# Comparative Study of Nanofiller-Reinforced Orthophthalic Polyester Composites: Mechanical, Surface, Tribological and Environmental Performance

**DOI:** 10.3390/ma19183989

**Published:** 2026-09-19

**Authors:** Dominik Stępka, Dagmara Słota, Karina Niziołek, Zuzanna Buchwald, Kinga Setlak, Dariusz Mierzwiński, Josef Jampilek, Katarzyna Haraźna, Agnieszka Sobczak-Kupiec

**Affiliations:** 1 CUT Doctoral School, Department of Materials Science, Faculty of Materials Engineering and Physics, Cracow University of Technology, 37 Jana Pawła II Av., 31-864 Krakow, Poland; 2 Department of Materials Science, Faculty of Materials Engineering and Physics, Cracow University of Technology, 37 Jana Pawła II Av., 31-864 Krakow, Poland; 3 Institute of Chemical Technology and Engineering, Poznan University of Technology, Berdychowo St. 4, 61-131 Poznań, Poland; 4 Department of Chemical Biology, Faculty of Science, Palacky University, Slechtitelu 27, 779 00 Olomouc, Czech Republic; 5 Department of Analytical Chemistry, Faculty of Natural Sciences, Comenius University, Ilkovicova 6, 842 15 Bratislava, Slovakia

**Keywords:** polymer composites, orthophthalic polyester resin, nanofillers, carbon nanotubes, halloysite, nanoclay, zinc oxide

## Abstract

Orthophthalic unsaturated polyester resins are widely used in engineering applications due to their favourable mechanical properties, low cost, and ease of processing; however, their long-term durability under environmental exposure remains a significant challenge. Therefore, this study investigates the effect of selected nanofillers on the mechanical, surface, and tribological properties of orthophthalic unsaturated polyester resin composites. Carbon nanotubes (CNTs), halloysite, Cloisite^®^ 30B, nanoclay, and zinc oxide (ZnO) were incorporated into the polyester matrix at concentrations of 0.1, 0.25, and 0.5 wt.%. The environmental stability of the developed composites was evaluated through incubation in buffer solutions with pH values of 4, 7, and 9. Changes in pH values, sample mass and surface morphology were assessed before and after exposure. In addition, accelerated ageing and soil burial tests were conducted to evaluate the stability of the materials under different environmental exposure conditions. Changes induced by environmental exposure were evaluated using mass measurements, surface roughness analysis, morphological observations, and mechanical characterisation, depending on the applied exposure procedure. The surface characteristics of the composites were investigated using contact angle measurements and scanning electron microscopy (SEM). Mechanical and functional performance was further evaluated through scratch resistance testing in accordance with PN-EN ISO 1518:2023 and pull-off adhesion measurements. Tribological behaviour was determined for selected ZnO- and nanoclay-modified composites by evaluating the coefficient of friction, linear wear, wear track morphology, and surface topography. Furthermore, the gross heat of combustion of selected materials was determined according to PN-EN ISO 1716:2018. The obtained results provide a comprehensive comparison of the effects of nanofiller type and concentration on the mechanical, surface, tribological, and environmental behaviorpolyester resin composites. The results demonstrate that the investigated nanofillers affect different aspects of composite performance and that their effectiveness depends on both nanofiller type and concentration, indicating that nanofiller selection should be tailored to the properties required for the intended application.

## 1. Introduction

In 2024, the global polyester resin market was valued at USD 8.50 billion and is expected to grow to USD 10.29 billion by 2029, with a projected compound annual growth rate (CAGR) of 3.9%. The main factors driving the growth of this market are rising demand in sectors such as construction, transport, electronics and electrical engineering [1]. Their industrial application is driven by their low cost, good chemical resistance, and ease of large-scale processing [2]. A specific example of resins is unsaturated polyester resins (UPR), which are among the most commonly used thermosetting resins in the composites industry. They are linear or slightly branched oligomers obtained by the polycondensation of glycols with saturated and unsaturated dicarboxylic acids or their anhydrides [3,4,5]. A characteristic feature of their structure is the presence of C=C double bonds, most commonly derived from maleic anhydride or fumaric acid [6]. The presence of these bonds enables their subsequent curing via a free-radical reaction with the initiator methyl ethyl ketone peroxide (MEKP) [7]. 

The popularity of polyester resins derives from their favourable ratio of performance characteristics to production costs, ease of processing, and good mechanical and chemical properties [8]. UPRs are widely used in the manufacture of pipes, tanks, technical infrastructure components, marine structures, transport components and construction products [9,10]. They play a particularly important role in continuous winding and textile-based composite manufacturing methods, where they serve as a matrix for various types of fibre reinforcements [11]. Despite their numerous advantages, the long-term use of polyester resin-based composites in environmental conditions can lead to the gradual degradation of the material [12]. The presence of the aforementioned ester bonds in the resin’s structure makes it susceptible to hydrolytic processes occurring during prolonged exposure to moisture and environments with fluctuating pH levels [13]. Furthermore, ultraviolet (UV) radiation and biological factors may also contribute to degradation [14,15]. These factors can cause changes in surface properties and a deterioration in mechanical parameters, resulting from water absorption and the hydrolysis of ester bonds. This affects the durability of products used in the natural environment in infrastructure and installation applications [16]. 

One of the most commonly used methods of modifying UPR, which can positively improve mechanical and strength properties, is the incorporation of nanofillers [17]. The development of nanotechnology is driving progress in the design of resin-based composites reinforced with nanoadditives/nanofillers, the most commonly used of which are nanofibres, nano-clay, metal oxides and carbon nanotubes. In 2025, the Asia-Pacific region was the largest market for polymer nanocomposites, and it is predicted that in the coming years, this region will see the fastest growth rate in the global polymer nanocomposites market [18,19]. Nanofillers are characterised by a very large specific surface area and a specific morphology; for this reason, they can influence the mechanical, tribological, thermal and barrier properties of composites, as well as increase their resistance to environmental degradation. However, the effectiveness of such modification depends on both the type of nanoadditive and its concentration, as well as the degree of dispersion within the polymer matrix [20,21]. Experimental studies reported in the scientific literature show that these effects depend to a large extent on both the physicochemical properties of the nanofiller and the environmental or loading conditions applied. Pączkowski et al. investigated unsaturated polyester composites containing 0.1–0.5% by mass of CNTs and demonstrated changes in their mechanical and thermomechanical properties, as well as in their response to accelerated ageing under UV radiation and to chemical exposure [22]. Importantly, the authors also noted that increasing the carbon nanotube content did not necessarily lead to a proportional improvement in the composite’s properties, which highlights the importance of the distribution of the nanofiller within the polymer matrix. The influence of the chemical composition of nanofillers on the mechanical and thermal properties of UPR-based composites has also been demonstrated for other nanoscale modifiers. Farshidfar et al. demonstrated that the mechanical and thermomechanical properties of graphene oxide/nano-clay/UPR nanocomposites were strongly influenced by the composition of the nanofillers and their surface chemistry, highlighting the role of polymer–filler interactions in determining the material’s final behaviour [23]. It has also recently been reported that carbon nanosystems incorporated into UPR simultaneously modify tensile strength, thermal stability, behaviour during the glass transition, and the elastic modulus during storage [24]. The aim of this study was to comparatively evaluate the influence of different nanofillers on the performance of orthophthalic UPR composites. Zinc oxide (ZnO), carbon nanotubes (CNTs), Cloisite^®^ 30B, halloysite, and nanoclay were selected as reinforcing nanomaterials and incorporated into the polyester matrix. ZnO is characterised by high chemical stability, good thermal resistance and the ability to absorb UV radiation. For this reason, it can help to limit photodegradation and ageing processes in materials used outdoors [25,26]. The potential of nanoscale fillers to modify the behaviour of UPRs under ageing conditions has also been demonstrated experimentally. UPR nanocomposites containing carbon nanolayers exhibited better protection against UV radiation and improved thermal properties, demonstrating that relatively low concentrations of nanofillers can significantly influence the response of the polyester matrix to UV exposure [27]. CNTs were selected as UPR modifiers because their high aspect ratio, exceptional mechanical strength, and high elastic modulus make them effective reinforcing agents even at relatively low concentrations. Their incorporation may enhance stress transfer within the polymer matrix and consequently improve the mechanical performance of UPR composites [28,29]. They are characterised by very high mechanical strength, a high modulus of elasticity, as well as excellent electrical and thermal conductivity. Even a small addition of CNTs can lead to a significant improvement in the mechanical properties of polymer composites [30,31]. Another modifier selected was Cloisite 30B, an organically modified montmorillonite belonging to the group of layered nanoclays. The literature indicates that the addition of Cloisite^®^ 30B may lead to improved mechanical properties, thermal stability and resistance to ageing processes. Furthermore, the layered structure of this material may limit the transport of moisture and degrading agents into the interior of the composite [32,33]. Compared with other nanofillers, halloysite is characterised by its relatively low production cost and high availability. It is a natural aluminosilicate that occurs most commonly in the form of hollow nanotubes. This morphology helps to improve the mechanical and tribological properties of composites. It has been shown that its presence can also increase the stiffness of the material and limit the development of microcracks during service [34]. The final nanomaterial selected for UPR modification in this study is nanoclay. Like Cloisite^®^ 30B, it has a layered structure and is characterised by a very high specific surface area, enabling effective interaction with the polymer matrix even at low concentrations. The incorporation of nanoclay can lead to an improvement in the material’s mechanical, thermal and barrier properties. Furthermore, it may limit the diffusion of water within the composite [35,36]. The selection of these nanofillers was based on their distinct physicochemical characteristics, morphologies, and expected functions within the UPR matrix. ZnO was selected primarily due to its chemical and thermal stability and UV-absorbing ability, which may contribute to improved resistance to environmental ageing [37,38]. CNTs were included as high-aspect-ratio reinforcing nanomaterials with the potential to enhance the mechanical performance of the resin at low concentrations [39,40]. Cloisite^®^ 30B and nanoclay were selected because their layered silicate structure can create a more tortuous diffusion pathway, thereby potentially limiting the penetration of moisture and other degrading agents into the polymer matrix [41]. Halloysite, in turn, combines a characteristic tubular morphology with relatively low cost and high availability and may contribute to improved mechanical and tribological properties [42]. Thus, the selected nanofillers represent different reinforcement mechanisms, enabling a systematic comparison of their effectiveness in modifying the properties and environmental durability of orthophthalic UPR composites. Although these nanofillers have been investigated individually in polyester-based composites, direct comparative studies evaluating nanofillers with different chemical compositions and morphologies within the same orthophthalic UPR matrix and under consistent experimental conditions remain limited. Therefore, the originality of the present study lies in the systematic comparison of five different nanofillers within a common experimental framework, allowing their effects on the properties and environmental durability of UPR composites to be directly compared. 

In this study, five nanofillers with different physicochemical characteristics and morphologies—zinc oxide (ZnO), carbon nanotubes (CNTs), Cloisite^®^ 30B, halloysite, and nanoclay—were selected for the modification of orthophthalic UPR. Therefore, the aim of this work was to investigate the influence of selected nanofillers on the mechanical, surface, tribological and environmental properties of UPR composites. Particular attention was given to the effect of nanofiller type and concentration on composite performance and stability under different environmental exposure conditions. The study included the evaluation of chemical structure and morphology, surface characteristics, compressive behaviour, qualitative response to scratching, pull-off behaviour, tribological performance, and environmental stability during buffer incubation, soil burial, and accelerated weathering. In addition, the gross heat of combustion was determined for selected materials. The surface-related measurements and environmental exposure tests were selected to provide complementary information on the behaviour of the investigated composites. Contact angle and surface free energy measurements were used to characterise the initial surface–liquid interactions of the materials, while surface roughness analysis enabled the assessment of topographical changes associated with environmental exposure. These measurements were complemented by abiotic ageing tests designed to evaluate the stability of the composites under different environmental conditions. Together, these analyses provide a comprehensive basis for relating the initial surface characteristics of the composites to their subsequent response to environmental exposure and for assessing the influence of nanofiller type and concentration on their overall environmental stability.

## 2. Materials and Methods

### 2.1. Materials

The polymer matrix used in this study was an unsaturated polyester resin (UPR), supplied by Ciech Sarzyna S.A. (Nowa Sarzyna, Poland). This resin is commonly employed in the production of pipes and tanks manufactured using the Continuous Filament Winding (CFW) process. According to the manufacturer, the resin exhibits a viscosity of approximately 205 mPa·s at 23 °C, a styrene monomer content of about 44 wt.%, and a flexural strength of approximately 86 MPa. The resin was used as received without further purification. The investigated nanofillers included nanoclay, halloysite, Cloisite^®^ 30B, multi-walled CNTs, and ZnO. Nanoclay (montmorillonite clay, CAS No. 1318-93-0) was purchased from Sigma-Aldrich (St. Louis, MO, USA). According to the manufacturer, the nanoclay consisted of montmorillonite modified with 34–45 wt.% dimethyl dialkyl amine, with an average particle size below 20 µm and a bulk density ranging from 200 to 500 kg·m^−3^. Halloysite nanoclay (CAS No. 1332-58-7) was purchased from Sigma-Aldrich (St. Louis, MO, USA). Cloisite^®^ 30B from BYK Additives (Shanghai, China) was a separate commercially available organically modified montmorillonite and was used as received. According to the supplier’s technical data, Cloisite^®^ 30B was modified with methyl, tallow, bis-2-hydroxyethyl, quaternary ammonium at a modifier concentration of 90 meq/100 g clay. Its particle size distribution was characterised by 10% of particles ≤ 2 µm, 50% ≤ 6 µm, and 90% ≤ 13 µm. Thus, although both the material designated as “nanoclay” and Cloisite^®^ 30B are organically modified montmorillonite-based fillers, they are distinct commercial materials with different organic surface treatments and physicochemical characteristics. Multi-walled carbon nanotubes (MWCNTs, CAS No. 308068-56-6, product No. 412988, Sigma-Aldrich, St. Louis, MO, USA) were employed as the carbon-based nanofiller. According to the manufacturer, the MWCNTs were produced by the arc-discharge method and supplied as an as-produced cathode deposit with a nominal outer diameter of 7–15 nm, a length of 0.5–10 µm, and an average number of 5–20 graphitic layers. The material contained >99% total carbon (TGA) and >7.5% MWCN1zTs and was specified by the manufacturer as catalyst-free. No surface functionalization was specified by the manufacturer. Zinc oxide (ZnO, CAS No. 1314-13-2) was supplied by Sigma-Aldrich (St. Louis, MO, USA). Composite fabrication was carried out using MEKP supplied by AkzoNobel (Amsterdam, The Netherlands) as the curing initiator, together with a cobalt octanoate accelerator. Prior to casting, the moulds were coated with a wax-based release agent (Paste Wax 34D, Abel Industrie) to facilitate specimen demoulding. For incubation studies buffer solutions with pH values of 4, 7, and 9 were supplied by Chempur (Piekary Śląskie, Poland). For wettability measurements, demineralised water and diiodomethane were used as probe liquids. The diiodomethane was purchased from Sigma-Aldrich (St. Louis, MO, USA). Demineralised water was obtained using a Hydrolab HLP 5SP demineralization system.

Detailed physicochemical characteristics of all investigated nanofillers, including their particle size distribution, specific surface area, morphology, elemental composition, and surface characteristics, were determined in our previous study [43]. The same commercially available nanofillers were used in the present work. 

### 2.2. Samples Preparation

To prepare the composites, 300 g of orthophthalic unsaturated polyester resin was measured out and poured into a plastic bucket. The resin was then thermally stabilised to a constant temperature of 23 °C. Next, 0.75 mL of a 4% cobalt octanoate solution, used as an accelerator, and a specified amount of the selected nanofillers were added. The selected nanoadditives were ZnO, nanoclay, halloysite, Cloisite^®^ 30B and CNTs. Then, the mixture was initially stirred with a hand-held stirrer and then mixed with an electric stirrer for 15 min at 850 rpm. The mixture was immersed in a water bath at 23 °C and subjected to sonication for 10 min using an ultrasonic cleaner (Ulsonix Cleaning Instruments, Berlin, Germany, PROCLEAN 10.0, 240 W) whilst being stirred. Then, 6 mL of commercial methyl ethyl ketone peroxide (MEKP; Butanox^®^ M-50, AkzoNobel, Amsterdam, The Netherlands), supplied as a solution in dimethyl phthalate with a total active oxygen content of 8.8–9.0%, was added as the curing initiator. The mixture was stirred again until homogeneous, and subsequently poured into moulds. Before use, the moulds were coated with a wax-based antistatic agent. The curing time for the samples was one hour, after which they were removed from the moulds and left undisturbed for 24 h to cure completely. At the end of the curing process, the samples were heated in an oven at 60 °C for a period of 1 h; during this time, the samples were loaded (with approximately 5 kg) to prevent deformation. Pure resin samples were obtained in a similar manner, omitting the stage of adding nanofillers. The procedure for sample preparation was analogous to that described earlier [44].

The nanofiller contents of 0.1, 0.25, and 0.5 wt.% were calculated relative to the initial mass of the polyester resin (300 g), corresponding to 0.30, 0.75, and 1.50 g of nanofiller, respectively. The unmodified resin was used as a reference material and designated as Pure Resin. Composite samples were identified according to the type and concentration of nanofiller. Carbon nanotube-reinforced composites were denoted as CNTs_0.1, CNTs_0.25, and CNTs_0.5; nanoclay-filled composites as Nc_0.1, Nc_0.25, and Nc_0.5; zinc oxide-filled composites as ZnO_0.1, ZnO_0.25, and ZnO_0.5; halloysite-filled composites as Hl_0.1, Hl_0.25, and Hl_0.5; and Cloisite^®^ 30B-filled composites as Cl_0.1, Cl_0.25, and Cl_0.5, where the numerical suffix indicates the nanofiller content expressed in weight percent.

### 2.3. Fourier Transform Infrared Spectroscopy Analysis

To characterise the chemical structure and identify the functional groups present in the materials, Fourier-transform infrared spectroscopy (FT-IR) was performed on the resin used as a reference sample and all prepared composites with nanofillers. The analysis was carried out to determine whether the incorporation of the nanofiller induced any chemical changes within the resin matrix or whether the nanofiller remained physically dispersed without forming new chemical bonds. FT-IR spectra were obtained using a Nicolet iS5 FT-IR spectrometer equipped with an iD7 ATR accessory (Thermo Scientific, Loughborough, UK). Measurements were performed in the spectral range of 4000–400 cm^−1^ at room temperature, using 32 scans per sample and a spectral resolution of 4 cm^−1^.

### 2.4. Incubation in Buffers

To assess the stability of the composites, they were incubated in aqueous solutions simulating various environmental conditions, with pH values of 4, 7 and 9. Composite samples weighing approximately 0.5 g were individually placed in sterile containers containing 50 mL of buffer solution, immersed in the corresponding buffer solution, and tightly sealed. Three independent replicates (*n* = 3) were prepared for each experimental condition. In parallel, blank controls containing 40 mL of each buffer solution without composite samples were prepared and maintained under the same experimental conditions. The containers with the samples were stored at a temperature of 22 °C in a POL-EKO ST 5 B SMART incubator (POL-EKO, Wodzisław Śląski, Poland). The incubation period lasted 14 days. During this time, the pH of the solutions was measured at intervals of 3, 5, 7, 10 and 14 days using a CX-705 multifunction metre (Elmetron, Zabrze, Poland). After this period, the samples were removed from the liquids, and allowed to dry under ambient conditions until a constant mass was reached, defined as the point at which successive mass measurements showed no appreciable change. The dried samples were then weighed using an EX324M analytical balance (OHAUS Europe GmbH, Nänikon, Switzerland) to determine the change in mass following incubation. The percentage mass change was calculated according to Equation (1), where m_0_ is the initial and m_f_ is the final mass of composite.

(1) $$m=\frac{m_{f}-m_{0}}{m_{0}}\cdot 100\%$$

### 2.5. Wettability and Surface Free Energy

An analysis of surface wettability was carried out using the resting drop method. A See System goniometer manufactured by Advex Instruments (Brno, Czech Republic) was used for this purpose. The procedure complied with the PN-EN ISO 19403-2:2025 standard [45]. The contact angle of the samples was assessed for two different liquids: distilled water (a polar liquid) and diiodomethane (a non-polar liquid). For each test liquid, three individual 10 µL drops were deposited at three different positions on the smooth surface of each sample using an automatic pipette. The measurement positions were selected on smooth surface areas located at least 10 mm from the sample edges. The drop image was acquired immediately after deposition using a digital camera. Each drop was deposited at a new position to account for local variations in surface properties. The shape of the drop was recorded with a digital camera, transferred to a computer for magnification, and then analysed using See System 7.6 software. Measurements at different positions on each sample were used to account for local surface heterogeneity.

Surface free energy (SFE) and its dispersive and polar components were subsequently determined using the Owens–Wendt (OW) method based on the measured contact angles for distilled water and diiodomethane. The total surface free energy was calculated as the sum of its dispersive and polar components according to Equations (2)–(4):

(2) $$\gamma_{s}=\gamma_{s}^{D}+\gamma_{s}^{P}$$

(3) $$\sqrt{\gamma_{s}^{D}}=\frac{\gamma_{d}\left(\cos\theta_{d}+1\right)-\sqrt{\frac{\gamma_{d}^{P}}{\gamma_{w}^{P}}}\gamma_{w}\left(\cos\theta_{w}+1\right)}{2\left(\sqrt{\gamma_{d}^{D}}-\sqrt{\frac{\gamma_{d}^{P}\gamma_{d}^{D}}{\gamma_{w}^{P}}}\right)}$$

(4) $$\sqrt{\gamma_{s}^{P}}=\frac{\gamma_{w}\left(\cos\theta_{w}+1\right)-2\sqrt{\gamma_{s}^{D}\gamma_{w}^{D}}}{2\sqrt{\gamma_{w}^{P}}}$$

where

$\gamma_{s}$—total surface free energy of the sample,

$\gamma_{s}^{D}$—dispersive component of the sample surface free energy,

$\gamma_{s}^{P}$—polar component of the sample surface free energy,

$\gamma_{d}$—total surface tension of diiodomethane (50.8 mN m^−1^),

$\gamma_{d}^{D}$—dispersive component of the surface tension of diiodomethane (48.5 mN m^−1^),

$\gamma_{d}^{P}$—polar component of the surface tension of diiodomethane (2.3 mN m^−1^),

$\gamma_{w}$—total surface tension of water (72.8 mN m^−1^),

$\gamma_{w}^{D}$—dispersive component of the surface tension of water (21.8 mN m^−1^),

$\gamma_{w}^{P}$—polar component of the surface tension of water (51.0 mN m^−1^),

$\theta_{d}$—contact angle of diiodomethane,

$\theta_{w}$—contact angle of water.

### 2.6. Microscopic Analysis

#### 2.6.1. SEM

Scanning electron microscopy (SEM) was employed to investigate the surface morphology and fracture surfaces of the composites, both before and after the accelerated ageing tests. The observations were performed using a JEOL JSM-IT200 scanning electron microscope (JEOL Ltd., Tokyo, Japan) operating under low-vacuum conditions at an accelerating voltage of 10 kV. Micrographs were acquired at a magnifications of 100× and 250×. Prior to SEM analysis, both the specimen surfaces and the fracture surfaces were coated with a thin layer of gold using a sputter coater (DII-29030SCTR, JEOL, Tokyo, Japan) to improve electrical conductivity and image quality. The obtained micrographs were used to assess the morphology of the composites, and to identify possible local morphological heterogeneities, structural defects, and filler agglomerates. No quantitative assessment of nanofiller dispersion was performed.

#### 2.6.2. Optical Microscopy and Surface Roughness Analysis

Digital imaging and surface topography analysis were performed using a high-precision Keyence VHX-7000 4K digital microscope (Keyence Corporation, Osaka, Japan). Images were recorded with a resolution of 4000 × 3000 pixels using the HDR and depth-composition functions, which improved the visualisation of low-contrast features and enabled the reconstruction of surfaces exhibiting considerable height variations. For detailed surface analysis, representative areas were examined at 500× magnification, corresponding to a field of view of approximately 592 × 443 µm. The integrated measurement software was used to generate two-dimensional images, three-dimensional surface topography maps and line-height profiles. For specimens before incubation, after incubation in buffer solutions (pH 4, 7, and 9), and after soil burial, the Ra and Rz roughness parameters were determined, and representative three-dimensional surface topography maps and corresponding roughness profiles were obtained. Surface texture was quantitatively characterised using both areal and profile roughness parameters, including the arithmetic mean height, Sa, and the arithmetic average profile roughness, Ra, together with the remaining height and roughness parameters provided by the measurement software for specimens after aging materials. Multiple line profiles were recorded at different positions across each analysed surface to account for its spatial heterogeneity.

### 2.7. Thermogravimetric Analysis

Thermogravimetric analysis (TGA) was performed using an STA Regulus 2500 simultaneous thermal analyser (NETZSCH-Gerätebau GmbH, Selb, Germany). The measurements were conducted under a nitrogen atmosphere, with flow rates of 50 mL/min for the active gas and 20 mL/min for the purge gas, over a temperature range of 25–600 °C at a heating rate of 10 °C/min. The samples were subsequently held at 600 °C for 5 min, after which the atmosphere was changed to an oxidising atmosphere using synthetic air. The samples were then combusted at 600 °C for an additional 15 min, with a synthetic-air flow rate of 50 mL/min and a nitrogen purge flow rate of 20 mL/min. At least three independent measurements were performed for each material.

### 2.8. Differential Scanning Calorimetry

Differential scanning calorimetry (DSC) was performed using a NETZSCH DSC 3500 Sirius instrument (NETZSCH-Gerätebau GmbH, Selb, Germany). Samples weighing 10–20 mg were cut from the prepared specimens and placed in sealed aluminium crucibles. Measurements were conducted under a nitrogen atmosphere at a gas flow rate of 20 mL/min. The samples were analysed over a temperature range from −50 to 250 °C using a heating–cooling–heating programme. Both heating steps and the intermediate cooling step were performed at a rate of 10 K/min. The first heating cycle was used to evaluate the initial thermal state of the materials, including their thermal history and possible residual curing effects, whereas the second heating cycle was used to determine the thermal transitions after eliminating the previous thermal history of the samples.

### 2.9. Determination of Gross Heat of Combustion

The gross heat of combustion (PCS) of the prepared composites was determined in accordance with PN-EN ISO 1716:2018-08 [46]. The method is intended for the evaluation of the potential contribution of construction products to fire and is commonly used as part of the assessment procedure for reaction to fire classifications. The test was performed using a bomb calorimeter. A small specimen of the material was placed inside the combustion vessel, which was subsequently filled with pure oxygen at a pressure of approximately 30 bar. The specimen was then completely combusted under controlled conditions, and the heat released during combustion was determined calorimetrically. The gross heat of combustion was calculated and expressed in MJ·kg^−1^. For this study, the Nc_0.1 sample was selected as the formulation exhibiting the most favourable properties and was compared with the pure resin. For each material, three independent measurements were performed, and the results were reported as the mean value with the corresponding standard deviation.

### 2.10. Scratch Resistance Test

The scratch resistance test was carried out using the constant-load method. The measurement was performed on a series of nanoclay and ZnO samples using a scratch tester (serial number 5201709006) located at the Anti-Corrosion Laboratory of Tenslab S. z o.o. (Gdańsk, Poland). A PosiTector DPM thermohygrometer (S/N 817245), equipped with a probe (S/N 331597), was used to control the environmental conditions. The scratches were made using an HM1.0 scratch tester, applying a load of 20 N under conditions of 51% RH and an ambient temperature of 22 °C. The test was carried out in accordance with the PN-EN ISO 1518:2023 standard [47]. The material’s resistance was assessed on the basis of observations of the scratch marks produced under the applied load. The scratch test was used as a qualitative assessment of the surface response to scratching under defined loading conditions; scratch width, scratch depth, and critical load were not quantitatively determined.

### 2.11. Pull-Off Strength Measurements

Pull-off tests were performed to compare the resistance of the investigated nanocomposites to tensile loading applied perpendicular to the specimen surface and to assess changes in their surface-layer integrity resulting from nanofiller incorporation. The measurements were carried out using a PosiTest AT-A pull-off adhesion tester (DeFelsko Corporation, Ogdensburg, NY, USA). Circular aluminium dollies with a diameter of 20 mm were bonded to the specimen surfaces using a two-component epoxy adhesive. The dollies were positioned approximately 20 mm from the specimen edge to minimise edge effects. After bonding, the specimens were stored at 22 °C for 24 h to allow the adhesive to cure. Following the curing period, the dollies were subjected to tensile loading perpendicular to the specimen surface until failure occurred. The maximum pull-off stress was recorded and expressed in MPa. Three independent measurements were performed for each material variant.

After testing, the failure mode was determined by visual examination of the dolly and specimen surfaces. Failure was classified as adhesive when separation occurred at the adhesive–composite interface or cohesive when fracture occurred within the composite material.

### 2.12. Compressive Strength

Compression tests were carried out using a universal testing machine (MTS Criterion Model 43, MTS Systems Corporation, Eden Prairie, MN, USA) at ambient temperature. Square specimens with dimensions of 10 × 10 × 3 mm (length × width × height) were tested. Three independent specimens (*n* = 3) were tested for each material variant. Tests were carried out for composites both before and after the ageing test. The tests were performed at a loading speed of 10 mm/min. The compressive strength was determined as the maximum compressive stress (peak stress) recorded during the test. The applied load, compressive stress, and compressive modulus were recorded and analysed using MTS TestSuite™ software (version 1.0).

### 2.13. Tribological Testing 

Tribological tests were performed using a T-17 tribological tester operating in a linear reciprocating motion configuration. The pure resin was used as the reference material, while Nc_0.1 and ZnO_0.1 were selected as representative nanocomposites containing two chemically and morphologically different inorganic nanofillers. The test methodology was based on the principles of ASTM F732 [48].

The tribological pair consisted of a stationary pin made of 100Cr6 bearing steel pressed against a composite plate performing reciprocating motion. The tests were carried out under distilled water lubrication at a normal load of 225 N. The reciprocating motion was applied with a stroke length of 10 mm and a frequency of 1 Hz. Each test was performed for 5000 cycles, and a minimum of three repetitions was conducted for each material variant. During testing, the friction force was continuously monitored and used to calculate the coefficient of friction (CoF). In addition, the linear wear of the tribological system was determined. The obtained results were used to evaluate the influence of nanofiller incorporation on the friction and wear behaviour of the polyester composites.

Following the tribological tests, the wear tracks were examined using a Nikon MM-40/L3FA optical microscope equipped with the MultiScanBase v.8.08 image acquisition and processing system. The microscope enabled detailed observation of the wear scars using the Extended Focus Imaging (EFI) function, which provides enhanced depth of field for irregular surfaces. Selected specimens were further characterised using a Talysurf CCI white-light interferometric microscope (Taylor Hobson, Leicester, UK). Surface topography measurements were performed with a resolution of 1024 × 1024 measurement points. The acquired data were analysed using Talysurf CCI and TalyMap Platinum software to determine selected surface texture parameters, including roughness, waviness, amplitude distribution, bearing area curves, and surface volume characteristics. These analyses were performed to identify wear mechanisms and assess changes in surface geometry induced by the tribological interaction.

### 2.14. Assessment of Composite Stability 

#### 2.14.1. Soil Burial Test

In order to assess the stability of the composites, samples of material weighing approximately 5 g were buried in the soil at a depth of half a metre for a period of 30 days. Three independent replicates (*n* = 3) were used for each investigated material. The experiment was conducted outdoors under ambient environmental conditions; therefore, the temperature was not actively controlled or continuously recorded during the exposure period. The soil moisture content was 11.78 ± 0.58%, as determined using a RADWAG MA 50.R moisture analyser (Radom, Poland). The soil pH was 7.156 ± 0.001. This value was determined in accordance with ISO 10390 regarding soil quality by preparing a soil suspension in 1 mol/L KCl [49]. The pH of the suspension was measured using a CX-705 multifunction metre (Elmetron, Zabrze, Poland). The microbiological activity and microbial composition of the soil were not determined in the present study.

After the 30-day exposure period, the samples were retrieved from the soil and carefully rinsed with distilled water to remove adhering soil particles. The cleaned samples were subsequently allowed to dry under ambient conditions until a constant mass was reached, defined as the point at which successive mass measurements showed no appreciable change. The samples were then weighed using an EX324M analytical balance (OHAUS Europe GmbH, Nänikon, Switzerland). The percentage change in mass was calculated in accordance with Equation (1). The samples were subsequently analysed using a VHX-7000 4K digital microscope equipped with three-dimensional imaging and surface profiling capabilities (KEYENCE, Osaka, Japan), according to the procedure described in Section 2.6.2.

#### 2.14.2. Short-Term Accelerated Abiotic Weathering Test

Based on the results of the physicochemical, surface, thermal, and mechanical screening, the following formulations were selected for the short-term accelerated weathering experiment: Nc_0.25, Nc_0.5, Hl_0.5, CNTs_0.1, and ZnO_0.1, together with the neat resin used as a reference. Experiments were conducted using a Q-UV SPRAY accelerated weathering chamber (Q-LAB, Grand Rapids, MI, USA). The applied procedure was designed to simulate the combined effects of solar radiation, rainfall, and moisture condensation under controlled laboratory conditions. Ultraviolet exposure was provided by UV-A 340+ fluorescent lamps, which closely reproduce the short-wavelength region of natural sunlight between 295 and 365 nm. All investigated nanocomposite formulations were subjected to the accelerated ageing procedure. Specimens representing each material variant were removed from the chamber after 100 h of exposure. The weathering programme was performed according to ASTM G154 [50], Cycle 7, and consisted of the following sequential stages: 8 h of UV irradiation at an irradiance of 1.55 W m^−2^ nm^−1^ and a temperature of 60 °C, followed by 15 min of water spraying at 30 °C, and subsequently a condensation stage lasting 225 min at 50 °C. The entire exposure cycle was repeated continuously until the required ageing time was reached [51]. After completion of the test, the specimens were carefully secured and stored until further characterisation. The materials were subsequently subjected to analyses aimed at determining their structural parameters and surface profiles, evaluating their morphology, and identifying changes induced during the ageing process. Hardness and compression tests were also performed to assess the effect of weathering on the mechanical properties of the nanocomposites. Detailed descriptions of the applied characterisation methods are provided in the previous described sections. 

### 2.15. Statistical Analysis

Statistical differences between the investigated groups were evaluated using one-way analysis of variance (ANOVA). Differences were considered statistically significant at * *p* < 0.05, ** *p* < 0.01, and *** *p* < 0.001. Statistical analyses were carried out using OriginPro 2019 software (OriginLab Corporation, Northampton, MA, USA). 

## 3. Results

### 3.1. Fourier Transform Infrared Spectroscopy Analysis 

**Figure 1:**
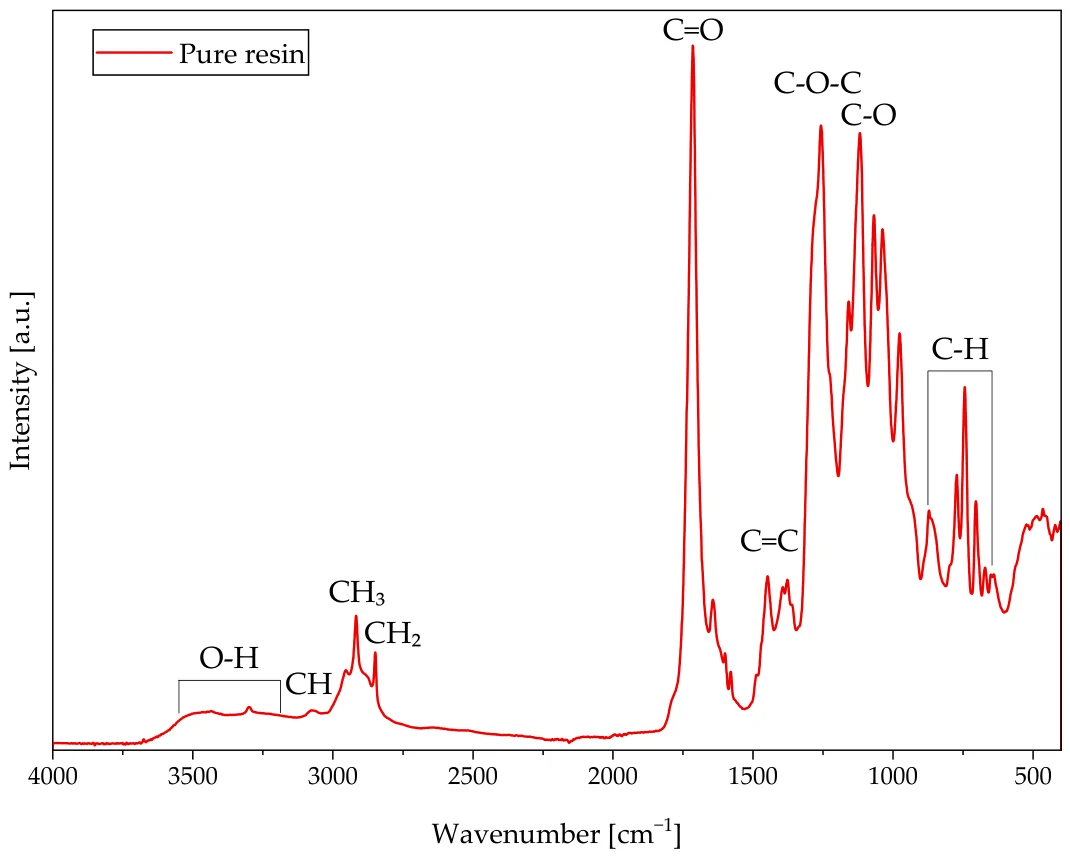
The FTIR spectrum of an cured neat unsaturated polyester resin, highlighting the functional groups.

Figure 1 presents the FTIR spectrum for the pure resin. At a wavelength of 1715 cm^−1^, one of the main bands characteristic of UPR was observed; this has been attributed to the stretching vibrations of the C=O carbonyl groups present in the ester bonds of the polyester chain. Other characteristic spectra for the polyester matrix were observed at 1118 cm^−1^ and 1257 cm^−1^, corresponding to the stretching vibrations of the C-O and C-O-C bonds in the ester groups. Bands in the range of approximately 880–630 cm^−1^ may be associated with deformation vibrations of C-H bonds. Signals for skeletal C=C vibrations of aromatic rings were observed at 1640 and 1446 cm^−1^. A weak band at 3083 cm^−1^ may additionally include C-H vibrations of aromatic rings [52,53]. At wavelengths of 2847 cm^−1^ and 2918 cm^−1^, bands associated with the stretching vibrations of C-H bonds in the aliphatic -CH_2_ and -CH_3_ groups are visible [54]. A broad, weaker band in the range of approximately 3500–3200 cm^−1^ can be attributed to the stretching vibrations of O-H groups. This may result from the presence of terminal hydroxyl groups in the polyester or from trace moisture [55]. 

**Figure 2:**
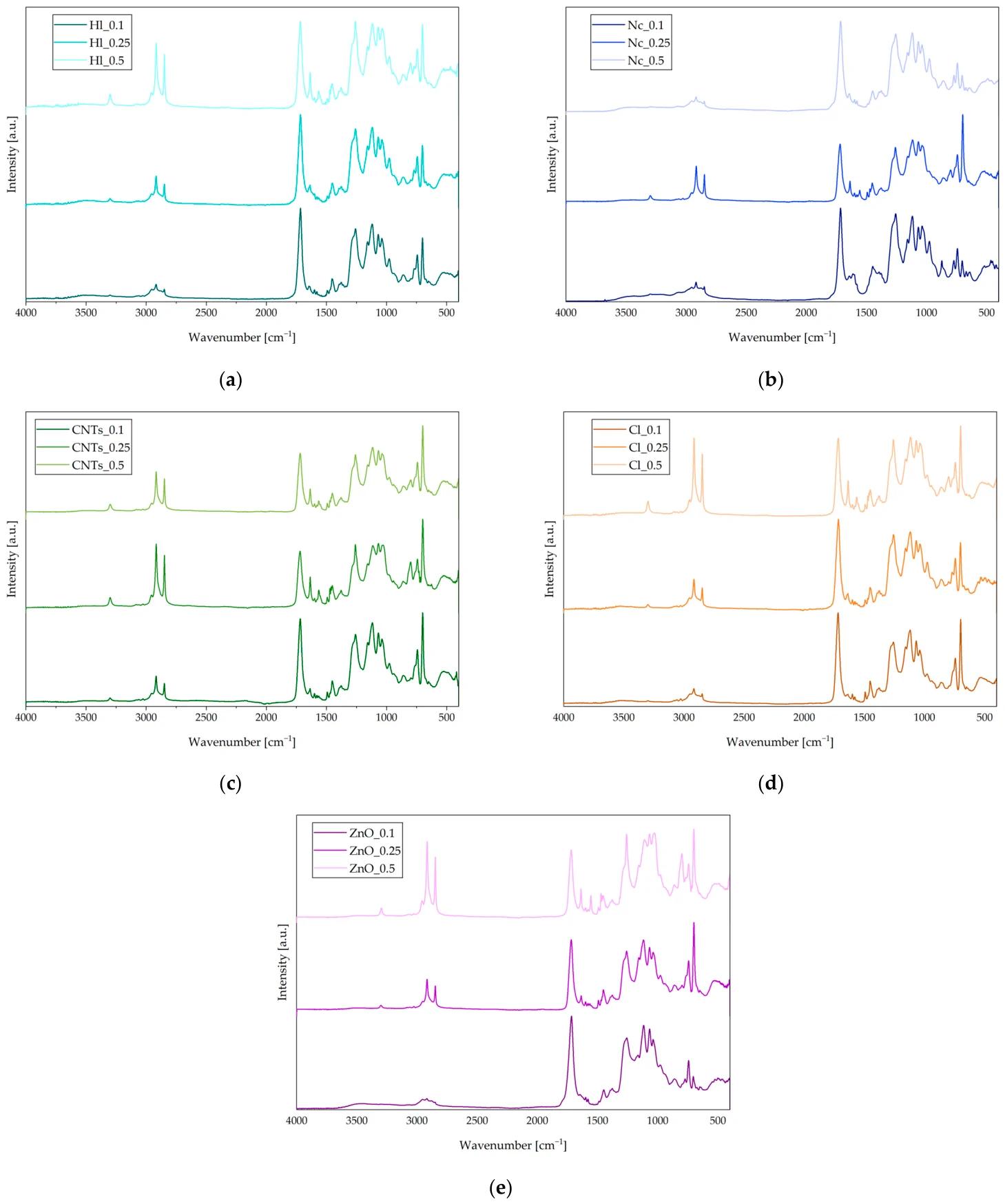
FTIR spectra of polyester resin composites containing nanofillers: (**a**) halloysite; (**b**) nanoclay; (**c**) CNTs; (**d**) Cloisite^®^ 30B; (**e**) ZnO.

The FTIR spectra for composites with nanofillers are shown in Figure 2. It was observed that the spectra of the composites are dominated by the matrix and exhibit the typical profile of cured polyester resin. Considering the relatively low nanofiller contents used in this study (0.1–0.5 wt.%), the contribution of the fillers to the overall spectra is expected to be limited, and some filler-related bands may overlap with the intense absorption bands of the polyester matrix. 

For composites containing halloysite (Figure 2a), the bands characteristic of this material at 3695 and 3620 cm^−1^ were not clearly observed [56]. This may be due to its low content and the dominant contribution of the polyester matrix to the recorded spectra. The contribution of halloysite should be visible in the range of approximately 1100–900 cm^−1^, but this region overlaps with the very intense C-O-C and C-O vibrations of the resin’s ester groups. However, in the case of Hl_0.5, slightly more pronounced bands were observed in the region around 1100–900 cm^−1^ and at lower wavenumbers, which may result from a greater contribution from the vibrations of the aluminosilicate framework [57]. Nevertheless, because of the substantial overlap of absorption bands in this region, these spectral differences cannot be unequivocally attributed to specific chemical interactions between halloysite and the polyester matrix. In the spectra of nanoclay (Figure 2b), certain differences are visible in the 3500–3000 cm^−1^ region. In particular, for Nc_0.5, a broader signal is observed in the -OH group region. This may be related to the presence of hydroxyl groups and adsorbed moisture typical for hydrophilic clay [58]. In the range of 1100–1000 cm^−1^, the bands become broad and complex. This is a region common to Si-O in the nanoclay, but also to C-O-C and C-O in the polyester resin [59]. Consequently, the observed differences in this spectral region cannot be unequivocally assigned to the formation of chemical bonds between the nanoclay and the polymer matrix. In the case of CNT composites (Figure 2c), bands characteristic of cured polyester resin are observed. No new absorption bands were observed with increasing CNT content. This is primarily due to the low activity of the unmodified graphite structure in the infrared region [60] and may additionally be related to the relatively low CNT concentrations used in the investigated composites. However, the absence of new absorption should not be interpreted as definitive evidence for the absence of chemical or interfacial interactions. Rather, no clear spectroscopic evidence of the formation of new functional groups or chemical bonds between CNTs and the polyester matrix was obtained under the applied experimental conditions. Possible interactions at the CNT-polymer interface may be physical in nature or may result in spectral changes below the sensitivity of the applied FTIR analysis. In the case of Cloisite^®^ 30B (Figure 2d), two bands in the range of 2950–2850 cm^−1^ are more clearly discernible for Cl_0.5. These may originate from the aliphatic groups of the organic Cloisite^®^ 30B. However, as demonstrated above, the resin itself also exhibits intense -CH_2_ and -CH_3_ bands in this range. These bands are therefore not unique to Cloisite^®^ 30B. As with nanoclay, the Si-O contribution should be present in the range of approximately 1100–1000 cm^−1^, but it overlaps with the ester vibrations of the resin [61,62]. Therefore, no distinct additional absorption bands that could be unequivocally assigned to the formation of new chemical bonds between Cloisite^®^ 30Band the polyester matrix were identified. Figure 2e presents the spectra for ZnO composites. ZnO is characterised by Zn-O lattice vibrations, typically observed below 600 cm^−1^ [63]. However, the polyester matrix also exhibits bands in the 700–400 cm^−1^ range; therefore, it is not possible to clearly identify the signal from the oxide. This limitation is particularly relevant considering the low ZnO concentrations used in the present study. Consequently, the obtained FTIR spectra do not provide sufficient evidence to determine whether specific chemical interactions occur between ZnO and the polyester matrix.

The FTIR spectra of all investigated nanocomposites remained predominantly characteristic of the cured polyester matrix, with only minor differences observed in selected spectral regions. It should be emphasised that FTIR spectroscopy alone cannot unequivocally confirm or exclude the formation of chemical bonds between the nanofillers and the polymer matrix, particularly at the low filler concentrations investigated in this study. Potential filler-related absorption bands may exhibit low intensity, overlap with the characteristic bands of the polyester matrix, or appear only as subtle changes in band position, shape, or intensity. Moreover, such changes may result from physical interactions, differences in the local chemical environment, or filler dispersion rather than from the formation of covalent bonds. Therefore, the FTIR results obtained in the present study are interpreted as indicating that no distinct spectroscopic evidence of new bond formation was detected, rather than as proof of either the presence or absence of chemical bonding at the polymer–nanofiller interface.

### 3.2. Stability Assesment 

#### 3.2.1. Changes in Buffer Solution pH

**Figure 3:**
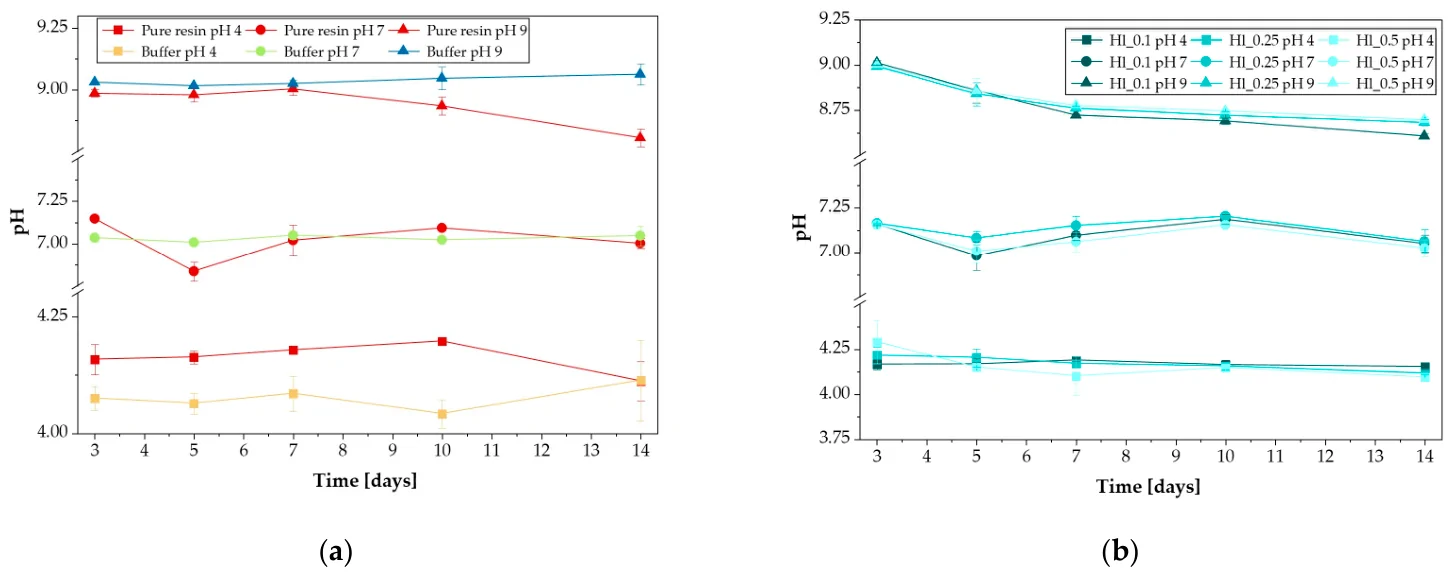

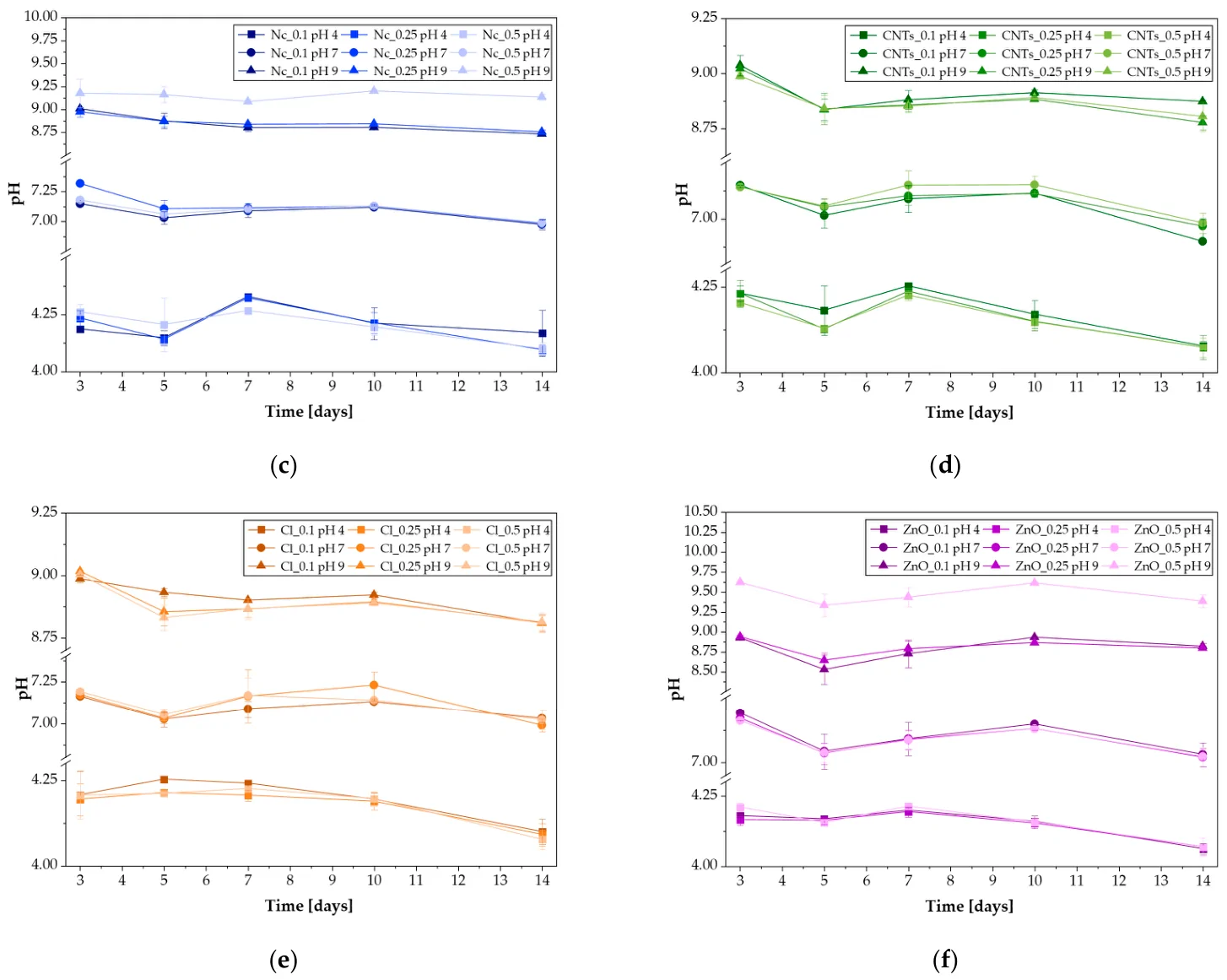
Changes in the pH of buffer solutions (pH 4, 7, and 9) during the incubation of the neat unsaturated polyester resin and nanofiller-modified composites. (**a**) Pure resin, (**b**) Halloysite, (**c**) Nanoclay, (**d**) CNTs, (**e**) Cloisite^®^ 30B, and (**f**) ZnO (*n* = 3).

The potentiometric measurements obtained during the 14-day incubation are presented in Figure 3. The analysis carried out showed that the samples under investigation did not cause any sudden changes in the pH of the buffer solutions. The pH values remained close to their initial values, indicating only minor changes in the acid–base conditions of the incubation media. The greatest relative changes were usually observed during the first few days of exposure, whilst in the later period the pH values stabilised or changed only slightly. For pure resin, the changes in pH were minor in each of the three environments. In the acidic buffer, the pH initially remained at around 4.15, and then, after 14 days, fell to around 4.1. In the neutral solution, an initial drop in pH was observed, followed by a partial return towards the initial value. In the alkaline environment, the pH remained close to 9, although a slight decrease was observed towards the end of the incubation period. These results indicate that the cured polyester resin itself did not significantly affect the chemical stability of the solutions, and the changes observed were minor. In the case of composites containing halloysite, the changes were similar for all three concentrations of the nanoadditive. However, in an alkaline environment, a gradual decrease in pH from 9.0 to 8.6 was recorded. This may indicate interactions between the halloysite-containing composite and the incubation medium; however, the scale of these changes was small. Composites containing nanoclay exhibited slightly greater variability, particularly in an acidic environment. For the Nc_0.1, Nc_0.25 and Nc_0.5 samples, transient fluctuations in pH were observed, particularly between the 5th and 7th days of incubation. After 14 days, however, the pH values remained close to their initial levels. In an alkaline environment, a slight, gradual decrease in pH was observed, whilst in a neutral environment the changes did not exceed a few tenths of a pH unit. These minor variations may reflect interactions between the nanoclay-containing composite and the incubation medium. For compositions modified with CNTs, the course of the changes was more regular. No clear dependence on CNT concentration was observed, indicating that the presence of nanotubes in the range of 0.1–0.5 wt.% did not cause significant chemical changes in the incubation environment. Compositions containing Cloisite^®^ 30Bexhibited slight fluctuations in pH throughout the study period. The small amplitude of these changes may indicate that the pH of the incubation medium remained relatively stable throughout the exposure period. The greatest differences between the various concentrations were observed in the case of composites containing ZnO, particularly in the alkaline solution. For the ZnO_0.5 sample, the pH remained higher than for the other variants, at around 9.3–9.6. This may indicate that the surface properties of ZnO influence the acid–base equilibrium of the solution. In acidic and neutral buffers, the differences in concentration were significantly smaller, and the pH values remained close to their initial levels. It should be noted that the observed pH variations alone cannot be interpreted as evidence of the release or leaching of specific substances from the composites, as no complementary chemical analyses of the incubation solutions were performed.

#### 3.2.2. Changes in Mass Following Different pH Exposures and Soil Burial Tests

Changes in the mass of samples following incubation in buffer solutions and exposure to soil provide indirect information on the composites’ resistance to environmental influences and their ability to absorb water and other environmental components. Analysis of the results (Table 1) revealed that all the materials tested underwent a change in mass following a 14-day incubation in buffers of varying pH. However, the extent of these changes depended on both the type of nanoadditive used and the pH of the environment. In most cases, the greatest mass gain was observed following exposure to an acidic solution at pH = 4. Particularly high values were observed for the CNTs_0.1 and Nc_0.5, 0.903 ± 0.104% and 0.790 ± 0.166%, respectively. It can be assumed that the acidic environment facilitated more intense water penetration into the material. This may be due to the presence of polar ester groups characteristic of unsaturated polyester resins, which are capable of interacting with water molecules. In neutral and alkaline environments, changes in mass were generally smaller. For most of the materials, a gradual decrease in mass gain was observed as the pH increased. This trend was particularly evident for composites containing nanoclay and CNTs. At the same time, not all materials exhibited identical behaviour. For example, Hl_0.5 exhibited the greatest mass gain in a neutral environment, suggesting that the sorption mechanism depends not only on the pH of the environment but also on the chemical nature and morphology of the nanofiller used. When analysing the effect of the type of nanoadditive, it can be observed that composites containing Cloisite^®^ 30Band ZnO exhibited relatively small changes in mass regardless of the pH value. This may indicate a beneficial effect of these nanoadditives in limiting the transport of liquid into the interior of the material. In the case of Cloisite^®^ 30B, this effect can be attributed to the layered structure of the organically modified clay, which increases the tortuosity of the diffusion path of water molecules. A similar phenomenon is described in the literature for polymer nanocomposites containing montmorillonite. However, it is important to note that the differences observed between the samples were relatively small.

Similar trends were observed for the results obtained after 30 days’ exposure of the samples to soil. A positive change in mass was recorded for some of the materials, indicating the absorption of moisture present in the soil environment. The greatest increase in mass was observed for the CNTs_0.5 (2.560 ± 0.331%), whilst for most of the other materials the values did not exceed 0.5%. Such a significant increase in mass may indicate that this composite is more susceptible to moisture absorption. Interesting results were also obtained for Cl_0.1 and Cl_0.5, where a negative change in mass was observed following the soil test. This may indicate the partial removal of degradation products from the material’s surface, the leaching of less firmly bound composite fragments, or local damage to the surface layer during exposure to the soil environment. However, this phenomenon requires further analysis, as the change in mass alone does not allow for an unambiguous determination of the mechanism responsible for the observed loss of material.

### 3.3. Wettability and Surface Free Energy

**Figure 4:**
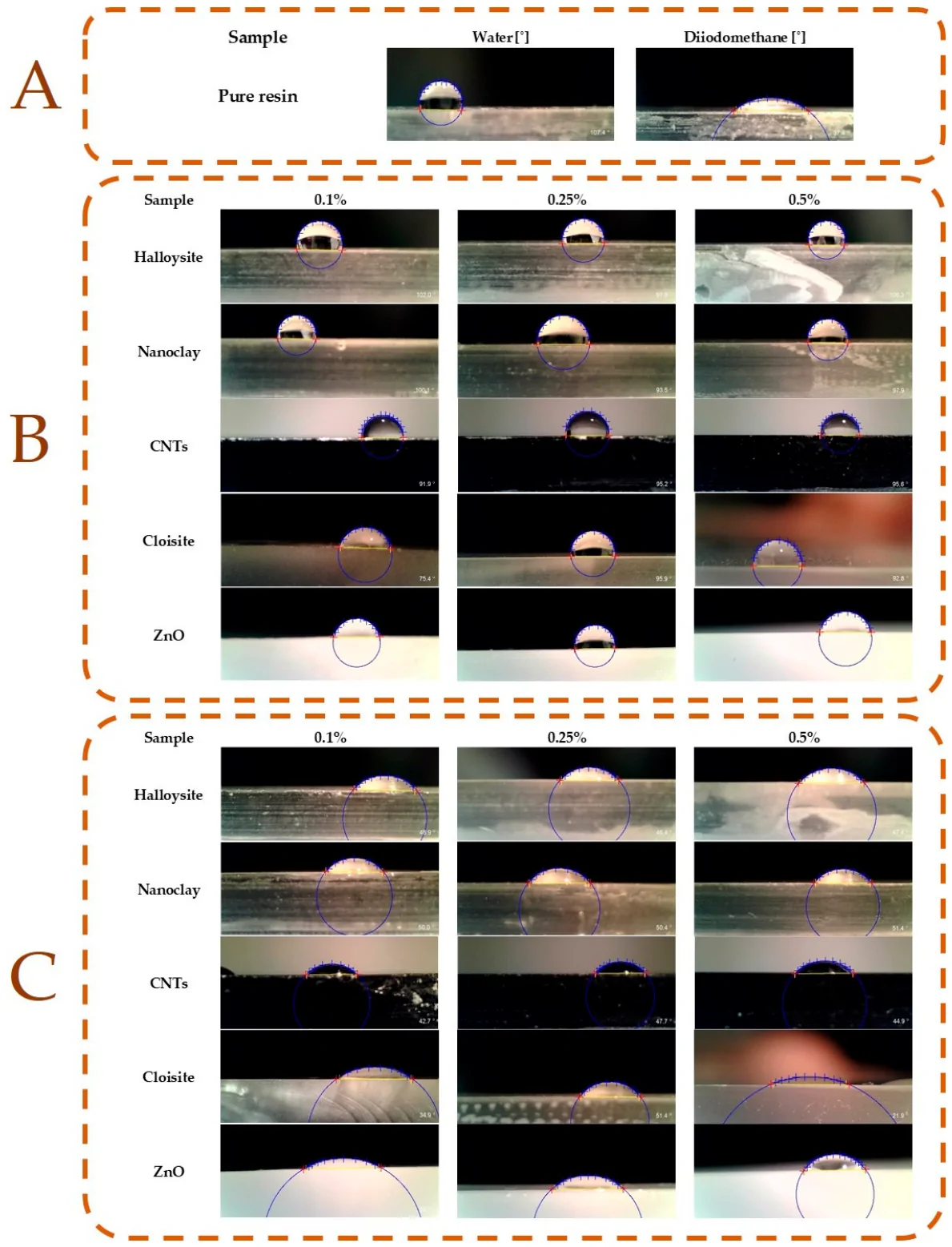
Representative images of contact angle measurements performed on neat unsaturated polyester resin and nanofiller-modified composites. (**A**) Neat unsaturated polyester resin. (**B**) Water contact angle images. (**C**) Diiodomethane contact angle images. Nanofiller concentrations are indicated in the columns (0.1, 0.25, and 0.5 wt.%), while the nanofiller type is indicated in the row.

Representative images of water and diiodomethane droplets on the surface of the neat resin and nanofiller-modified composites are presented in Figure 4, while the corresponding contact angle values and calculated surface free energy parameters are summarised in Table 2.

The unmodified polyester resin exhibited a water contact angle of 102.95 ± 3.31°, indicating a predominantly hydrophobic surface. The incorporation of nanofillers affected the wetting behaviour to different extents, depending on both the filler type and concentration. As can be observed in Figure 4, the morphology of the sessile droplets varied noticeably among the investigated composites, particularly for Cloisite^®^ 30B- and ZnO-modified samples, reflecting differences in surface wettability. The clay-based fillers (halloysite and nanoclay) produced only minor changes in water contact angle, with values remaining close to those of the reference material (95–105°). Similarly, composites containing CNTs exhibited only a moderate decrease in water contact angle, reaching approximately 93–96°. In contrast, Cloisite^®^ 30B and ZnO caused the most pronounced changes in surface wettability. The lowest water contact angles were observed for Cloisite^®^ 30B (78.72 ± 3.41° for 0.1 wt.% and 86.71 ± 5.73° for 0.5 wt.%) and ZnO (78.54 ± 1.14° for 0.1 wt.% and 77.75 ± 1.69° for 0.5 wt.%), indicating a substantial increase in surface hydrophilicity compared with the neat resin.

Similar trends were observed for diiodomethane contact angle measurements (Figure 4C), although the magnitude of changes depended on the nanofiller. The lowest diiodomethane contact angles were measured for Cloisite^®^ 30B containing 0.5 wt.% (22.06 ± 2.67°) and ZnO containing 0.1 wt.% (29.42 ± 1.61°), whereas halloysite-, nanoclay-, and CNT-modified composites exhibited values comparable to or slightly higher than those of the reference resin. 

The calculated surface free energy ranged from 33.66 ± 0.83 to 47.77 ± 1.08 mN m^−1^ (Table 2). The neat resin exhibited a total surface free energy of 42.12 ± 2.01 mN m^−1^. Among all investigated fillers, Cloisite^®^ 30B (0.5 wt.%) resulted in the highest surface free energy (47.77 ± 1.08 mN m^−1^), closely followed by ZnO at 0.1 wt.% (44.74 ± 0.62 mN m^−1^). Conversely, nanoclay produced the lowest surface free energy, decreasing to 33.66 ± 0.83 mN m^−1^ at 0.5 wt.%. Analysis of the individual surface free energy components revealed that the observed changes were governed primarily by the dispersive component ($\gamma_{s}^{D}$), whereas the polar component ($\gamma_{s}^{P}$) remained low for most samples. A notable exception was ZnO at 0.5 wt.%, which exhibited the highest polar component (7.39 ± 1.02 mN m^−1^), followed by Cloisite^®^ 30B at 0.1 wt.% (3.91 ± 1.33 mN m^−1^) and ZnO at 0.1 wt.% (3.73 ± 0.48 mN m^−1^), suggesting an increased contribution of polar surface interactions for these compositions.

### 3.4. Surface Roughness Analysis

**Figure 5:**
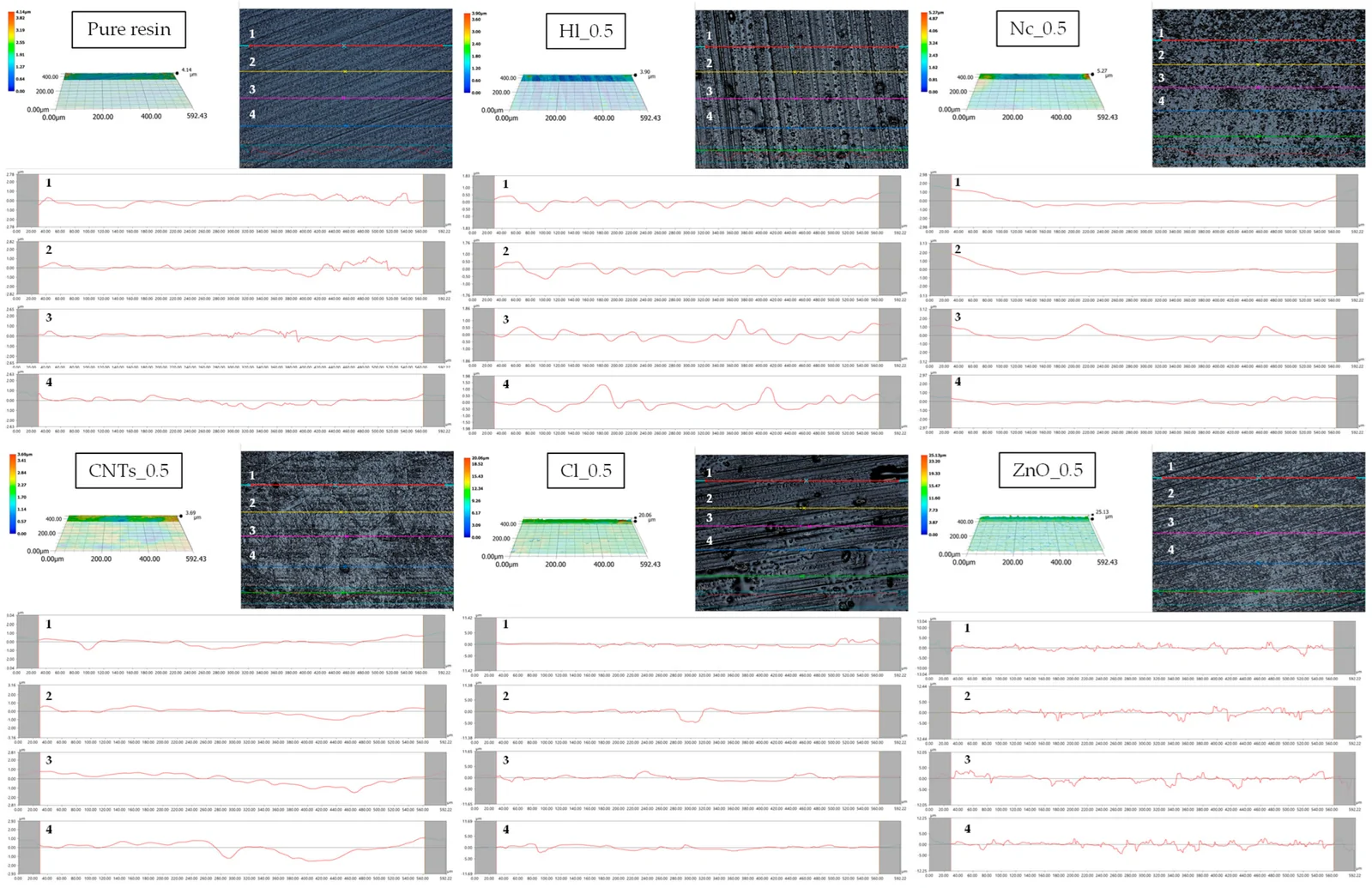
Representative surface topography maps, optical images showing the four measurement traces, and the corresponding roughness profiles of the neat unsaturated polyester resin and representative composites containing 0.5 wt.% halloysite, nanoclay, CNTs, Cloisite^®^ 30B, and ZnO before incubation. Numbers 1–4 indicate the locations of the four roughness measurements on each sample.

Surface roughness (Ra and Rz) values determined before incubation and after exposure to different degradation environments are summarised in Table 3. Representative surface topography maps, optical images with the measurement traces, and the corresponding roughness profiles for the neat resin and representative composites containing 0.5 wt.% nanofillers are presented in Figure 5 (before incubation) and Figures S1–S4 (after incubation in buffer solutions at pH 4, 7, and 9 and after soil burial).

Before incubation, all investigated materials exhibited relatively smooth surfaces with low roughness values. The Ra parameter ranged from 0.26 ± 0.06 to 1.10 ± 0.06 µm, whereas Rz varied between 1.39 ± 0.27 and 20.47 ± 3.74 µm, depending on the nanofiller type and concentration. The representative roughness profiles presented in Figure 5 illustrate the surface morphology of the neat resin and representative composites prior to incubation, showing relatively smooth surfaces with only minor height fluctuations. Incubation in buffer solutions resulted in noticeable changes in surface roughness, the magnitude of which strongly depended on the environmental conditions and composite composition. The most pronounced increase in both Ra and Rz was observed after incubation at pH 4, indicating accelerated surface degradation under acidic conditions. This effect was particularly evident for ZnO- and Cloisite^®^ 30B-containing composites, which exhibited the highest roughness values, reaching Ra = 2.21 ± 0.25 µm and Rz = 22.86 ± 4.25 µm for ZnO_0.1, and Ra = 2.47 ± 0.36 µm and Rz = 13.81 ± 3.00 µm for Cl_0.25. The corresponding roughness profiles shown in Figure S1 reveal the appearance of pronounced peaks and valleys, indicating significant surface degradation.

In contrast, incubation at pH 7 resulted in considerably smaller changes in surface roughness for most materials. Although increases in Ra and Rz were observed for selected composites, the majority of samples exhibited relatively moderate roughness values. The highest values were observed for ZnO_0.25, reaching Ra = 4.09 ± 0.60 µm and Rz = 26.62 ± 3.58 µm, while Cl_0.1 also exhibited an increased Rz value of 9.04 ± 1.40 µm. Exposure to alkaline conditions (pH 9) generally caused intermediate changes in roughness. The most pronounced increases were observed for Cl_0.5 (Ra = 1.44 ± 0.18 µm; Rz = 17.07 ± 4.74 µm) and CNTs_0.5 (Ra = 1.03 ± 0.24 µm; Rz = 6.28 ± 1.74 µm). For the remaining samples, changes were generally more moderate.

Following soil burial, the roughness parameters remained lower than those measured after incubation in the acidic buffer. For most materials, Ra values were below 1 µm and Rz remained below approximately 5 µm, indicating only moderate surface modification during soil exposure. The highest roughness after soil burial was observed for Cl_0.5, reaching Ra = 1.03 ± 0.02 µm and Rz = 7.08 ± 4.21 µm. The representative topography maps and roughness profiles presented in Figure S4 demonstrate that soil burial generally produced less pronounced changes in surface morphology than acidic incubation, which is consistent with the quantitative roughness parameters summarised in Table 3.

### 3.5. Morphological Analysis

**Figure 6:**
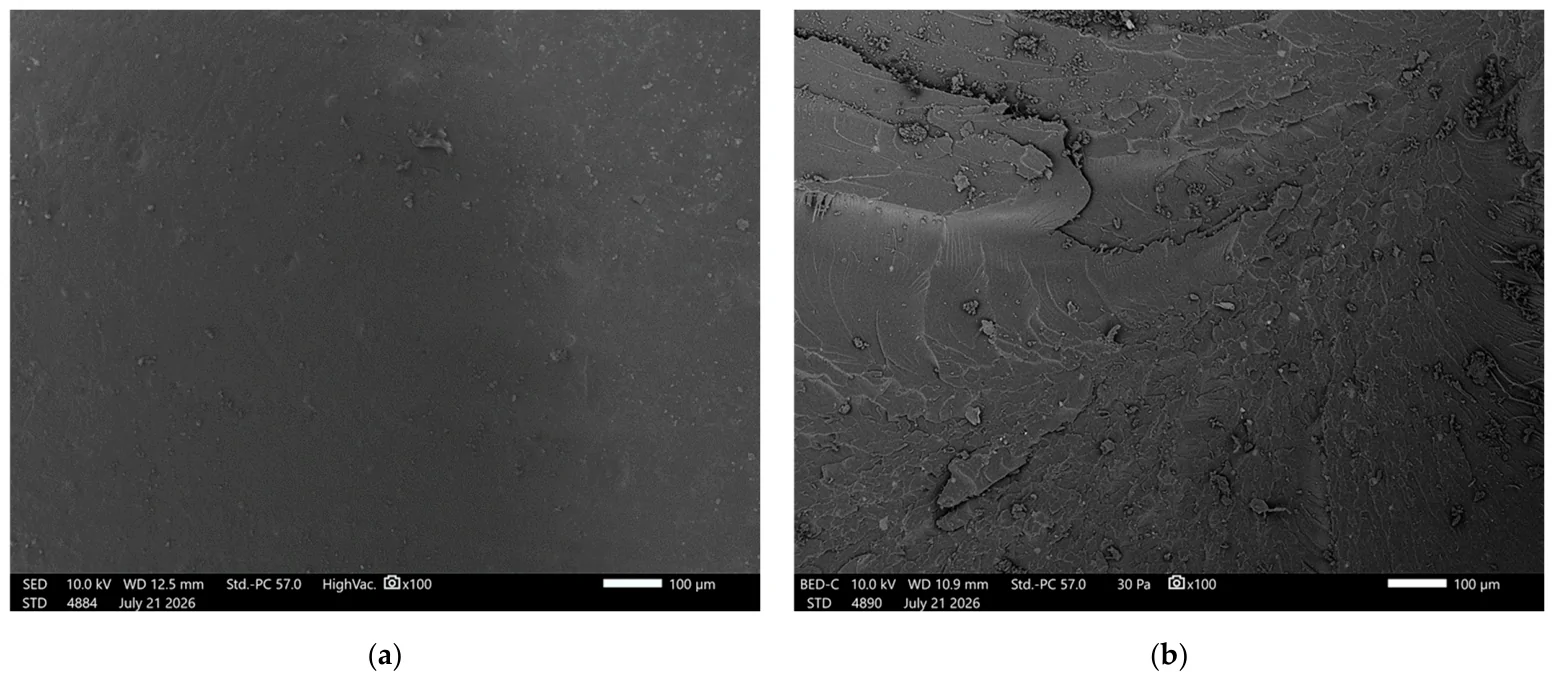

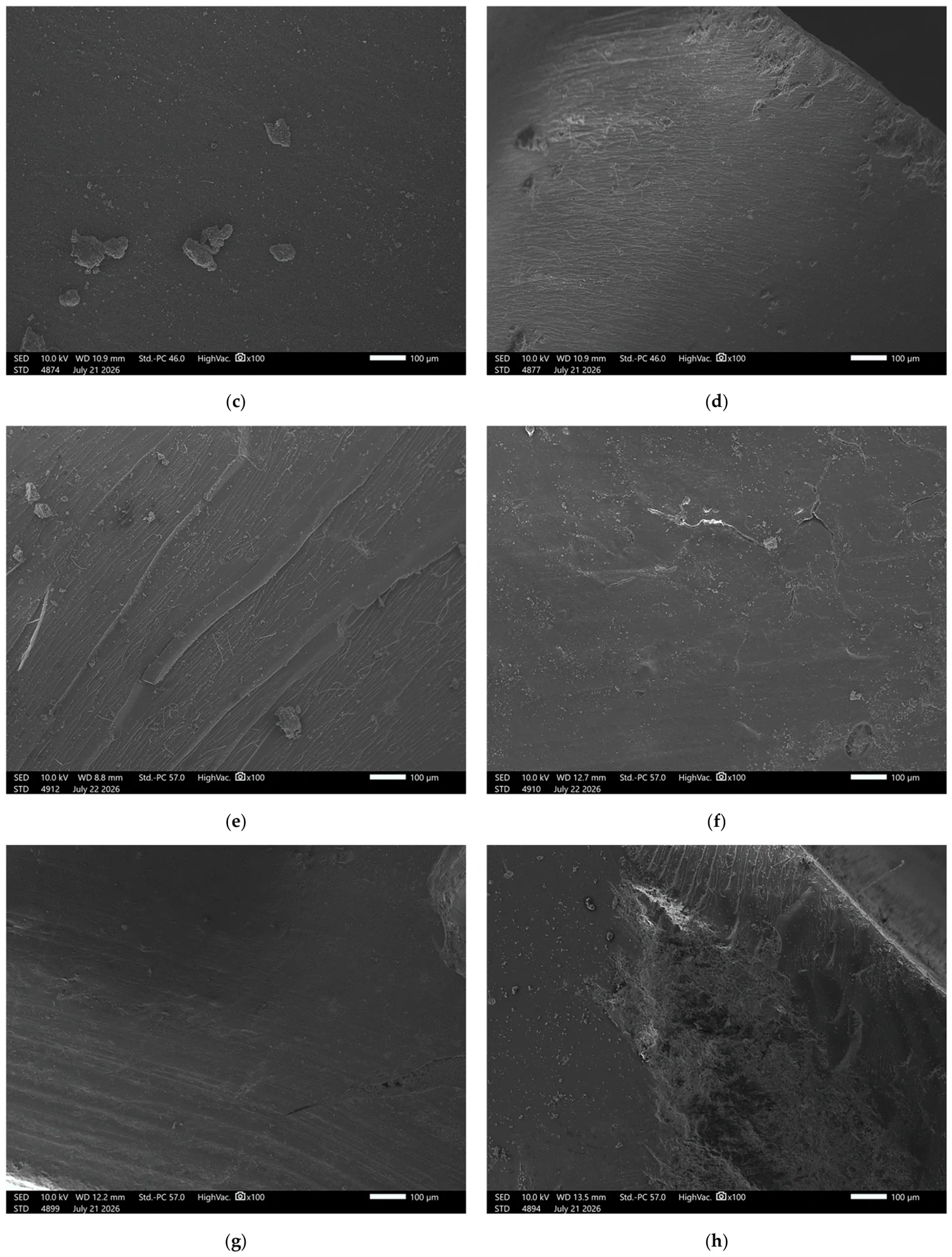

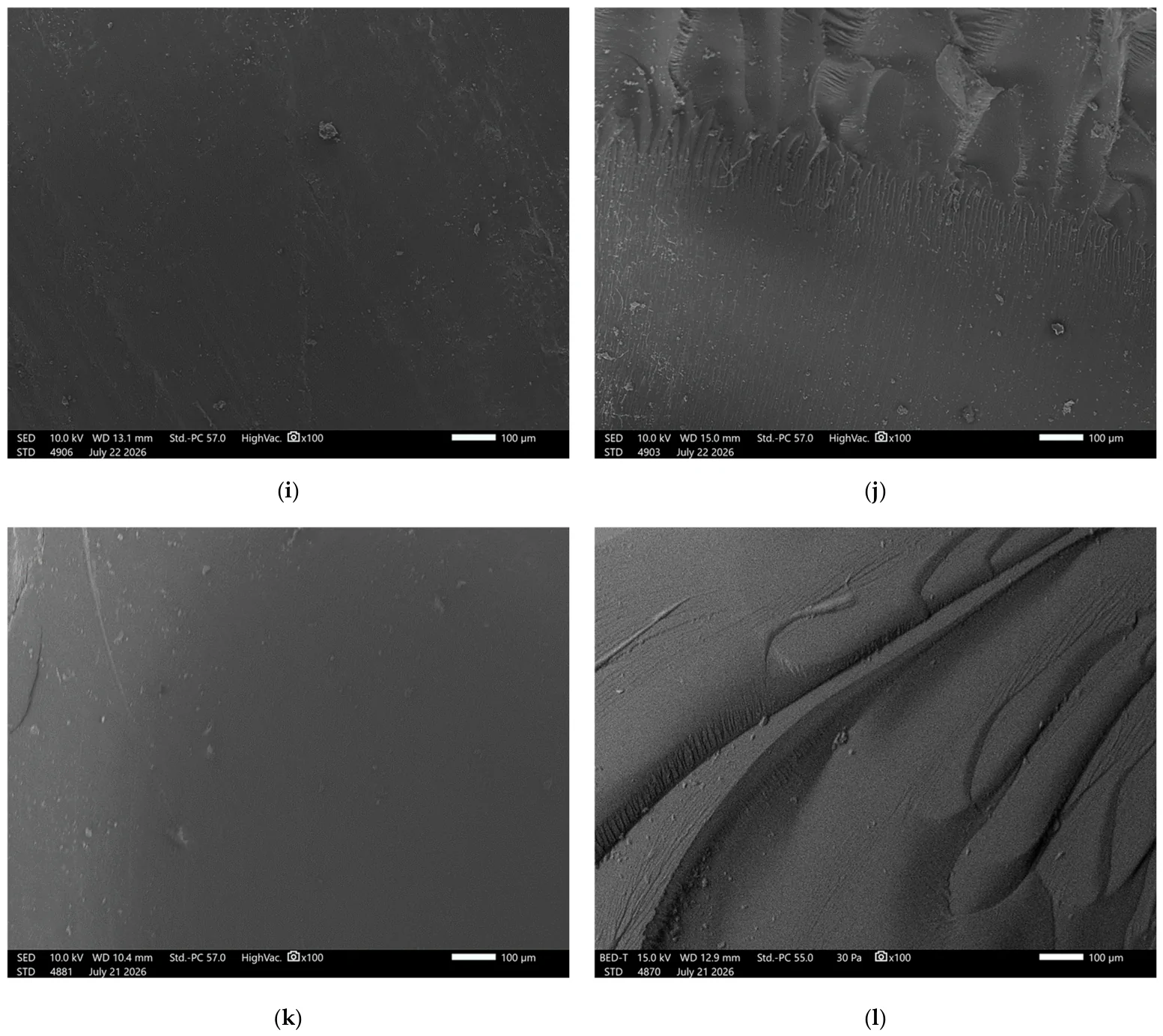
Comparison of the morphology and structure of composites and pure resin. (**a**) Hl_0.5 surface; (**b**) Hl_0.5 fracture; (**c**) Nc_0.5 surface; (**d**) Nc_0.5 fracture; (**e**) CNTs_0.5 surface; (**f**) CNTs_0.5 fracture; (**g**) Cl_0.5 surface; (**h**) Cl_0.5 fracture; (**i**) ZnO_0.5 surface; (**j**) ZnO_0.5 fracture; (**k**) pure resin surface; (**l**) pure resin fracture.

The SEM observations presented in Figure 6 showed that the surfaces of the materials, after the casting and hardening process, were generally relatively smooth, and any visible differences were largely localised. As the surface topography may have largely reflected the mould used during sample preparation, one should be cautious about attributing the observed irregularities directly to the presence of nanofillers. For this reason, fracture images reflecting the manner in which cracks propagate through the material are more reliable for assessing the effect of the modification.

The fracture surface of the pure resin was characterised by a relatively smooth and orderly morphology, with the presence of extensive, flat areas and distinct bands corresponding to the direction of crack propagation. This pattern is typical of brittle fracture in highly crosslinked thermosetting resins, in which the crack propagates rapidly with little plastic deformation. Following the introduction of nanofillers, the fracture morphology changed significantly. More irregular surfaces were observed, along with a greater number of steps, ridges, local depressions and areas of increased roughness. This may indicate that the presence of nanoparticles interrupted the straight-line propagation of the crack and led to its deflection or branching. In such a case, the crack front must follow a more complex path, which is usually associated with greater energy consumption during material failure. In the case of the composite containing haloisite, the fracture was irregular and exhibited local stepped zones. The fracture in the Nc_0.5 was more developed than that in the pure resin, but at the same time relatively homogeneous on a macro-scale. The visible bands and irregularities may indicate a change in the direction of crack propagation due to the presence of nanoclay, whose layered morphology may increase the tortuosity of the crack path. For the CNTs_0.5, a more varied and, in places cracked fracture morphology was observed. This may indicate local disturbances in crack propagation and a change in the stress transfer mechanism within the matrix. The most developed and irregular fracture morphology was observed in the composite containing Cloisite^®^ 30B. The fracture surface featured numerous faults, depressions and areas of varying slope, suggesting significant deflection and dispersion of the crack front. This pattern may indicate more effective interaction between this nanofiller and the polyester matrix. In the case of the ZnO_0.5, the fracture surface exhibited distinct zones with varied topography. Local ridges and bands were visible, which may correspond to changes in the direction of crack propagation. The presence of ZnO may therefore have influenced the local stress field and the mode of damage propagation.

### 3.6. Thermogravimetric Analysis

TGA was carried out to determine the effect of the type and content of nanofillers on the thermal stability of the materials, the temperature at 5% mass loss (T_d,5%_), the rate of degradation and the ability to form a carbon residue. The results are presented in Table 4. The TGA indicates that all the materials underwent a single stage of thermal decomposition, with the maximum rate of mass loss occurring in the range of 391.2–400.7 °C. However, a slight mass loss of up to 1% is observed at approximately 180 °C, which can be attributed to the volatilisation of absorbed moisture, solvent and/or any monomers that did not react. The slight differences in T_d,5%_ indicate that the fillers used did not significantly alter the thermal degradation of the unsaturated polyester resin matrix. This single-stage degradation corresponds mainly to the decomposition of the orthophthalic polyester part of the composites involving styrene-moiety cross-links and the degradation of the polyester backbone [64].

For the pure resin, T_d,5%_ was 272.0 ± 8.3 °C. The introduction of Nc into the orthophthalic polyester matrix resulted in a decrease in T_d,5%_ by 12.1%, 10.9% and 5.0%, for Nc_0.1, Nc_0.25 and Nc_0.5, respectively (Figure S6, Table 4). For the CNT-containing composites, the T_d,5%_ were lower than the control by 9.0%, 8.4% and 14.6% for CNTs_0.1, CNTs_0.25, and CNTs_0.5, respectively. T_d,5%_ in the Closite-containing materials depended on Cl content. T_d,5%_ decreased for: Cl_0.1 and Cl_0.5 by 14.2% and 9.3%, respectively, whilst for Cl_0.25 an increase of 3.9% was recorded (Figure S6, Table 4). For the halloysite-based series, the T_d,5%_ increased by 3.3% for Hl_0.1 and by 4.9% for Hl_0.5, whilst for Hl_0.25 it decreased by 10.0%, compared to pure resin (Figure S5, Table 4). The addition of 0.1% ZnO nanopartices resulted in an increase of 2.5% (ZnO_0.1), whilst for ZnO_0.25 and ZnO_0.5 this parameter decreased by 7.2% and 8.6% respectively (Figure S7, Table 4). The highest T_d,5%_ was obtained for the Hl_0.5 sample: 285.3 ± 11.7 °C, which is 4.9% higher than for the pure resin. This indicates that, at this content, the filler slightly delayed the onset of thermal degradation. The lowest T_d,5%_ was recorded for CNTs_0.5: 232.2 ± 12.0 °C, corresponding to a reduction in T_d,5%_ by14.6%, compared to pure resin. However, a reduction in the temperature of the onset of mass loss does not necessarily imply a reduction in the material’s stability across the entire temperature range. In UPR nanocomposites containing organically modified nanoclays, an earlier onset of mass loss has been reported, associated, amongst other things, with the decomposition of the organic modifier, whilst simultaneously slowing down subsequent degradation and increasing the residue at high temperatures [64]. After heating to 600 °C in a nitrogen atmosphere, the pure resin retained 20.9 ± 7.3% of its mass. The highest residue was obtained for Hl_0.5: 27.6 ± 3.7%, followed by Nc_0.25: 25.1 ± 0.2 percent and ZnO_0.1: 24.4 ± 5.4%. Taking into account the average values for the entire series, the Hl group exhibited the greatest ability to form residue. The lowest final mass was recorded for CNTs_0.25: 8.6 ± 4.0%.

This residue should not be defined as the mass after combustion, as heating in nitrogen leads to pyrolysis rather than combustion of the material. The mass determined at 600 °C therefore comprises the charred organic residue and the inorganic components present in the material. High values in the range of 20–28% are possible in systems exhibiting a capacity for carbonisation. Krishnan et al. reported that polyester resin composites retained approximately 21.5% residual mass at 575 °C under nitrogen, whereas the residue decreased to approximately 13.1% under an air atmosphere [65]. 

In this work, following completion of the thermal decomposition carried out in N2, the samples were subjected to an additional 15 min exposure to air at 600 °C. The change in atmosphere reduced the residue mass but did not lead to complete oxidation. For the pure resin, the mass decreased from 20.9 ± 7.3% to 18.3 ± 7.7%. The highest mass following the short-term oxidation stage was still observed for Hl_0.5 and amounted to 25.8 ± 2.9%. Høgsaa et al. investigated pristine halloysite nanotubes and their counterparts modified with wood-derived biocrude, and found an exceptionally high residual mass of 82.7% for the unmodified halloysite nanotubes even at a temperature of 1000 °C, whilst the modified halloysite nanotubes retained approximately 56–58% of their initial mass. This phenomenon was attributed primarily to the high thermal stability of the inorganic aluminosilicate structure of halloysite, whilst the lower residual mass of the modified samples resulted from the thermal decomposition of the organic fraction of the raw biofuel [66]. High mass residues were also recorded for ZnO_0.1, Nc_0.25 and Hl_0.1: 21.6 ± 6.0%, 20.3 ± 6.3% and 19.6 ± 3.8%, respectively. The lowest residue was found for CNTs_0.25: 5.1 ± 3.6%.

As a residual mass of over 10% was observed for all tested samples, including pure resin, the residues remaining after decomposition at 600 °C cannot be attributed solely to the presence of inorganic fillers. A significant part of these residues most probably corresponds to char formed during the decomposition of the cross-linked polymer matrix. Camacho et al. reported a similar observation, finding a high residue following TGA conducted in N2 for epoxy composites containing only 0.1 wt.% ZnO, approximately 22.8% at 900 °C, whilst a similar value was also obtained for the unmodified epoxy matrix [67]. This indicates that this residue originated primarily from the carbonisation of the polymer matrix, rather than directly from the small amount of ZnO. The lowest mass residues at 600 °C in N2 was found for CNTs_0.25: 5.1 ± 3.6%. For CNTs_0.25, the residual mass at 600 °C under N_2_ was 8.6 ± 4.0%, whereas subsequent oxidative treatment in air at 600 °C for 15 min reduced the residue to 5.1 ± 3.6%, indicating partial oxidation of the carbonaceous char formed during pyrolysis. This observation appears to be supported by the fact that, during continuous heating of UPR-based composites, an additional stage of carbon residue oxidation was observed in the temperature range of approximately 435–566 °C, whilst the complete decomposition of the unmodified resin occurred at temperatures above approximately 575 °C [65]. 

### 3.7. Differential Scaning Calorimetry

DSC analysis was carried out to assess the cross-linking state of the tested materials and determine the effect of nanofiller type and content on their thermal behaviour. Two successive heating cycles were carried out, and the results are presented in Table 5. The first cycle reflected the state of the materials after curing at room temperature and enabled the identification of residual cross-linking. The second cycle, carried out after erasing the thermal history and after any additional cross-linking that may have occurred during the first heating, was used to determine the glass transition temperature (T_g_). 

No residual cross-linking effect was recorded for the pure resin, and its glass transition temperature was 108.9 °C, indicating no measurable post-curing under the DSC conditions applied. However, an exothermic residual cross-linking effect was observed in most nanocomposites, with T_g_: 96.6–123.5 °C and ΔH_res_: 2.189–19.67 J·g^−1^. The highest value of ΔHr_es_ was obtained for CNTs_0.1 (19.67 J·g^−1^), followed by Nc_0.5 (18.55 J·g^−1^) and Nc_0.25 (15.65 J·g^−1^). The smallest residual cross-linking effect was observed for CNTs_0.25 (2.189 J·g^−1^) and ZnO_0.5 (2.632 J·g^−1^), whilst no such effect was detected for ZnO_0.1. T_g_ after the second cycle ranged from 75.9 to 121.6 °C. This indicates that, in some of the nanocomposites, a fraction of groups capable of further reaction remained after the initial curing. Zhang et al. reported similar behaviour for epoxy nanocomposites containing polypyrrole nanofibres. Fully cured pure epoxy resin showed no residual ΔH_res_, and was characterised by Tg = 108.3, whilst the introduction of larger amounts of polypyrrole nanostructures resulted in distinct exothermic effects of residual curing. This was attributed to limited curing and incomplete network formation during the initial cross-linking process. 

In the case of composites with nano-clay, a significant increase was observed as the filler content increased: from 13.66 J·g^−1^ for Nc_0.1, through 15.65 J·g^−1^ for Nc_0.25, to 18.55 J·g^−1^ for Nc_0.5. The values obtained in the second cycle ranged from 105.2 to 111.1 °C and were similar to those obtained for the pure resin. This trend therefore indicates that, as the Nc content increased, the scope of the reactions remaining to be completed during the first heating cycle also increased. Previous studies by Poorabdollah et al. showed that organoclays can alter the curing kinetics of UPR [68]. Furthermore, Danaei et al. demonstrated that Cloisite 10A significantly increased the viscosity of UPR and caused the process to transition earlier into a diffusion-controlled regime. The authors also pointed to a slight reduction in cross-linking density associated with the adsorption of styrene by the nanoclay. In this system, the increase in viscosity was the dominant effect and led to the earlier onset of diffusion-limiting behaviour. For composites containing CNTs, halloysite and Cloisite^®^ 30B, no monotonic relationship was observed, indicating an increase with rising filler content. In the case of CNTs_0.5, the increase in Tg (114.5 °C), compared to the pure resin (5.6 °C), indicates post-curing occurring during the initial heating. At the same time, the relatively low value for CNTs_0.5 (5.720 J·g^−1^) suggests that the additional cross-linking was significantly weaker than for CNTs_0.1 (19.67 J·g^−1^). Carbon nanomaterials can modify the cross-linking process by interacting with reactive components of the system, as described by Monti et al. for unsaturated polyester resins containing carbon nanofibres [69]. Furthermore, for composites containing halloysite, the highest T_g_ value was obtained for Hl_0.5 (114.1 °C). However, Hl_0.5 also exhibited measurable residual cross-linking (6.715 J·g^−1^). The increase may therefore result from both the interaction of halloysite with the matrix and restricted segmental mobility, as well as, in part, from additional cross-linking during the first DSC cycle. Previous studies of UPR/halloysite composites have shown that their thermal behaviour is strongly dependent on filler dispersion, surface modification and interphase interactions [70]. Among the materials containing ZnO, only ZnO_0.1 showed no measurable residual cross-linking effect. At the same time, its T_g_ was 115.7 °C, which was 6.8 °C higher than that of the pure resin. In this case, the increase cannot be attributed to post-curing during the first DSC cycle, as no such effect was recorded. This result therefore suggests a change in the structure/segmental mobility of the matrix associated with the presence of ZnO and/or a more effective initial curing process. For ZnO_0.25 and ZnO_0.5, slight residual cross-linking effects were observed, amounting to 5.121 and 2.632 J·g^−1^ respectively, and their peak temperatures reached the highest values in the entire series: 121.6 and 120.0 °C. In these two cases, additional cross-linking during the first heating cycle may have partly contributed to the increase. This interpretation is consistent with the results of Franco-Urquiza et al. for UPR containing 0.05% wt. ZnO. In pure UPR, the authors observed an effect associated with the restart of the curing process in the range of 90–160 °C, whereas in ZnO nanocomposites this effect did not occur in a similar form. The authors concluded that the presence of ZnO promoted the curing of the resin. They also pointed out that ZnO may restrict the mobility of the polyester segments [71]. The influence of ZnO on cross-linking kinetics was also demonstrated by Ghaffari et al. in an epoxy/polyaminoamide system, where the addition of ZnO resulted in a reduction in the apparent activation energy, and the effect was stronger for nanoparticles than for microparticles, due to the catalytic interaction of ZnO with the reactive groups of the epoxy system [72]. 

### 3.8. Analysis of Gross Heat of Combustion 

An analysis of the PCS was carried out for the pure resin and for the composite Nc_0.1. The Nc_0.1 formulation was selected as a representative nanoclay-modified material containing a low nanofiller concentration to provide a preliminary assessment of whether the incorporation of a small amount of an inorganic phase affects the energetic content of the polyester matrix. The results are presented in Table 6. The neat resin exhibited a PCS value of 29.8395 ± 0.1704 MJ/kg, whereas a value of 28.7640 ± 0.8088 MJ/kg was obtained for Nc_0.1, corresponding to an approximately 3.6% lower mean value. This difference may be associated with the incorporation of the non-combustible inorganic nanoclay fraction into the polymer matrix. However, considering the relatively small difference between the mean values and the greater variability observed for the Nc_0.1 sample, the effect should be interpreted with caution.

The present measurement provides information exclusively on the gross heat of combustion and should not be considered a comprehensive assessment of the flammability or fire behaviour of the investigated composites. Moreover, since only one nanoclay-containing formulation was examined, the obtained results do not allow conclusions to be drawn regarding the influence of nanofiller type or concentration on this parameter. Further systematic investigations involving the complete series of nanocomposites and complementary fire-related measurements would be required to evaluate their overall fire behaviour.

### 3.9. Scratch Resistance Test

**Figure 7:**
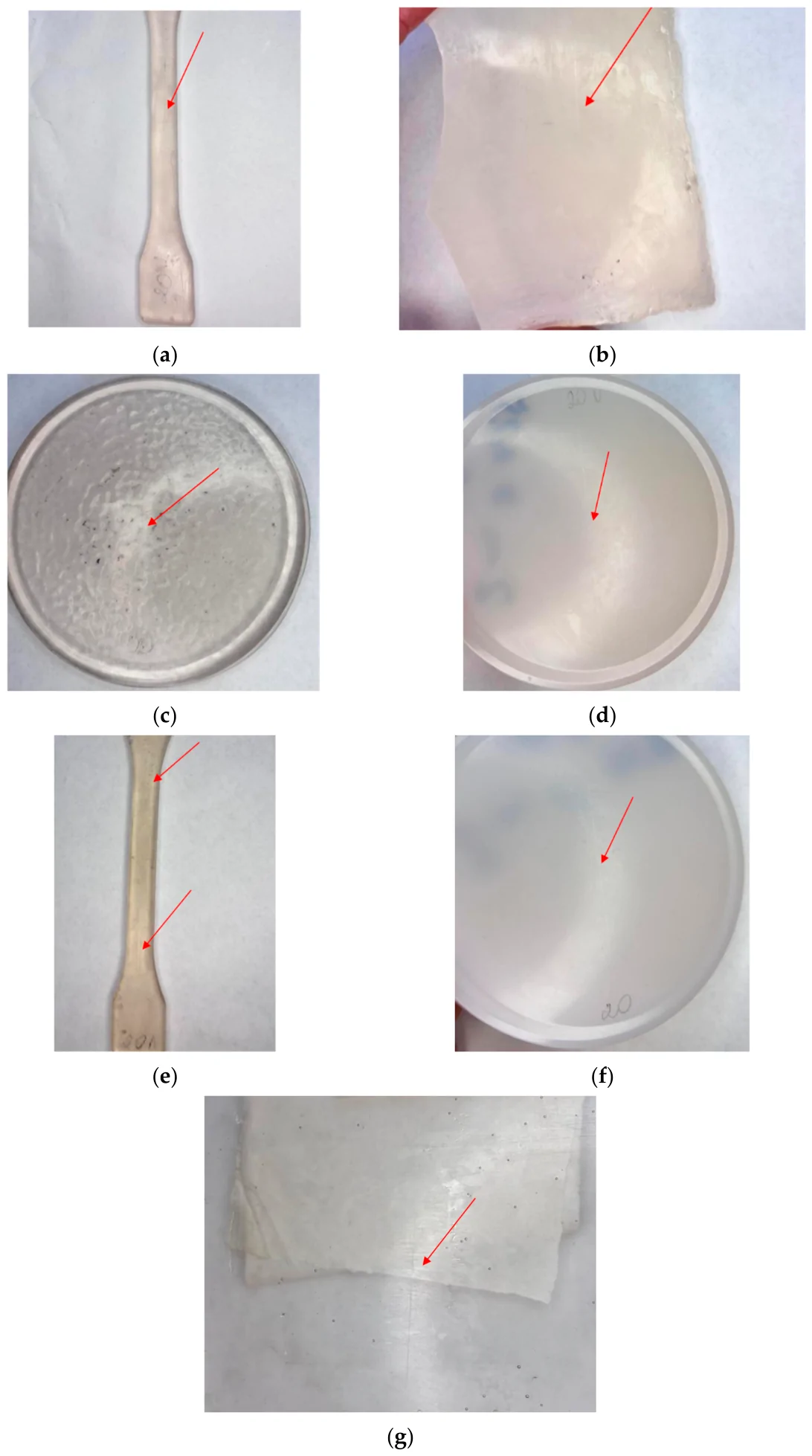
Images demonstrating the scratch resulting from the analysis: (**a**) Nc_0.1; (**b**) ZnO_0.1; (**c**) Nc_0.25; (**d**) ZnO_0.25; (**e**) Nc_0.5; (**f**) ZnO_0.5; (**g**) pure resin. The red arrows indicate the location of the scratch.

Figure 7 presents the results of the presents the results of the qualitative scratch test performed under a constant load. A series of nanoclay and ZnO composites were selected for this study. It was observed that the 20N load applied to the stylus did not produce visually detectable deep penetration into the material but resulted in superficial scratch marks. The resulting scratches are only noticeable when viewed from different angles and under different lighting conditions. A faintly visible surface scratch was observed on all samples; only in the case of the 0.25% nanoclay sample, the scratch was particularly difficult to distinguish visually. However, this may be due to the highly undulating surface of the sample, as it was cast into a mould whose surface was not perfectly smooth. Nevertheless, based on a qualitative visual assessment, the differences in the visibility of the scratches are minimal in all cases. Under the applied test conditions, all investigated materials exhibited only limited visually detectable surface damage.

### 3.10. Pull-Off Strength Measurements

**Figure 8:**
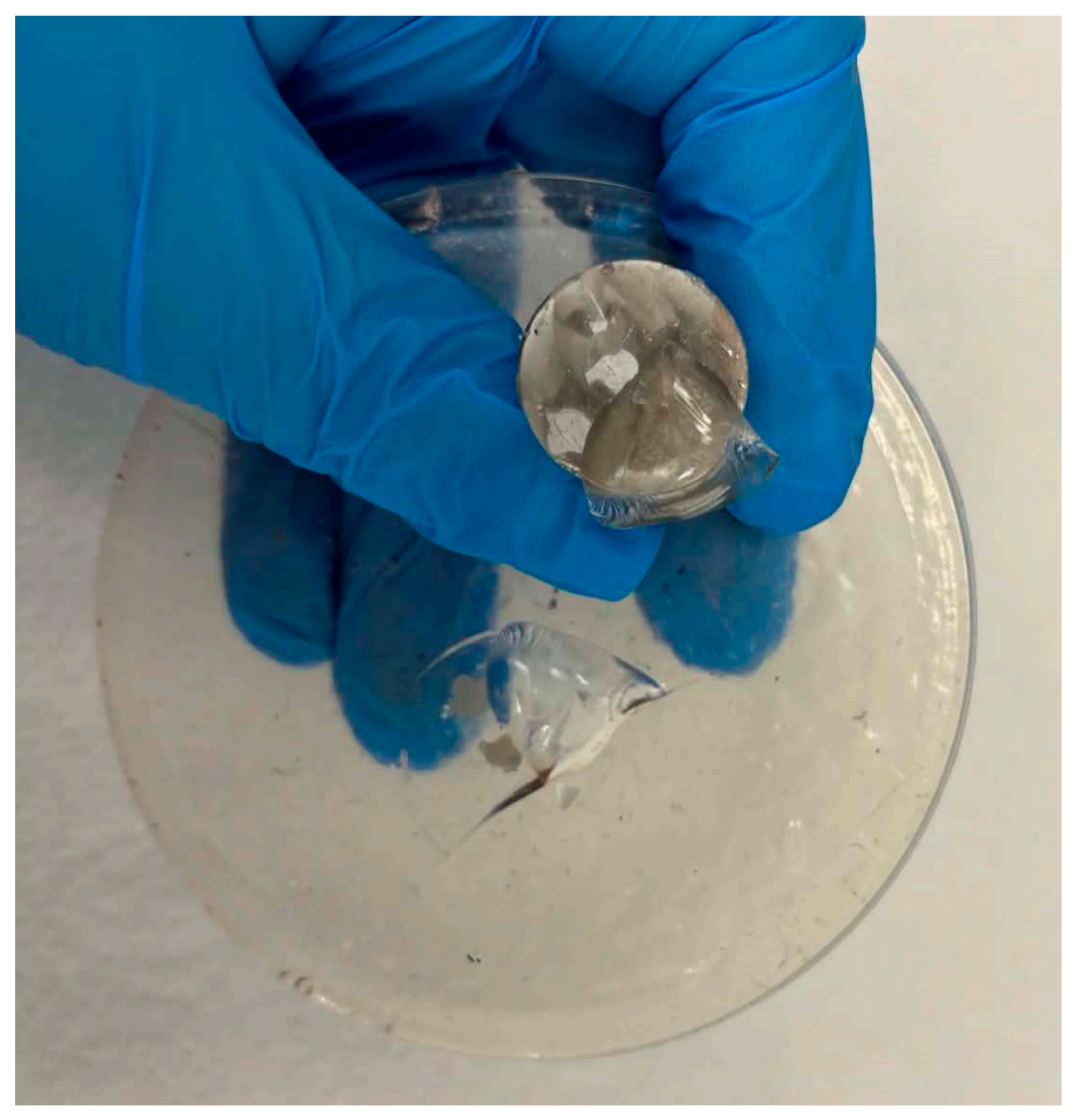
Representative cohesive failure of the Cl_0.5 composite after pull-off testing. The aluminium dolly detached together with a fragment of the composite material.

Pull-off tests demonstrated that both the type and concentration of the nanoadditive used influenced the behaviour of the composites when subjected to a load. The results are presented in Table 7. For the pure resin, an average strength value of approximately 0.63 MPa was obtained, and the failure was of an adhesive nature, during which the mushroom head was observed to detach from the surface of the sample. A similar mechanism was observed for all composites containing 0.1% nanofiller, for which the pull-off values ranged from approximately 0.57 to 0.87 MPa. This indicates that the strength limit of the system was determined by the bond between the adhesive and the composite surface, rather than by the material itself. It was observed that as the nanofiller content increased to 0.25% and 0.5%, the failure mechanism changed for some of the tested samples. In the case of the Hl_0.25, Hl_0.5, Nc_0.25, Nc_0.5, CNTs_0.5, Cl_0.5 and ZnO_0.5 composites, during the pull-off test a fragment of the composite was torn away together with the measuring indenter, leaving a characteristic indentation on the sample surface. This mode of failure indicates a cohesive nature of the material’s fracture and suggests that the strength of the adhesive bond was greater than the local strength of the composite’s near-surface layer. The highest average pull-off stress was recorded for the Cl_0.5 composite (2.73 MPa), for which cohesive failure occurred within the composite. Figure 8 demonstrates an example of the failure mechanism for the Cl_0.5 specimen. A similar failure, involving the detachment of a piece of the composite, was observed in the other samples.

The obtained results suggest that increasing the content of selected nanoadditives may alter the stress transfer behaviour in the near-surface layer of the composite. This suggests that the observed damage may be the result of local inhomogeneities or the potential agglomeration of nanoparticles, or may have been dependent on changes in interfacial properties. The variation in values for adhesive delamination of the dollies from the specimen surface may also suggest a change in surface energy properties due to the presence of the nanofiller.

### 3.11. Compressive Strength

**Figure 9:**
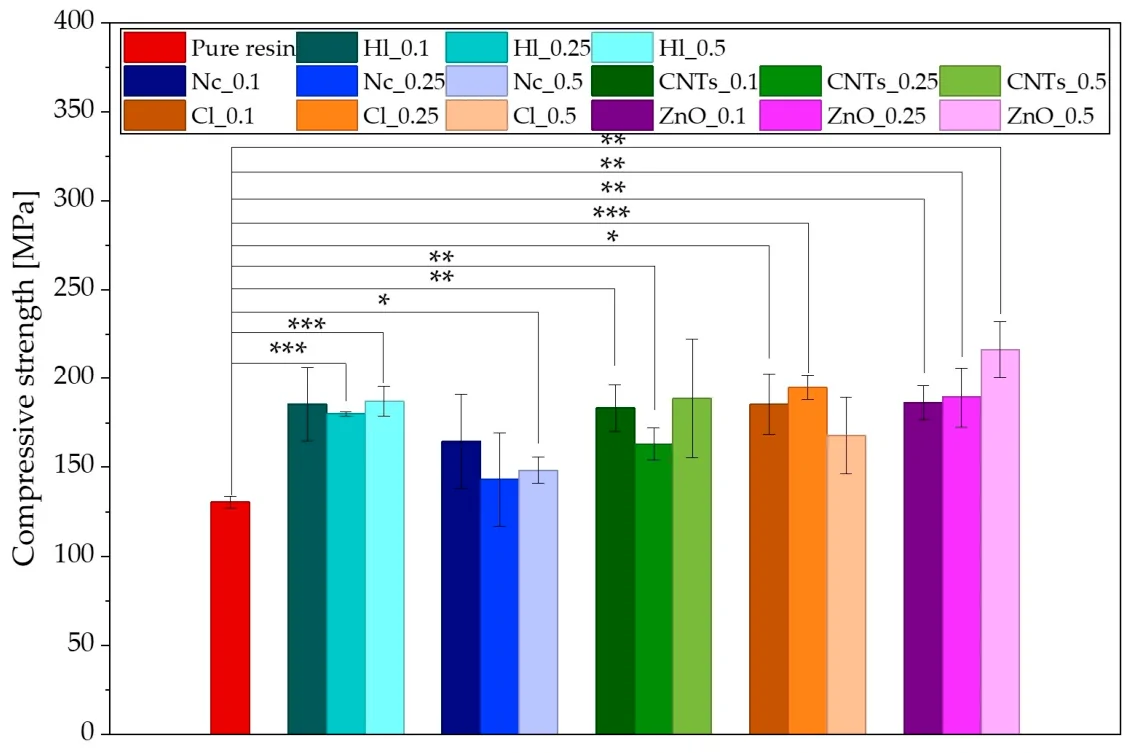
Compressive strength of the neat unsaturated polyester resin (reference) and composites reinforced with halloysite, nanoclay, CNTs, Cloisite^®^ 30B, and ZnO at nanofiller concentrations of 0.1, 0.25, and 0.5 wt.%. Data are presented as mean ± standard deviation (SD). Statistical significance was defined as * *p* < 0.05, ** *p* < 0.01 and *** *p* < 0.001, (*n* = 3).

The results of the compressive strength tests are presented in Figure 9. It was observed that, in all cases, the use of nanoadditives led to an increase in compressive strength compared with the unmodified resin; however, the extent of this effect depended on both the type and concentration of the nanoadditive. The lowest compressive strength value was obtained for the pure resin, at 128 MPa. The highest value, however, was observed for ZnO_0.5, for which the compressive strength exceeded 210 MPa. Very high values were also recorded for Cl_0.25 and Hl_0.5, with compressive strengths of approximately 194 MPa and 186 MPa, respectively. This suggests that both ZnO nanoparticles and layered aluminosilicates effectively reinforced the polyester matrix and limited the development of local stress concentrations during compression. In the case of composites modified with halloysite, a gradual increase in strength was observed as the nanofiller content increased. However, the differences between the individual concentrations were not very large, which may indicate that even a small amount of halloysite was sufficient to achieve a reinforcing effect. A slightly different trend was observed for composites containing nanoclay. The highest strength was achieved for Nc_0.1, whilst a further increase in the nanofiller content led to a decrease in this parameter. This behaviour may indicate a deterioration in the homogeneity of the material’s structure at higher nanoclay concentrations. A similar trend was observed for composites modified with CNTs. The highest strength was achieved for CNTs_0.25, whilst a reduction in strength was observed at a concentration of 0.5%. This phenomenon is frequently described in the literature and is associated with poorer dispersion of nanotubes at higher concentrations. The formation of potenatial local agglomerates may limit the effective stress transfer between the matrix and the nanofiller and lead to the formation of microvoids that act as crack initiation sites. Interesting results were also obtained for composites containing Cloisite^®^ 30B. The highest strength was recorded for Cl_0.25, whilst a further increase in content to 0.5% resulted in a clear decrease in strength. This result suggests the existence of an optimal concentration of the nanofiller at which the most effective interaction between the polyester matrix and the nanofiller is achieved. Composites containing ZnO exhibited different behaviour. In this case, the strength remained at a similar level for ZnO_0.1 and ZnO_0.25, whilst for ZnO_0.5 a marked increase was observed, reaching the highest value among all the materials tested. Despite the presence of local morphological heterogeneities visible in the SEM observations, including regions that may correspond to ZnO agglomeration, the ZnO_0.5 composite exhibited the highest compressive strength. This indicates that such local heterogeneity did not prevent mechanical reinforcement under the applied compression conditions. However, because filler dispersion was not quantified, the relationship between ZnO distribution and mechanical performance cannot be established unequivocally. 

**Figure 10:**
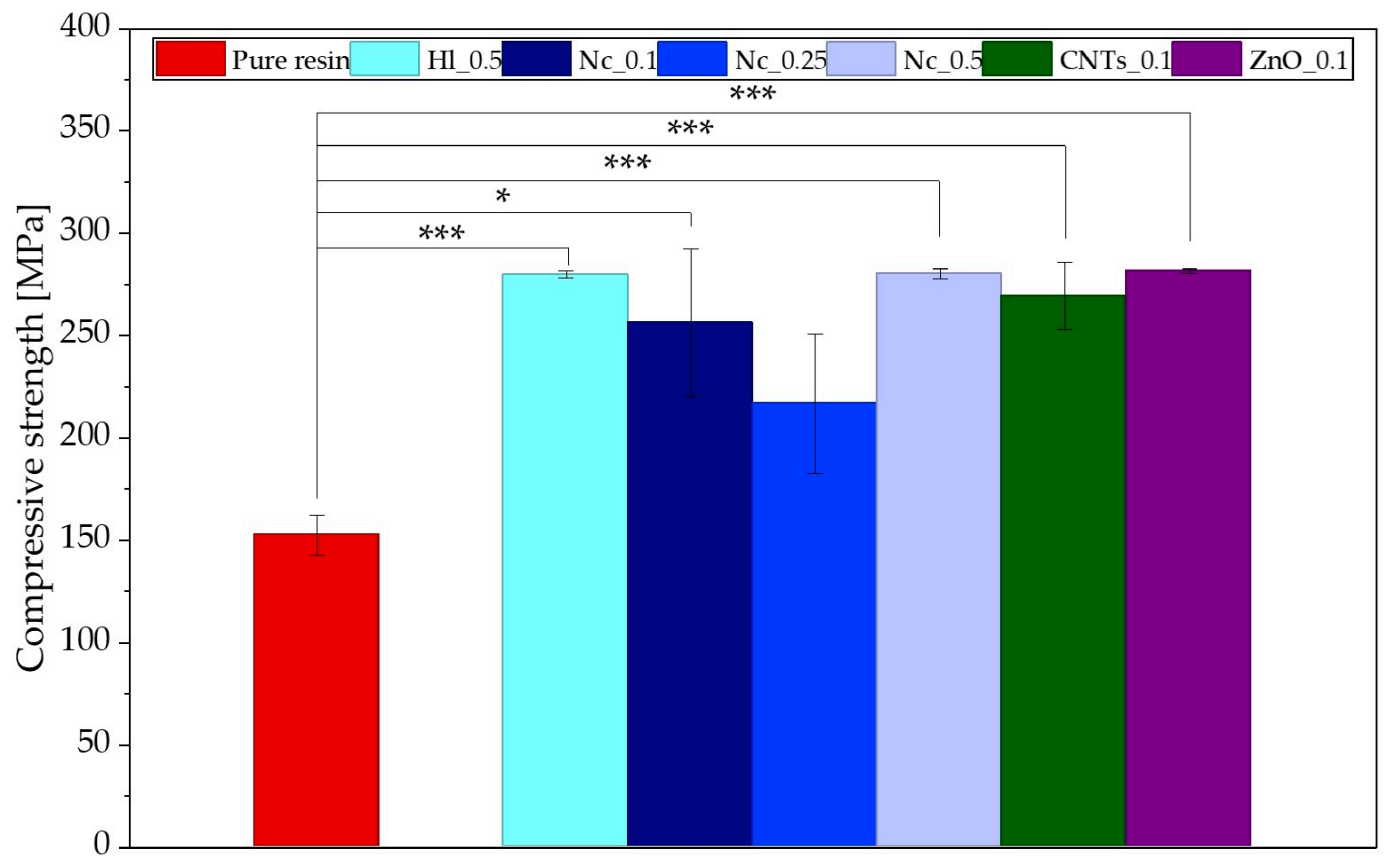
Compressive strength of the neat unsaturated polyester resin (reference) and composites reinforced with halloysite, nanoclay, CNTs, Cloisite^®^ 30B, and ZnO at nanofiller concentrations of 0.1, 0.25, and 0.5 wt.% following exposure to ageing tests. Data are presented as mean ± standard deviation (SD). Statistical significance was defined as * *p* < 0.05, ** *p* < 0.01 and *** *p* < 0.001, (*n* = 3).

Figure 10 presents the results of compressive strength tests on the composites following accelerated atmospheric ageing. After the ageing process, all the tested composites showed a compressive strength higher than that of the pure polyester resin. For the unmodified resin, the average compressive strength was 152.5 ± 9.7 MPa, whilst all composites achieved values exceeding 200 MPa, which demonstrates the beneficial effect of the nanoadditives used on the retention of mechanical properties following exposure to atmospheric conditions. The highest strength values were obtained for ZnO_0.1, Nc_0.5, and Hl_0.5; these were 281.6 ± 1.1 MPa, 280.2 ± 2.6 MPa and 279.9 ± 1.8 MPa, respectively. These materials were also characterised by very small standard deviations, indicating the homogeneity of their mechanical properties following the ageing process. In the case of composites containing nanoclay, a dependence on the concentration of the nanoadditive was observed. The lowest strength was obtained for the sample containing 0.25 wt.% nanoclay (216.7 ± 34.1 MPa), whilst increasing the content to 0.5 wt.% resulted in a significant increase in strength to 280.2 MPa. CNTs_0.1 achieved a strength of 269.3 ± 16.2 MPa, which also significantly exceeded the value obtained for the pure resin. The results suggest that the incorporated nanoadditives limited the negative impact of accelerated atmospheric ageing on the mechanical properties of the composites. It should be noted, however, that the ageing process was carried out at 60 °C during UV exposure and at 50 °C during the condensation stage, which may have promoted further cross-linking of the polyester resin (post-curing). Consequently, the observed mechanical properties result from the combined effect of degradation processes induced by UV radiation and moisture, as well as the continued maturation of the polymer network.

### 3.12. Tribological Testing

**Figure 11:**
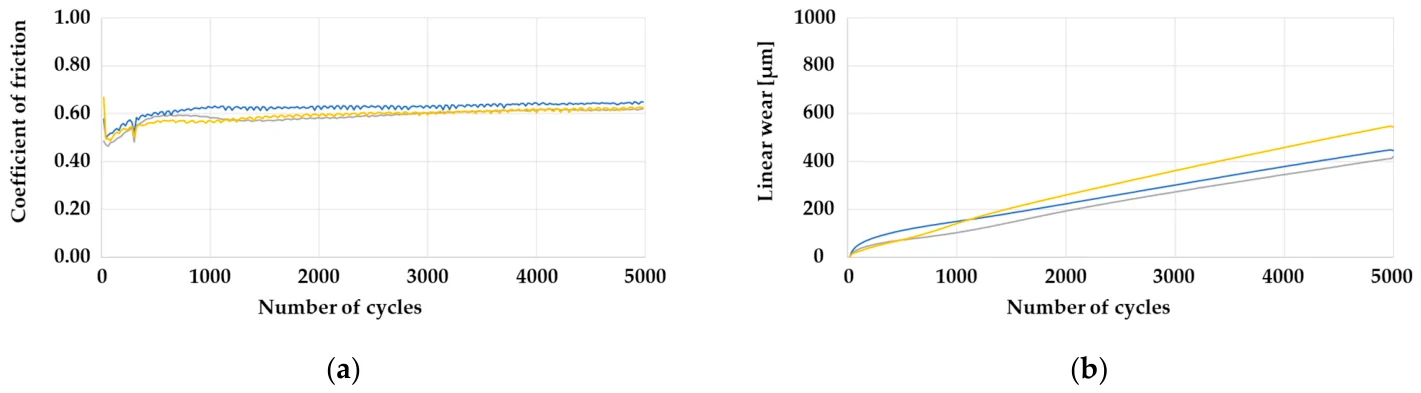

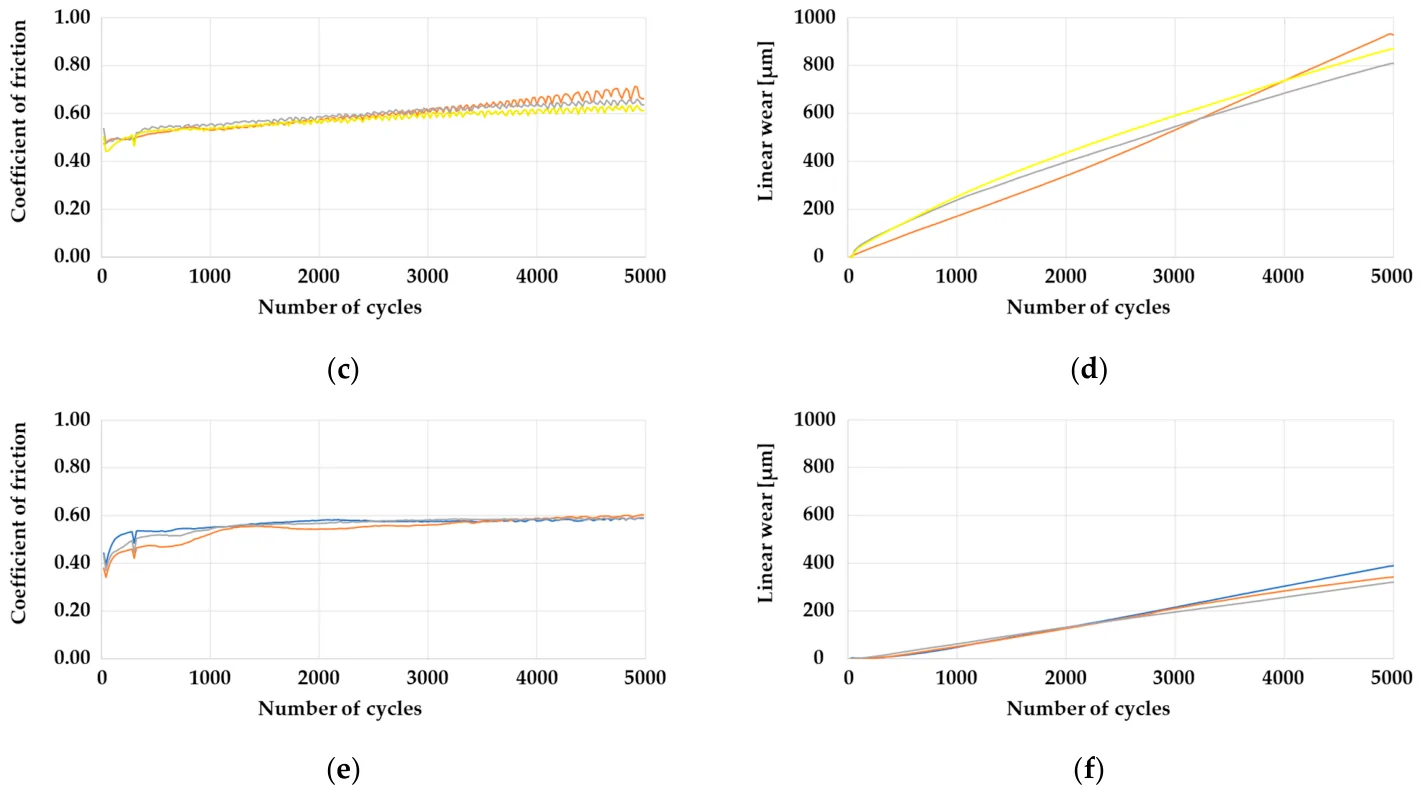
Evolution of the CoF as a function of the number of cycles for (**a**) pure resin; (**c**) Nc_0.1; (**e**) ZnO_0.1; and linear wear as a function of the number of cycles for (**b**) pure resin; (**d**) Cl_0.1; (**f**) ZnO_0.1. In each panel, the three differently colored curves represent separate measurements (*n* = 3).

Figure 11 presents the trends in the coefficient of friction (CoF) and linear wear as a function of the number of cycles for the pure polyester resin and Nc_0.1 and ZnO_0.1, whilst Table 8 summarises the parameters of average CoF, linear wear, and mass loss of the plate and pin. All the materials tested exhibited similar friction behaviour, which can be divided into two distinct stages. In the initial phase, a rapid increase in CoF was observed, corresponding to the running-in stage of the mating surfaces. During this period, local surface irregularities are removed, and the effective contact area between the specimen and the pin increases. 

For the pure polyester resin, the average CoF was 0.602 ± 0.018. The curves were relatively stable, and the differences between individual repetitions were minor. At the same time, linear wear increased linearly with the number of cycles, reaching a value of 439 ± 82,261 µm at the end of the test. This suggests a uniform wear process and the absence of sudden changes in the friction mechanism during the course of the tests. For Nc_0.1, a reduction in the average CoF to 0.587 ± 0.012 was observed, suggesting a reduction in the resistance to motion between the interacting surfaces. However, analysis of the curves indicates that, after approximately 3000 cycles, more pronounced fluctuations in the CoF appear, which were not observed for the pure resin or ZnO_0.1. This may indicate a change in the wear mechanism during the final phase of the test, potentially associated with the progressive detachment of wear debris or localised spalling of the composite surface. The highest mass loss of 0.251 ± 0.016 g was also observed for this composite. The most favourable tribological properties were obtained for ZnO_0.1, which exhibited the lowest average CoF of 0.558 ± 0.010, the lowest linear wear of 372 ± 44,405 µm and the lowest mass loss of 0.107 ± 0.010 g. The stability of the CoF curves indicates that the wear process proceeded uniformly. Furthermore, the linear wear curve remained almost linear throughout the entire duration of the test. This suggests that the presence of ZnO promotes the stabilisation of tribological contact and limits the rate of degradation of the composite surface. 

**Figure 12:**
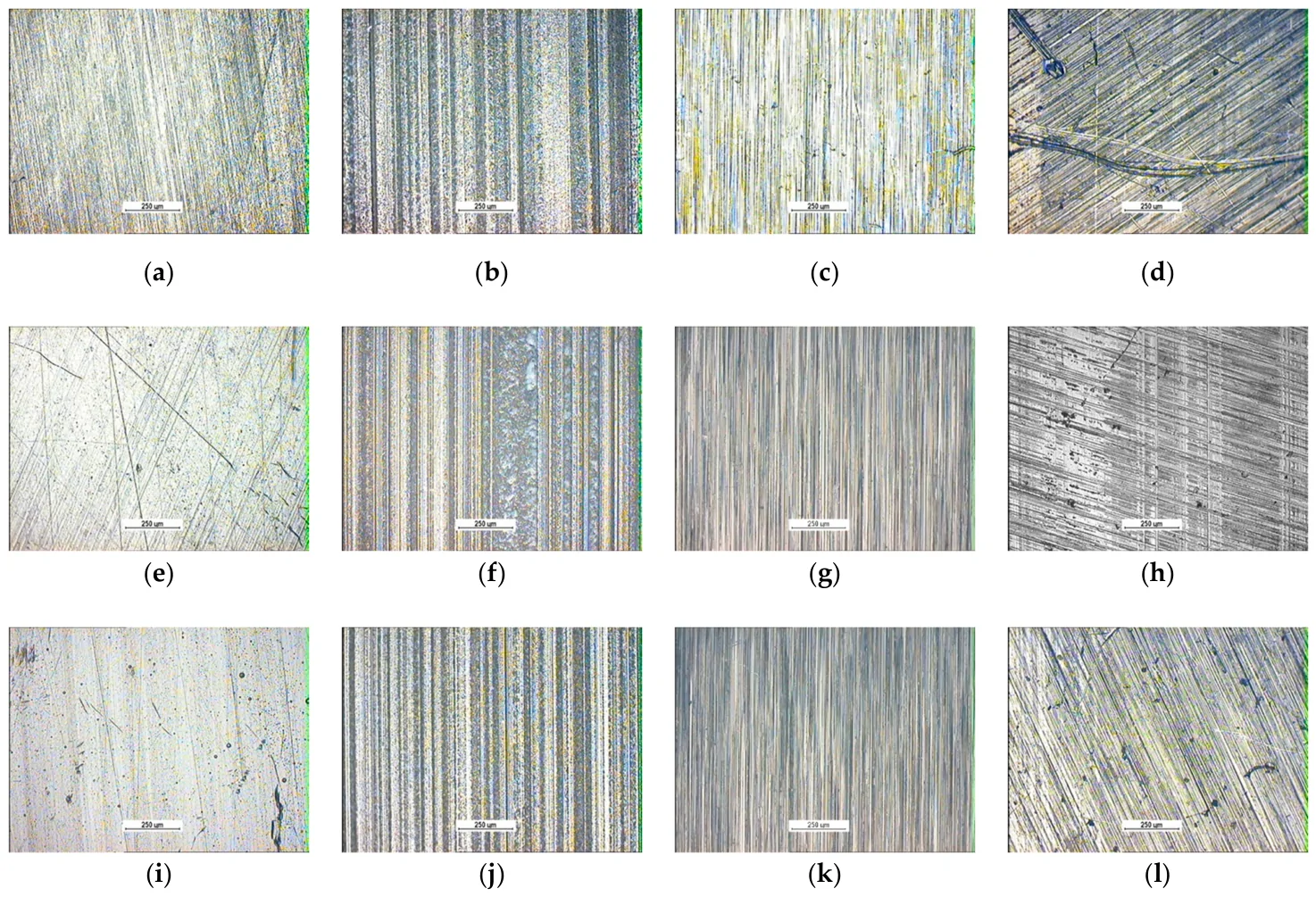
Microscopic images of the sample surfaces before and after tribological testing. Pure resin (**a**) sample before; (**b**) sample after; (**c**) pin before; (**d**) pin after. Nc_0.1 (**e**) sample before; (**f**) sample after; (**g**) pin before; (**h**) pin after. ZnO_0.1 (**i**) sample before; (**j**) sample after; (**k**) pin before; (**l**) pin after.

Figure 12 presents microscopic images of the surfaces of the samples and steel pins before and after the tribological tests. For all the materials analysed, a relatively homogeneous surface was observed prior to the start of the test, on which only traces of mechanical machining and minor local irregularities were visible. Following the tribological tests, characteristic wear marks appeared on the surface of all samples in the form of parallel scratches aligned with the direction of the pin movement, indicating that the abrasive wear mechanism was predominant.

**Figure 13:**
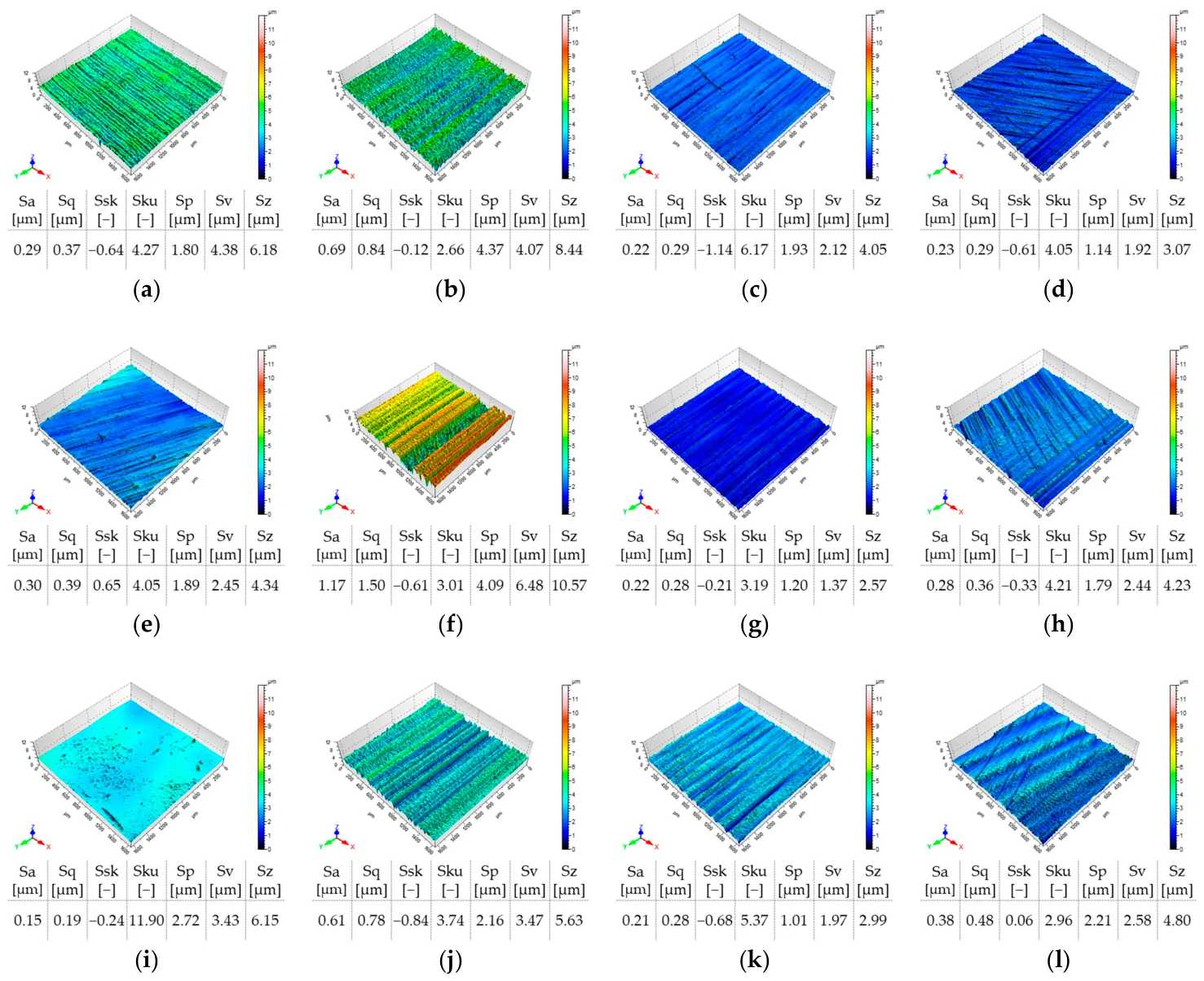
Geometric structure and 3D surface topography parameters of the samples before and after tribological testing. Pure resin (**a**) sample before; (**b**) sample after; (**c**) pin before; (**d**) pin after. Nc_0.1 (**e**) sample before; (**f**) sample after; (**g**) pin before; (**h**) pin after. ZnO_0.1 (**i**) sample before; (**j**) sample after; (**k**) pin before; (**l**) pin after.

The 3D surface topography and the corresponding geometric parameters before and after the tribological tests are presented in Figure 13. For all the materials tested, an increase in the parameters describing surface roughness was observed after the tribological tests, particularly in the arithmetic mean height (Sa) and the root mean square height (Sq). This indicates an increase in surface roughness due to localised damage to the material caused by the wear process. The greatest changes were recorded for Nc_0.1. The Sa value increased from 0.30 µm to 1.17 µm, whilst Sq increased from 0.39 µm to 1.50 µm. At the same time, the maximum surface height (Sz) reached 10.57 µm, the highest value among the analysed samples following the test. The results obtained indicate a significant development of the surface topography and the formation of deep grooves and local irregularities, which is consistent with the highest linear wear observed during the tribological tests. In the case of the pure resin, an increase in roughness parameters was also observed; however, the changes were markedly smaller than for Nc_0.1.

### 3.13. Short-Term Accelerated Abiotic Weathering Test

**Figure 14:**
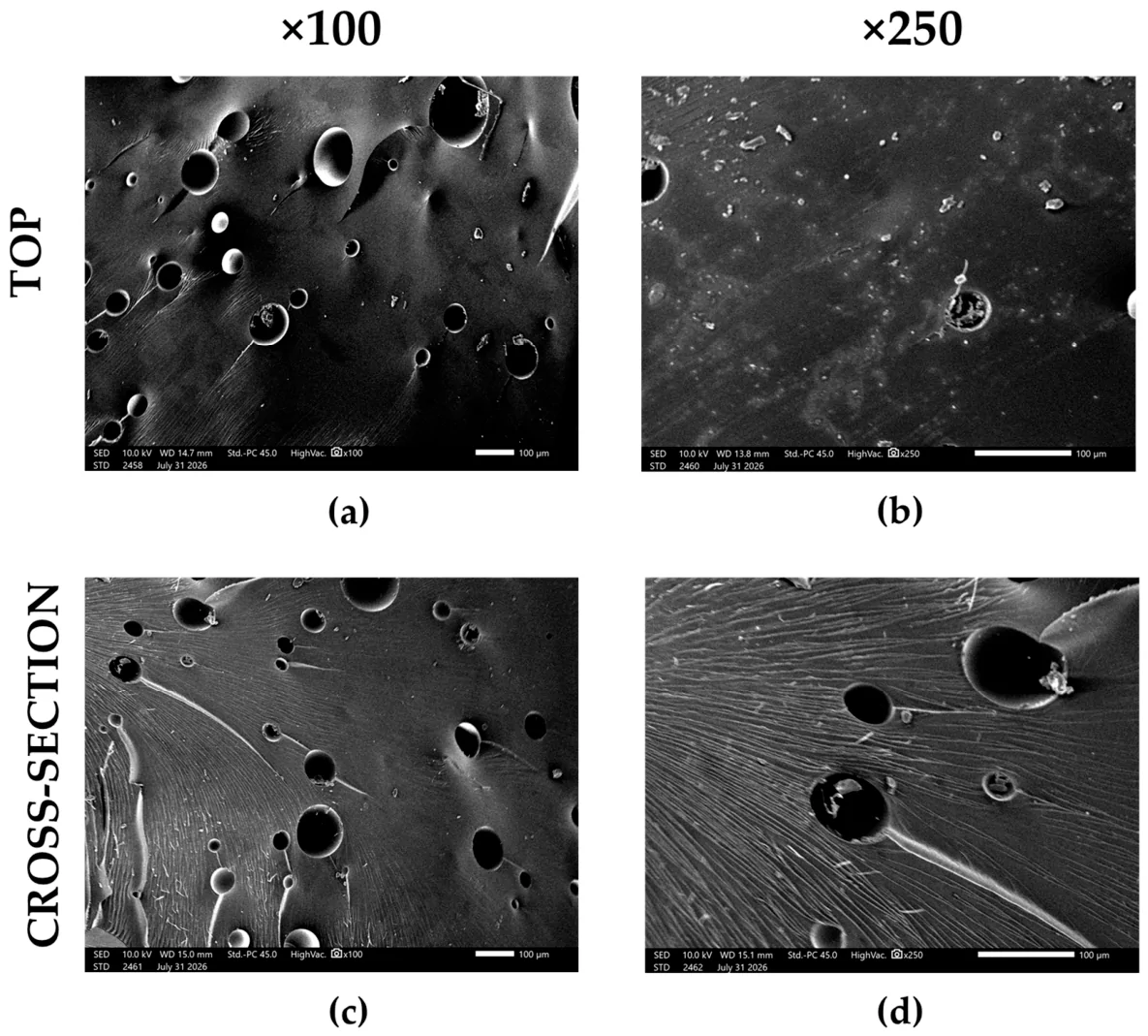
Representative SEM micrographs of the top surfaces and cross-sectional fracture surfaces of the pure resin following 100 h of exposure to accelerated abiotic ageing conditions. (**a**) top, magnifications 100×; (**b**) top, magnifications 250×; (**c**) cross-section, magnifications 100×; (**d**) cross-section, magnifications 250×. Scale bars: 100 µm.

**Figure 15:**
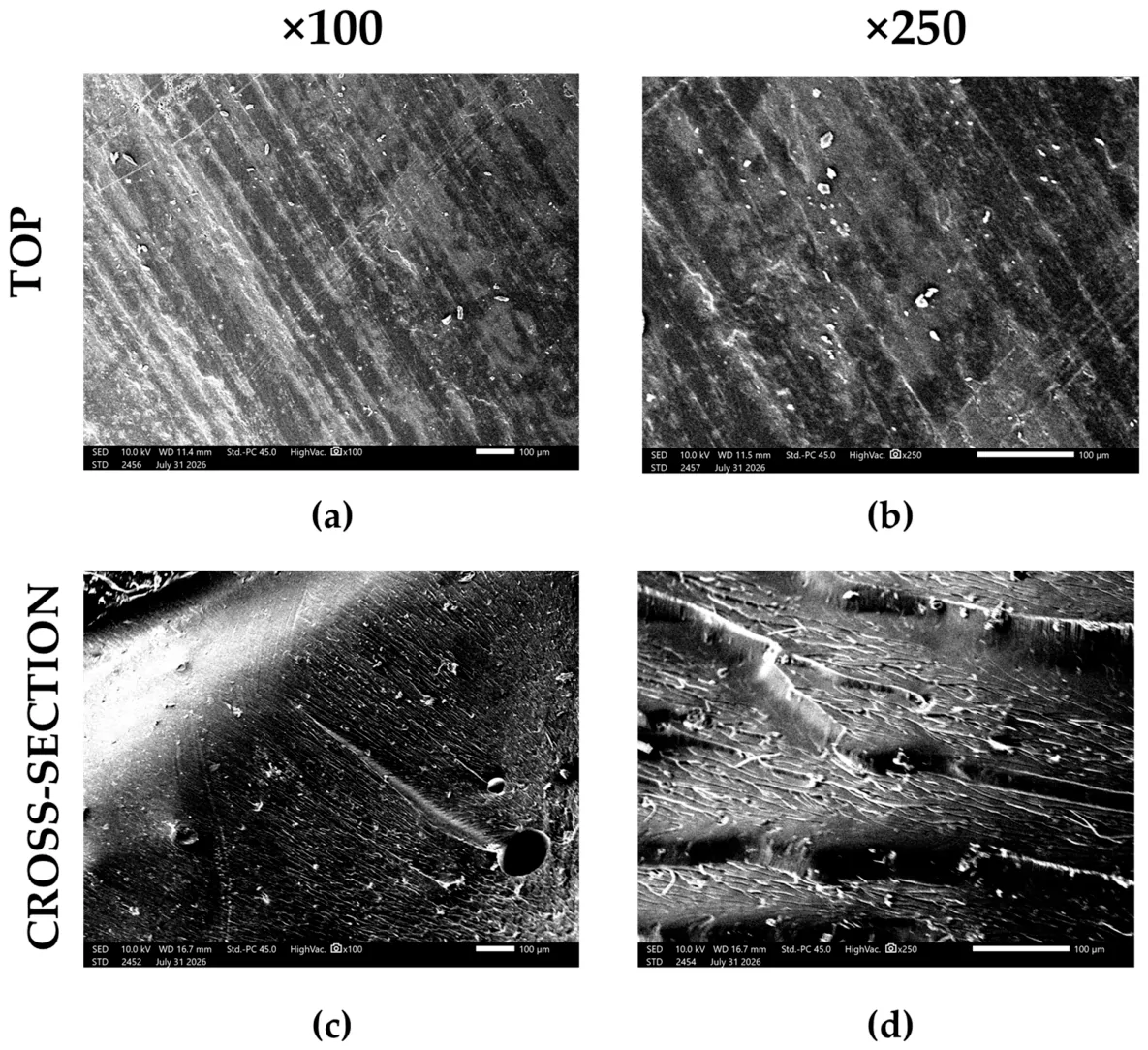
Representative SEM micrographs of the top surfaces and cross-sectional fracture surfaces of Nc_0.5 nanocomposite following 100 h of exposure to accelerated abiotic ageing conditions, (**a**) top, magnifications 100×; (**b**) top, magnifications 250×; (**c**) cross-section, magnifications 100×; (**d**) cross-section, magnifications 250×. Scale bars: 100 µm.

**Figure 16:**
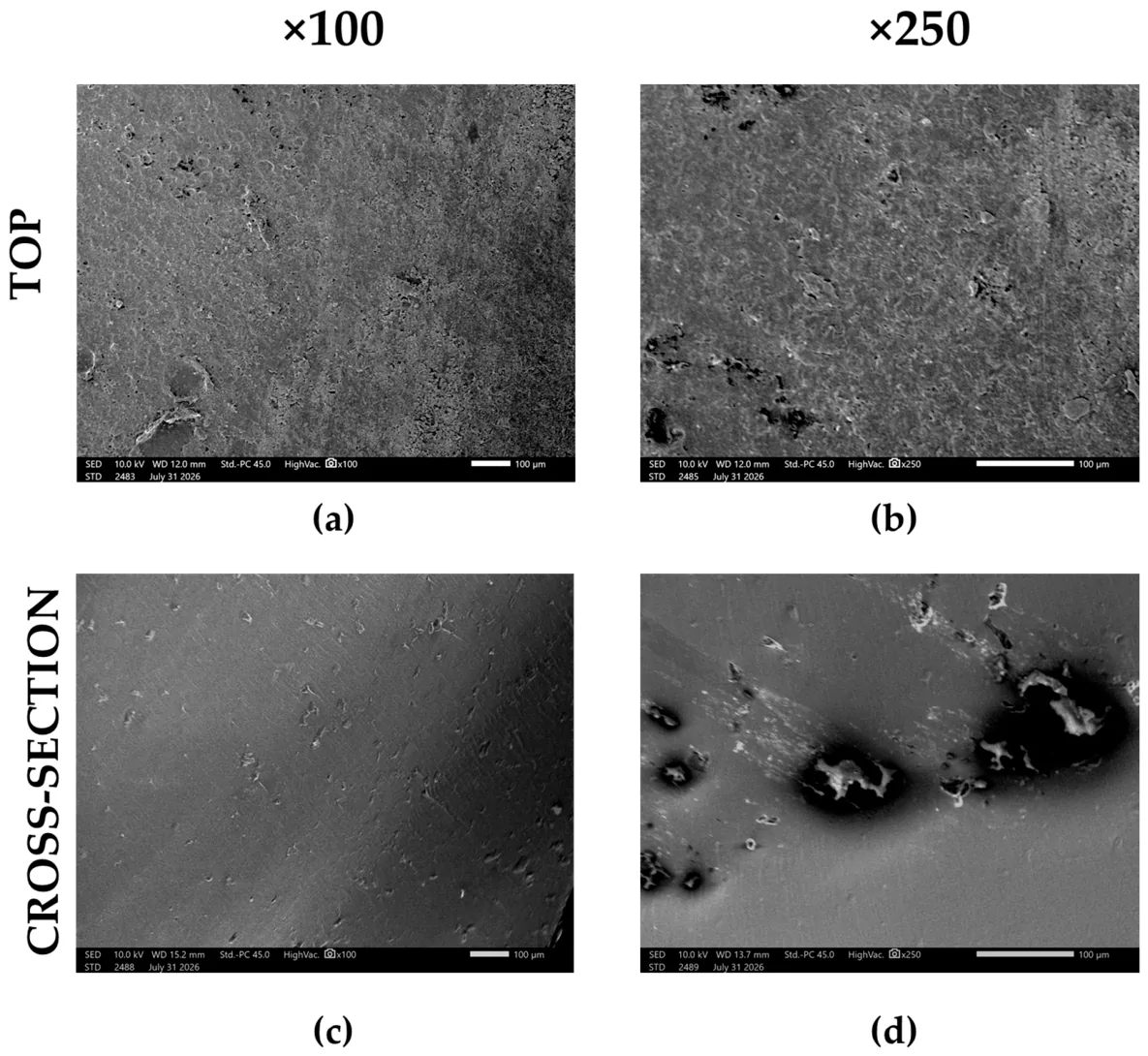
Representative SEM micrographs of the top surfaces and cross-sectional fracture surfaces of Hl_0.5 nanocomposite following 100 h of exposure to accelerated abiotic ageing conditions. (**a**) top, magnifications 100×; (**b**) top, magnifications 250×; (**c**) cross-section, magnifications 100×; (**d**) cross-section, magnifications 250×. Scale bars: 100 µm.

SEM observations of the top surfaces after 100 h of abiotic ageing revealed pronounced changes in the morphology of the investigated materials. The neat resin exhibited the most extensive surface degradation, characterised by numerous microcracks and large, rounded surface defects (Figure 14). Partial deterioration of the polymer matrix also resulted in the exposure of nanofiller particles at the composite surfaces. This effect was particularly evident for CNTs_0.1, ZnO_0.1, Nc_0.25, and Nc_0.5 (Figures S11 and S12). In the Nc-containing composites, the amount of exposed filler increased with increasing nanofiller content, indicating progressive removal or erosion of the surrounding polymer matrix (Figure 15). In contrast, only a limited amount of exposed filler was observed on the surface of the H1_0.5 sample (Figure 16). Overall, the top-view SEM images indicate that abiotic ageing induced matrix degradation, microcrack formation, and local exposure of the dispersed nanofillers, with the extent of these changes depending on the type and concentration of the incorporated filler.

Cross-sectional SEM micrographs provided additional information on the internal morphology of the materials, revealing the direction and extent of damage propagation as well as the presence and distribution of the incorporated nanoparticles within the composite matrix. In the neat resin, rounded and cavity-like microcracks were also observed in the cross-section, indicating that the degradation-induced damage was not restricted to the outer surface but extended into the bulk of the material (Figure 14, Figures S11 and S12).

**Figure 17:**
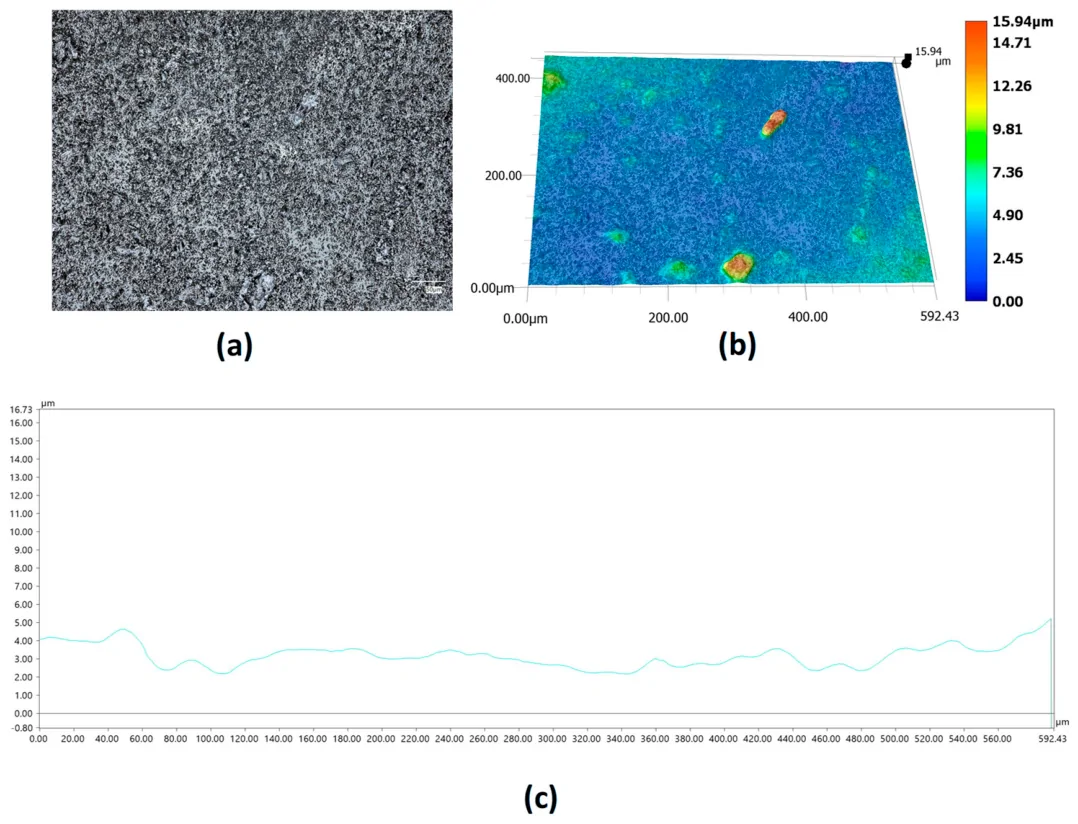
Surface morphology and topography of pure resin after accelerated abiotic weathering test: (**a**) two-dimensional optical micrograph recorded at 500× magnification; (**b**) three-dimensional surface topography with a colour-coded height scale; (**c**) representative line profile recorded across the analysed surface.

**Figure 18:**
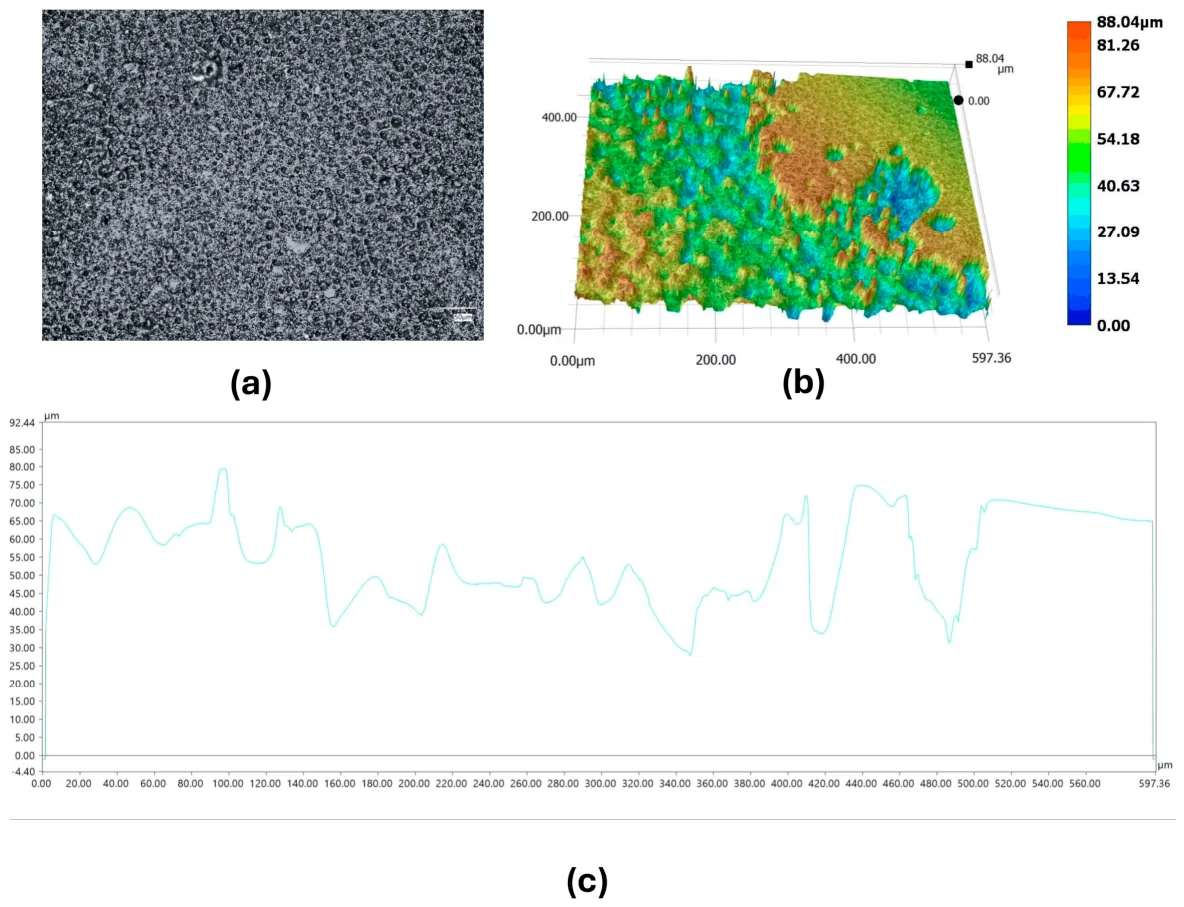
Surface morphology and topography of Nc_0.5 after accelerated abiotic weathering test: (**a**) two-dimensional optical micrograph recorded at 500× magnification; (**b**) three-dimensional surface topography with a colour-coded height scale; (**c**) representative line profile recorded across the analysed surface.

**Figure 19:**
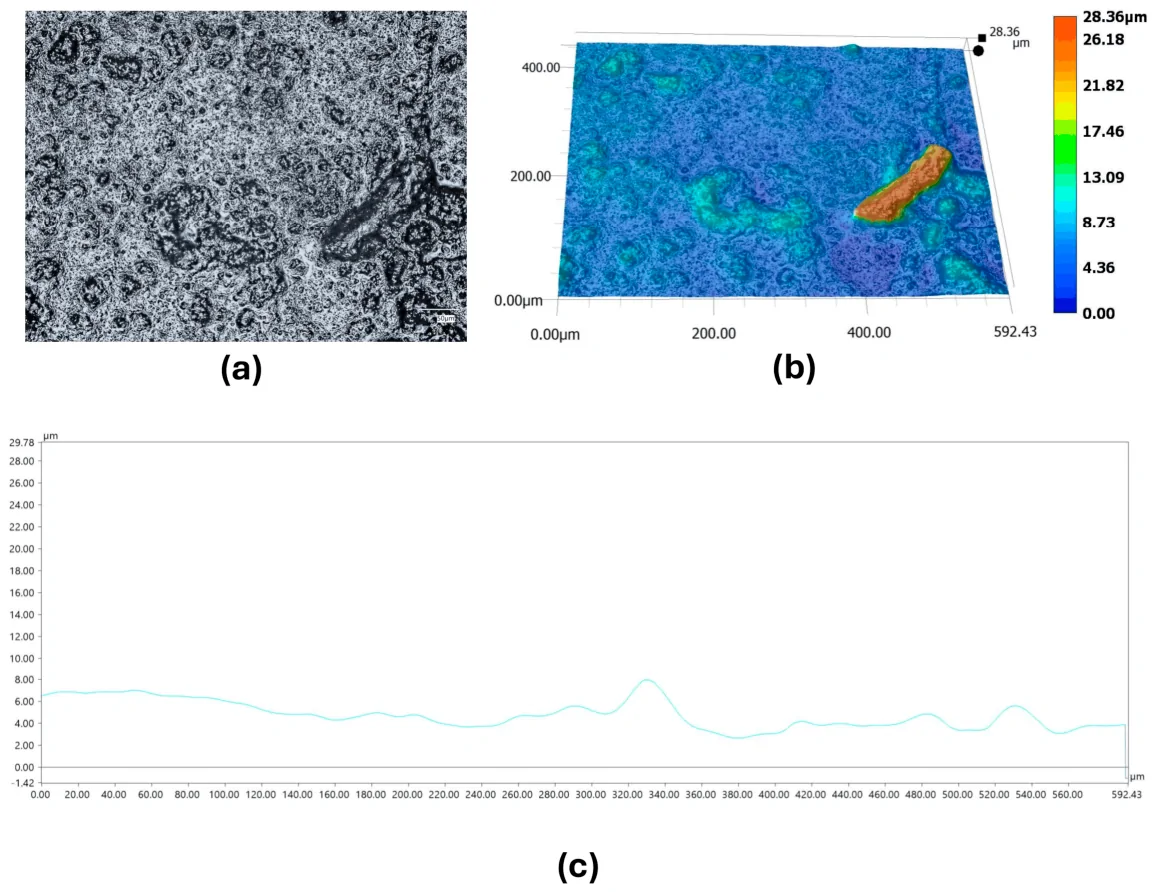
Surface morphology and topography of Hl_0.5 after accelerated abiotic weathering test: (**a**) two-dimensional optical micrograph recorded at 500× magnification; (**b**) three-dimensional surface topography with a colour-coded height scale; (**c**) representative line profile recorded across the analysed surface.

Following the abiotic degradation experiments, changes in surface topography were evaluated using the profile roughness parameters Ra, Rq and Rsk. Ra represents the arithmetic mean deviation of the profile from the mean line, while Rq is more sensitive to pronounced peaks and valleys. Rsk describes the asymmetry of the height distribution, indicating whether the surface profile is dominated by peaks or depressions [73,74]. Following 100 h of abiotic ageing, a marked increase in the Ra parameter was observed for the neat resin, H1_0.5, Nc_0.1, Nc_0.5 and CNTs_0.1 samples, amounting to 463%, 450%, 1573%, 519%, and 150%, respectively. In contrast, Ra decreased by 49% for Nc_0.25. A similar relationship was demonstrated by Bakkaloğlu et al. in thermal ageing experiments, where the Ra parameter decreased significantly in the case of a milled methacrylate composite containing as much as 27% by weight of inorganic filler [75]. For ZnO_0.1, no substantial change was detected, with Ra values of 0.96 ± 0.32 µm before ageing and 0.93 ± 0.01 µm after exposure (Table 9). Moreover, 100 h of exposure to abiotic ageing conditions resulted in a pronounced increase in surface irregularities and the formation of local grooves and depressions (Figure 17, Figure 18, Figure 19 and Figures S13–S16). Similar changes were reported by Lukachevskaia et al. [76] for epoxy-matrix laminates exposed to UV radiation for up to 2000 h. Surface profilograms recorded before and after ageing revealed progressively increasing roughness, deepening of microdefects, and the formation and enlargement of pores within the polymer matrix. Comparable observations were also described by Degirmenci et al. for a resin nanoceramic composite subjected to thermocycling and UV ageing. With increasing exposure time, the depth of cracks and surface depressions increased, while progressive degradation of the polymer matrix led to greater exposure of filler particles [77].

Due to the marked heterogeneity of the surfaces under investigation, surface roughness parameters, particularly Sa and Sq, provide a more representative description of surface topography than parameters calculated on the basis of a single linear profile. Sa reflects the mean absolute deviation of surface height from the mean plane, whilst Sq places greater emphasis on pronounced variations in height. The Sz parameter additionally describes the total vertical distance between the highest elevation and the deepest valley in the analysed area.

Following accelerated abiotic ageing, the pure resin showed a significant increase in the values of Sa, Sq and Sz, amounting to 558%, 743% and 555% respectively. This trend was consistent with the increase observed for the profile parameter Ra and confirms a widespread deterioration in surface condition, encompassing both a general increase in roughness and the formation of pronounced local defects. In the case of ZnO_0.1, a different relationship was observed. Although the Ra remained almost unchanged before and after ageing, the Sa and Sq values increased by 527% and 500% respectively, whilst the Sz value increased by only 19% (Table S1). This apparent discrepancy can be attributed to the spatial heterogeneity of the older surface. As the Ra value is determined along a single selected profile, it may not account for irregularities located outside the measured line. In contrast, Sa and Sq are calculated on the basis of the entire three-dimensional surface, thereby more effectively revealing distributed variations in surface height. Furthermore, the relatively small increase in Sz suggests that ageing did not result in the formation of significantly deeper individual defects, but rather increased the number and spatial distribution of moderate surface irregularities.

## 4. Discussion

UPRs are among the most commonly used thermosetting resins in the composites industry; however, their main limitation remains their relatively low resistance to long-term exposure to environmental factors and their limited mechanical strength compared with modern composites modified with nanofillers. For this reason, in recent years, increasing attention has been paid to the use of nanoadditives as a means of improving both the mechanical properties and service life of polymeric materials. However, most publications analyse a single type of nanofiller, whilst direct comparisons of several different nanomaterials in an identical polymer matrix are relatively rare. In this study, five groups of nanoadditives, differing in structure, morphology and chemical nature, were compared, making it possible to determine how the type of nanofiller influences the properties of an orthophthalic polyester resin. These structural differences are particularly important because the reinforcing efficiency of a nanofiller is determined not only by its concentration, but also by its shape, ratio, specific surface characteristics and the nature of the polymer–filler interface.

FTIR analysis did not reveal significant changes in the characteristic chemical structure of the polymer matrix following the incorporation of the nanofillers. For all composites, the spectra were dominated by the characteristic absorption bands of the crosslinked polyester resin, and no distinct new absorption bands were observed. However, considering the relatively low nanofiller concentrations (0.1–0.5 wt.%) and the substantial overlap between filler-related signals and the characteristic absorption bands of the polyester matrix, the absence of new bands cannot be considered conclusive evidence for the absence of chemical bonding at the polymer–nanofiller interface. Therefore, the FTIR results indicate only that no significant chemical changes or distinct spectroscopic evidence of new bond formation were detected under the experimental conditions employed. Consequently, the observed improvement in the properties of the nanocomposites cannot be directly attributed to the formation of new chemical bonds based on the FTIR results alone. Physical and interfacial interactions between the nanofillers and the polymer network, including the restriction of polymer chain mobility and stress transfer at the polymer–filler interface, may contribute to the observed reinforcement. Such mechanisms are commonly reported for polymer nanocomposites, although their relative contribution depends strongly on filler morphology, dispersion, interfacial compatibility, and filler concentration [78,79]. This is confirmed by SEM observations. The surfaces of all samples after curing were relatively homogeneous, and the local irregularities observed were mainly due to the casting and curing processes. However, significantly greater variations were observed on the fracture surfaces. Compared with the pure resin, the fracture surfaces of the composites were characterised by a more complex morphology, a greater number of discontinuities and changes in the direction of crack propagation. This structure indicates that crack propagation required more energy, as the nanoparticles acted as an obstacle to the propagating crack. This phenomenon corresponds well with the results of compressive strength tests, in which all nanoadditives led to an increase in strength compared with the pure resin. At the same time, the results obtained show that the increase in mechanical strength was not proportional to the increase in nano-filler content. For some of the materials, an optimal additive concentration was observed; beyond this concentration, no further improvement in properties occurred, or the improvement was significantly smaller. This was particularly noticeable in composites containing CNTs, Cloisite^®^ 30Band nanoclay. This effect is often associated with a deterioration in the dispersion of nanoparticles at higher concentrations, leading to the formation of potential local agglomerates that act as stress concentration points. Although no quantitative analysis of the dispersion of nanoadditives was carried out in this study, the observed trend in strength changes is consistent with the mechanisms described in the literature [80]. Therefore, potential local agglomeration is considered here only as a possible mechanism contributing to the observed non-linear concentration dependence and not as an experimentally confirmed cause. A. Seetharaman and V. Narayanan also observed that the addition of nancolay to polyester composites enhances mechanical properties up to a certain point, after which, once a given amount is exceeded, these properties begin to decline [81]. The results of the pull-off tests deserve particular attention. Detaching the metal dolly from the specimen made it possible to determine the failure mechanism or provided information on the structural integrity of the composites. In most samples containing higher amounts of nanofillers, cohesive failure was observed, involving the tearing away of a fragment of the composite together with the measuring dolly. However, it should be emphasised that the pull-off values must be interpreted together with the observed failure mode. In samples exhibiting adhesive failure, the observed pull-off stress primarily represents the strength of the adhesive-composite interface and therefore cannot be regarded as a direct measure of the intrinsic cohesive strength of the composite. Consequently, the relatively low values obtained for the pure resin and several composites, do not necessarily indicate lower intrinsic material strength, as failure occurred before the cohesive strength of the composite was reached. In contrast, for samples exhibiting cohesive failure, fracture occurred within the composite, indicating that the adhesive bond exceeded the local strength of the near-surface composite layer. Therefore, direct quantitative comparison of pull-off values corresponding to different failure modes should be made with caution. The observed pull-off results are consistent with SEM observations of fracture surfaces and with the results of compressive strength tests, confirming that the nanofillers influenced the mode of damage propagation within the material.

In the case of environmental stability tests, it was observed that, despite incubation in solutions with pH values of 4, 7 and 9, the changes in mass of all samples were minor and, in most cases, did not exceed 1%. At the same time, potentiometric measurements revealed only slight changes in the pH of the solutions throughout the entire incubation period. These two sets of results complement one another. The slight change in mass indicates limited sorption of liquid by the material, whilst the stability of the pH suggests that no significant quantities of products, reagent residues, degradation products or other substances capable of altering the pH of the environment were released from the composites. This means that, despite contact with solutions of varying pH, the composites under investigation retained good chemical stability. The differences observed between nanofillers can also be considered in the context of their morphology and surface properties. Layered materials, such as nanoclay and Cloisite^®^ 30B, may increase the tortuosity of diffusion paths through the polymer matrix, potentially limiting the transport of water and solutes. The hydroxylated surfaces of halloysite and nanoclay may promote interactions with water. These competing effects may contribute to the slight differences observed between the samples; however, as neither the water diffusion coefficients nor the distribution of the fillers were determined in this study, these mechanisms should be regarded as possible interpretations rather than directly demonstrated effects. In the case of ageing tests, most composites retained high compressive strength after short-term exposure to UV radiation, periodic water spraying, and moisture condensation., Furthermore, for some materials, these values were even higher than before the ageing process. Hovewer, this increase should not be attributed only to resistance to short-term, accelerated abiotic ageing. It should be noted that the ageing procedure involved exposure to 60 °C during UV irradiation and 50 °C during the condensation stage, conditions that may also have promoted additional post-curing of the unsaturated polyester resin matrix [82,83]. Previous studies showed that heat treating polyester resins at temperatures of 40–60 °C can increase the degree of conversion and significantly affect their mechanical properties [84]. During the first heating cycle carried out as part of the DSC tests, residual curing enthalpy was observed for most samples, with the exception of the pure resin and ZnO_0.1. This indicates that the introduction of a filler into orthophthalic polyester during cross-linking leaves some of the reactive groups necessary for curing. Consequently, the mechanical properties measured after ageing may reflect the combined effects of changes induced by UV radiation and moisture, as well as the continued cross-linking of the polymer network under the influence of heat. Importantly, for all formulations, no direct correlation was observed between residual curing enthalpy and compressive strength after ageing. For example, the ZnO_0.1 showed no detectable residual curing enthalpy, but after ageing achieved one of the highest compressive strength values of 281. 6 ± 1.1 MPa, which was 0.85% than that of the pure resin. Thus, secondary curing may have contributed to the observed increase in strength in selected materials, but cannot fully explain the mechanical response. The residual curing enthalpy, determined by the DSC method, has previously been used to characterise incomplete cross-linking and the subsequent secondary curing of unsaturated polyester resins [85]. The results of the tribological tests are also noteworthy. Materials characterised by greater internal cohesion and higher mechanical strength also exhibited more favourable tribological properties. This suggests that wear resistance was related not only to the hardness of the material, but also to its microstructure and its ability to limit the propagation of microcracks during friction. This relationship is consistent with SEM observations of the surfaces following tribological testing, where samples with lower wear also exhibited less developed friction scar topography [86].

The research carried out has demonstrated that the effectiveness of nanomodification of orthophthalic polyester resin depends primarily on the type of nanofiller used and the mechanism of its interaction with the matrix. The comparison of the five nanofillers indicates that their performance is governed by different combinations of morphology, surface characteristics and interfacial effects rather than by filler concentration alone. CNTs can provide reinforcement through their high aspect ratio and potential stress-transfer capability. Layered nanoclay and Cloisite^®^ 30B may combine mechanical reinforcement with modification of transport pathways; tubular halloysite can interfere with local deformation and crack propagation; whereas particulate ZnO provides a large polymer–particle interface and may additionally influence the response of the composite to UV exposure. This mechanistic diversity explains why no single nanofiller exhibited the optimum performance in all of the investigated tests. The results of all the analyses carried out complement one another and indicate that the mechanical properties, environmental resistance and tribological behaviour are associated with changes in the material’s microstructure and polymer–filler interfacial interactions, rather than being attributable solely to chemical modification. From a practical point of view, this means that the choice of nanoadditive should depend on the intended application of the composite—in applications requiring high strength, ZnO appears to be the most suitable.

## 5. Conclusions

This study investigated the effect of selected nanofillers on the properties of UPR-based composites. The research carried out demonstrated that the use of nanofillers is an effective method of modifying the properties of UPR; however, the extent of the achieved improvement depends on the type and content of the nanofiller. The results indicate that there is no single nanoadditive capable of simultaneously improving all the properties analysed. Composites containing ZnO exhibited the highest mechanical strength and retained favourable mechanical properties after accelerated weathering, while Cloisite^®^ 30B-containing composites showed relatively small changes under the environmental exposure conditions investigated in this study. Halloysite showed relatively stable mechanical performance under the investigated conditions, whilst the effectiveness of CNTs was strongly dependent on their content and may additionally have been influenced by their dispersion within the polymer matrix. The results obtained provide comparative information that may support the selection of an appropriate nanofiller depending on the intended application of the composite and confirm that a comprehensive assessment using multiple testing methods allows for a more complete characterisation of polymeric materials. At the same time, the results obtained point to the need for further research involving long-term exposure of materials under real operating conditions, an assessment of the effect of higher concentrations of nanoadditives, and a detailed analysis of their dispersion in the polymer matrix. This will provide a better understanding of the mechanisms responsible for the observed changes in properties and help to determine the optimal design conditions for durable polyester composites intended for engineering applications.

## Figures and Tables

**Table 1:** Changes in the mass [%] of the samples following incubation in buffers at pH 4, 7 and 9, and following soil burial tests (*n* = 3).

Sample	After Incubation in a Buffer with a pH of 4	After Incubation in a Buffer with a pH of 7	After Incubation in a Buffer with a pH of 9	After Soil Burial
Pure resin	0.282 ± 0.006	0.336 ± 0.013	0.363 ± 0.004	0.312 ± 0.012
Hl_0.1	0.364 ± 0.014	0.246 ± 0.001	0.009 ± 0.001	0.184 ± 0.030
Hl_0.25	0.399 ± 0.027	0.251 ± 0.003	0.209 ± 0.009	0.166 ± 0.013
Hl_0.5	0.384 ± 0.007	0.636 ± 0.696	0.191 ± 0.013	0.479 ± 0.121
Nc_0.1	0.569 ± 0.019	0.306 ± 0.005	0.183 ± 0.012	0.212 ± 0.019
Nc_0.25	0.563 ± 0.071	0.493 ± 0.126	0.274 ± 0.003	0.310 ± 0.050
Nc_0.5	0.790 ± 0.166	0.338 ± 0.023	0.201 ± 0.011	0.214 ± 0.009
CNTs_0.1	0.903 ± 0.104	0.254 ± 0.007	0.087 ± 0.006	−2.223 ± 0.147
CNTs_0.25	0.430 ± 0.032	0.389 ± 0.076	0.316 ± 0.141	0.230 ± 0.026
CNTs_0.5	0.542 ± 0.292	0.244 ± 0.016	−0.036 ± 0.031	2.560 ± 0.331
Cl_0.1	0.271 ± 0.499	0.536 ± 0.008	0.584 ± 0.240	−1.312 ± 0.409
Cl_0.25	−0.045 ± 1.662	0.468 ± 0.009	0.275 ± 0.016	0.254 ± 0.040
Cl_0.5	0.653 ± 0.047	0.562 ± 0.002	0.451 ± 0.032	−0.118 ± 0.025
ZnO_0.1	0.445 ± 0.218	0.403 ± 0.048	0.370 ± 0.047	0.395 ± 0.079
ZnO_0.25	0.328 ± 0.061	0.377 ± 0.084	0.116 ± 0.000	0.006 ± 0.009
ZnO_0.5	0.488 ± 0.010	0.449 ± 0.019	0.304 ± 0.011	0.295 ± 0.039

**Table 2:** Contact angle values measured using water and diiodomethane, and the corresponding surface free energy ($\gamma_{s}$) with its dispersive ($\gamma_{s}^{D}$) and polar ($\gamma_{s}^{P}$) components for neat unsaturated polyester resin and nanofiller-modified composites (*n* = 3).

Sample	Water [°]	Diiodomethane [°]	$\gamma_{s}^{D}$ [mN·m^−1^]	$\gamma_{s}^{P}$ [mN·m^−1^]	$\gamma_{s}$ [mN·m^−1^]
Pure resin	102.95 ± 3.31	40.60 ± 2.55	41.93 ± 1.85	0.19 ± 0.24	42.12 ± 2.01
Hl_0.1	103.54 ± 2.65	48.41 ± 2.35	37.01 ± 1.73	0.08 ± 0.10	37.09 ± 1.78
Hl_0.25	101.59 ± 2.92	50.69 ± 3.85	34.94 ± 2.72	0.16 ± 0.19	35.10 ± 2.62
Hl_0.5	104.98 ± 2.32	47.12 ± 0.87	38.27 ± 0.85	0.12 ± 0.09	38.40 ± 0.93
Nc_0.1	97.57 ± 1.83	47.40 ± 3.19	36.07 ± 2.13	0.30 ± 0.20	36.37 ± 1.99
Nc_0.25	95.22 ± 1.23	49.88 ± 0.94	33.84 ± 0.70	0.71 ± 0.23	34.55 ± 0.57
Nc_0.5	98.13 ± 1.42	51.87 ± 1.29	33.27 ± 0.94	0.40 ± 0.18	33.66 ± 0.83
CNTs_0.1	93.52 ± 3.95	41.08 ± 1.79	38.97 ± 1.55	0.67 ± 0.50	39.64 ± 1.18
CNTs_0.25	96.16 ± 1.49	45.32 ± 1.71	37.05 ± 1.17	0.36 ± 0.18	37.41 ± 1.06
CNTs_0.5	95.37 ± 1.68	43.32 ± 1.52	38.10 ± 1.06	0.38 ± 0.22	38.48 ± 0.93
Cl_0.1	78.72 ± 3.41	30.88 ± 3.17	40.34 ± 1.82	3.91 ± 1.33	44.25 ± 1.27
Cl_0.25	98.78 ± 2.05	50.24 ± 1.17	34.52 ± 0.95	0.28 ± 0.24	34.80 ± 0.80
Cl_0.5	86.71 ± 5.73	22.06 ± 2.67	46.55 ± 2.02	1.22 ± 1.26	47.77 ± 1.08
ZnO_0.1	78.54 ± 1.14	29.42 ± 1.61	41.01 ± 0.83	3.73 ± 0.48	44.74 ± 0.62
ZnO_0.25	98.73 ± 3.31	41.43 ± 1.14	40.23 ± 1.18	0.15 ± 0.09	40.38 ± 1.12
ZnO_0.5	77.75 ± 1.69	51.91 ± 2.13	28.18 ± 1.38	7.39 ± 1.02	35.57 ± 1.03

**Table 3:** Changes in the surface roughness parameters (Ra and Rz) of neat unsaturated polyester resin and nanofiller-modified composites after incubation in buffer solutions (pH 4, 7, and 9) and after soil burial (*n* = 4).

Sample	Before Incubation		After Incubation in a Buffer with a pH of 4		After Incubation in a Buffer with a pH of 7		After Incubation in a Buffer with a pH of 9		After Soil Burial	
	Ra [µm]	Rz [µm]	Ra [µm]	Rz [µm]	Ra [µm]	Rz [µm]	Ra [µm]	Rz [µm]	Ra [µm]	Rz [µm]
Pure resin	0.27 ± 0.05	1.65 ± 0.31	1.25 ± 0.25	5.48 ± 1.16	0.42 ± 0.10	2.30 ± 0.54	0.44 ± 0.09	2.00 ± 0.38	0.53 ± 0.04	2.88 ± 0.43
Hl_0.1	0.63 ± 0.12	3.13 ± 0.54	1.57 ± 0.36	9.44 ± 4.59	0.33 ± 0.10	1.81 ± 0.46	0.31 ± 0.04	1.50 ± 0.44	0.83 ± 0.28	3.74 ± 1.02
Hl_0.25	0.28 ± 0.02	1.70 ± 0.24	1.06 ± 0.15	6.95 ± 1.73	0.74 ± 0.22	3.68 ± 1.15	0.63 ± 0.06	3.23 ± 0.36	0.47 ± 0.18	3.03 ± 1.46
Hl_0.5	0.26 ± 0.06	1.55 ± 0.36	0.67 ± 0.23	3.36 ± 0.49	0.73 ± 0.16	3.87 ± 0.81	0.57 ± 0.16	3.19 ± 1.16	0.50 ± 0.08	3.10 ± 1.16
Nc_0.1	0.26 ± 0.09	1.49 ± 0.44	0.59 ± 0.14	4.59 ± 1.82	0.37 ± 0.09	1.77 ± 0.26	0.51 ± 0.24	3.39 ± 2.48	0.37 ± 0.03	1.97 ± 0.25
Nc_0.25	1.10 ± 0.06	4.65 ± 0.17	0.47 ± 0.10	2.37 ± 0.50	0.34 ± 0.06	1.60 ± 0.27	0.34 ± 0.06	2.46 ± 0.48	0.35 ± 0.11	1.81 ± 0.49
Nc_0.5	0.34 ± 0.07	1.90 ± 0.55	1.15 ± 0.35	7.83 ± 2.16	0.67 ± 0.14	5.36 ± 2.53	0.61 ± 0.19	4.56 ± 2.12	0.37 ± 0.09	2.01 ± 0.43
CNTs_0.1	0.30 ± 0.04	1.39 ± 0.27	1.03 ± 0.17	5.20 ± 0.98	0.29 ± 0.10	1.77 ± 0.65	0.50 ± 0.13	2.92 ± 1.00	0.52 ± 0.15	2.62 ± 1.02
CNTs_0.25	0.60 ± 0.44	3.17 ± 2.32	0.75 ± 0.18	4.88 ± 0.95	0.30 ± 0.07	2.13 ± 0.84	0.43 ± 0.07	2.20 ± 0.42	0.32 ± 0.07	1.84 ± 0.33
CNTs_0.5	0.41 ± 0.10	2.07 ± 0.38	0.66 ± 0.11	3.62 ± 0.70	0.67 ± 0.11	4.31 ± 0.35	1.03 ± 0.24	6.28 ± 1.74	0.27 ± 0.06	1.65 ± 0.41
Cl_0.1	0.46 ± 0.11	2.97 ± 0.91	0.39 ± 0.13	1.96 ± 0.75	0.56 ± 0.15	4.04 ± 1.39	2.36 ± 0.28	11.78 ± 1.35	0.82 ± 0.22	3.19 ± 0.57
Cl_0.25	0.62 ± 0.23	4.52 ± 2.08	2.47 ± 0.36	13.81 ± 3.00	0.39 ± 0.12	2.89 ± 1.47	0.62 ± 0.21	3.56 ± 0.76	0.49 ± 0.10	3.04 ± 0.56
Cl_0.5	0.72 ± 0.07	4.85 ± 1.02	0.98 ± 0.14	11.59 ± 5.39	0.61 ± 0.19	4.95 ± 3.34	1.44 ± 0.18	17.07 ± 4.74	1.03 ± 0.28	7.08 ± 4.21
ZnO_0.1	0.96 ± 0.32	5.38 ± 1.23	2.21 ± 0.25	22.86 ± 4.25	0.33 ± 0.05	1.86 ± 0.40	0.18 ± 0.04	1.03 ± 0.25	0.45 ± 0.07	3.52 ± 0.62
ZnO_0.25	2.65 ± 0.61	20.47 ± 3.74	0.73 ± 0.10	5.45 ± 0.95	4.09 ± 0.60	26.62 ± 3.58	1.32 ± 0.42	6.74 ± 1.24	0.49 ± 0.10	3.04 ± 0.56
ZnO_0.5	0.80 ± 0.11	7.52 ± 0.59	1.39 ± 0.29	13.92 ± 6.27	0.89 ± 0.31	8.18 ± 3.87	2.15 ± 0.32	21.49 ± 4.33	0.75 ± 0.27	5.40 ± 2.70

**Table 4:** Thermal degradation parameters obtained from TGA and DTG analyses of the neat resin and composites containing different types and concentrations of nanofillers: residual mass at 600 °C after 15 min of oxidation in air at 600 °C, temperature corresponding to 5% mass loss (T_d,5%_), and temperature of the maximum degradation rate (T_max,DTG_). Data are presented as mean ± standard deviation (*n* = 3).

Sample	Residual Mass at 600 °C in N_2_ [%]	Residual Mass After 15 min of Oxidation at 600 °C in Air [%]	T_d,5%_ [°C]	T_max,DTG_ [°C]
Pure resin	20.9 ± 7.3	18.3 ± 7.7	272.0 ± 8.3	396.5 ± 0.7
Nc_0.1	19.9 ± 0.4	17.6 ± 1.7	239.2 ± 15.9	396 ± 81.9
Nc_0.25	25.1 ± 0.2	20.3 ± 6.3	242.4 ± 45.2	399.3 ± 1.6
Nc_0.5	14.4 ± 6.6	11.0 ± 6.1	258.3 ± 15.8	397.9 ± 4.3
CNTs_0.1	10.5 ± 5.6	6.1 ± 7.0	247.4 ± 7.1	397.4 ± 1.0
CNTs_0.25	8.6 ± 4.0	5.1 ± 3.6	249.1 ± 42.6	398.5 ± 0.9
CNTs_0.5	17.8 ± 3.5	14.3 ± 4.4	232.2 ± 12.0	398.3 ± 0.2
Cl_0.1	9.8 ± 6.0	9.3 ± 3.5	233.3 ± 8.7	395.8 ± 3.7
Cl_0.25	20.4 ± 9.0	17.0 ± 7.7	282.6 ± 14.3	393.6 ± 2.5
Cl_0.5	10.9 ± 4.4	6.2 ± 4.2	246.8 ± 4.6	391.2 ± 0.5
Hl_0.1	21.1 ± 3.1	19.6 ± 3.8	280.9 ± 0.0	400.7 ± 1.8
Hl_0.25	17.9 ± 3.1	15.5 ± 3.0	244.8 ± 17.4	398.6 ± 0.4
Hl_0.5	27.6 ± 3.7	25.8 ± 2.9	285.3 ± 11.7	398.7 ± 1.1
ZnO_0.1	24.4 ± 5.4	21.6 ± 6.0	278.9 ± 11.7	392.9 ± 0.7
ZnO_0.25	17.0 ± 4.6	14.5 ± 4.4	252.5 ± 8.3	391.4 ± 1.6
ZnO_0.5	18.5 ± 2.8	15.4 ± 3.1	248.7 ± 0.0	393.6 ± 2.3

**Table 5:** DSC parameters determined during the first heating cycle and the glass transition temperature determined during the second heating cycle, where res—residual cross-linking (post-curing); Tg—glass transition temperature determined as the midpoint of the transition during the second heating.

Sample	T,res [°C]	ΔHres [J g^−1^]	Tg, [°C]
Pure resin	-	-	108.9
Nc_0.1	96.6	13.66	111.1
Nc_0.25	96.6	15.65	105.2
Nc_0.5	97.9	18.55	110.7
CNTs_0.1	96.7	19.67	104.2
CNTs_0.25	123.5	2.189	106.3
CNTs_0.5	118.7	5.720	114.5
Cl_0.1	116.7	6.463	104.4
Cl_0.25	120.0	6.392	75.9
Cl_0.5	116.8	6.891	107.8
Hl_0.1	117.3	7.175	110.5
Hl_0.25	117.5	4.018	106.7
Hl_0.5	117.2	6.715	114.1
ZnO_0.1	—	—	115.7
ZnO_0.25	119.3	5.121	121.6
ZnO_0.5	121.2	2.632	120.0

**Table 6:** Gross heat of combustion (PCS) of neat resin and resin containing 0.1 wt.% nanoclay.

	Gross Heat of Combustion, PCS [MJ/kg]
Pure resin	29.8395 ± 0.1704
Nc_0.1	28.7640 ± 0.8088

**Table 7:** Pull-off stress values of the investigated polyester nanocomposites together with the observed failure mode (*n* = 3).

Sample	Strength [MPa]	Failure Mode
Pure resin	0.63 ± 0.09	Adhesive
Hl_0.1	0.86 ± 0.05	Adhesive
Hl_0.25	1.30 ± 0.08	Cohesive
Hl_0.5	1.93 ± 0.68	Cohesive
Nc_0.1	0.76 ± 0.05	Adhesive
Nc_0.25	1.20 ± 0.24	Cohesive
Nc_0.5	1.87 ± 0.71	Cohesive
CNTs_0.1	0.80 ± 0.29	Adhesive
CNTs_0.25	0.73 ± 0.09	Adhesive
CNTs_0.5	1.20 ± 0.51	Cohesive
Cl_0.1	0.87 ± 0.05	Adhesive
Cl_0.25	0.63 ± 0.24	Adhesive
Cl_0.5	2.73 ± 0.48	Cohesive
ZnO_0.1	0.56 ± 0.09	Adhesive
ZnO_0.25	0.63 ± 0.19	Adhesive
ZnO_0.5	1.73 ± 0.09	Cohesive

**Table 8:** Comparison of the average CoF, linear wear, and mass loss of the plate and pin for pure resin, Nc_0.1 and ZnO_0.1 (*n* = 3).

Sample	Average CoF	Linear Wear [µm/5000 Cycles]	Plate Mass Loss [g]	Pin Mass Loss [mg]
Pure resin	0.602 ± 0.018	439.00 ± 82.26	0.144 ± 0.026	0.79 ± 0.02
Nc_0.1	0.587 ± 0.012	843.00 ± 91.11	0.251 ± 0.016	1.19 ± 0.08
ZnO_0.1	0.558 ± 0.010	372.00 ± 44.41	0.107 ± 0.010	1.00 ± 0.08

**Table 9:** Profile roughness parameters of the investigated materials after 100 h of short-term, accelerated abiotic weathering test. All measurements were performed with more than three replicates (*n* > 3), and the results are presented as mean ± standard error (SE). Statistical significance was defined as * *p* < 0.05, and ** *p* < 0.01.

Sample	Ra [μm]	Rq [μm]	Rsk [μm]
Pure resin	1.52 ± 0.06	1.95 ± 0.08	0.06 ± 0.07
Hl_0.5	1.43 ± 0.11	1.98 ± 0.18	0.85 ± 0.09
Nc_0.1	1.61 ± 0.19	1.94 ± 0.22	0.45 ± 0.11
Nc_0.25	0.56 ± 0.01 **	0.76 ± 0.02 **	0.82 ± 0.12
Nc_0.5	5.69 ± 0.52 **	6.75 ± 0.61 **	0.47 ± 0.06
CNTs_0.1	0.75 ± 0.02 *	0.99 ± 0.03 *	1.31 ± 0.11 **
ZnO_0.1	0.93 ± 0.01	1.11 ± 0.02 *	−0.21 ± 0.03

## Data Availability

The raw data supporting the conclusions of this article will be made available by the authors on request.

## Supplementary Materials

The following supporting information can be downloaded at: https://www.mdpi.com/article/10.3390/ma19183989/s1.

## Abbreviations

The following abbreviations are used in this manuscript:

CNT/CNTs	Carbon nanotubes
DSC	Differential scanning calorimetry
EFI	Extended Focus Imaging
FT-IR	Fourier transform infrared spectroscopy
MEKP	Methyl ethyl ketone peroxide
OW	Owens-Wendt method
PCS	Potential Calorific Value/Gross Heat of Combustion
SEM	Scanning electron microscopy
SEF	Surface free energy
TGA	Thermogravimetric analysis
UV	Ultraviolet
ZnO	Zinc oxide
