# Supplementary material for: Comparative Study of Nanofiller-Reinforced Orthophthalic Polyester Composites: Mechanical, Surface, Tribological and Environmental Performance
Source: Materials (Basel). 2026 Sep 19;19(18):3989. doi: 10.3390/ma19183989 (PMC13608299; doi:10.3390/ma19183989)
Supplement: Supplementary file 1 [file materials-19-03989-s001.zip › materials-4517402-supplementary.pdf]

materials

Supplementary Materials for

Comparative Study of Nanofiller-Reinforced Orthophthalic Polyester Composites: Mechanical, Surface, Tribological and Environmental Performance

Dominik Stępka [1], Dagmara Słota [2,*], Karina Niziołek [1], Zuzanna Buchwald [3], Kinga Setlak [2], Dariusz Mierzwiński [2], Josef Jampilek [4,5,*], Katarzyna Haraźna [2] and Agnieszka Sobczak-Kupiec [2]

1 Cracow University of Technology, CUT Doctoral School, Faculty of Materials Engineering and Physics, Department of Materials Science, 37 Jana Pawła II Av., 31-864 Krakow, Poland
2 Cracow University of Technology, Faculty of Materials Engineering and Physics, Department of Materials Science, 37 Jana Pawła II Av., 31-864 Krakow, Poland
3 Institute of Chemical Technology and Engineering, Poznan University of Technology, Berdychowo St. 4, 60-965, Poznań, Poland
4 Department of Analytical Chemistry, Faculty of Natural Sciences, Comenius University, Ilkovicova 6, 842 15 Bratislava, Slovakia
5 Department of Chemical Biology, Faculty of Science, Palacky University, Slechtitelu 27, 779 00 Olomouc, Czech Republic
* Correspondence: dagmara.slota@pk.edu.pl (D.S.); josef.jampilek@gmail.com (J.J.)

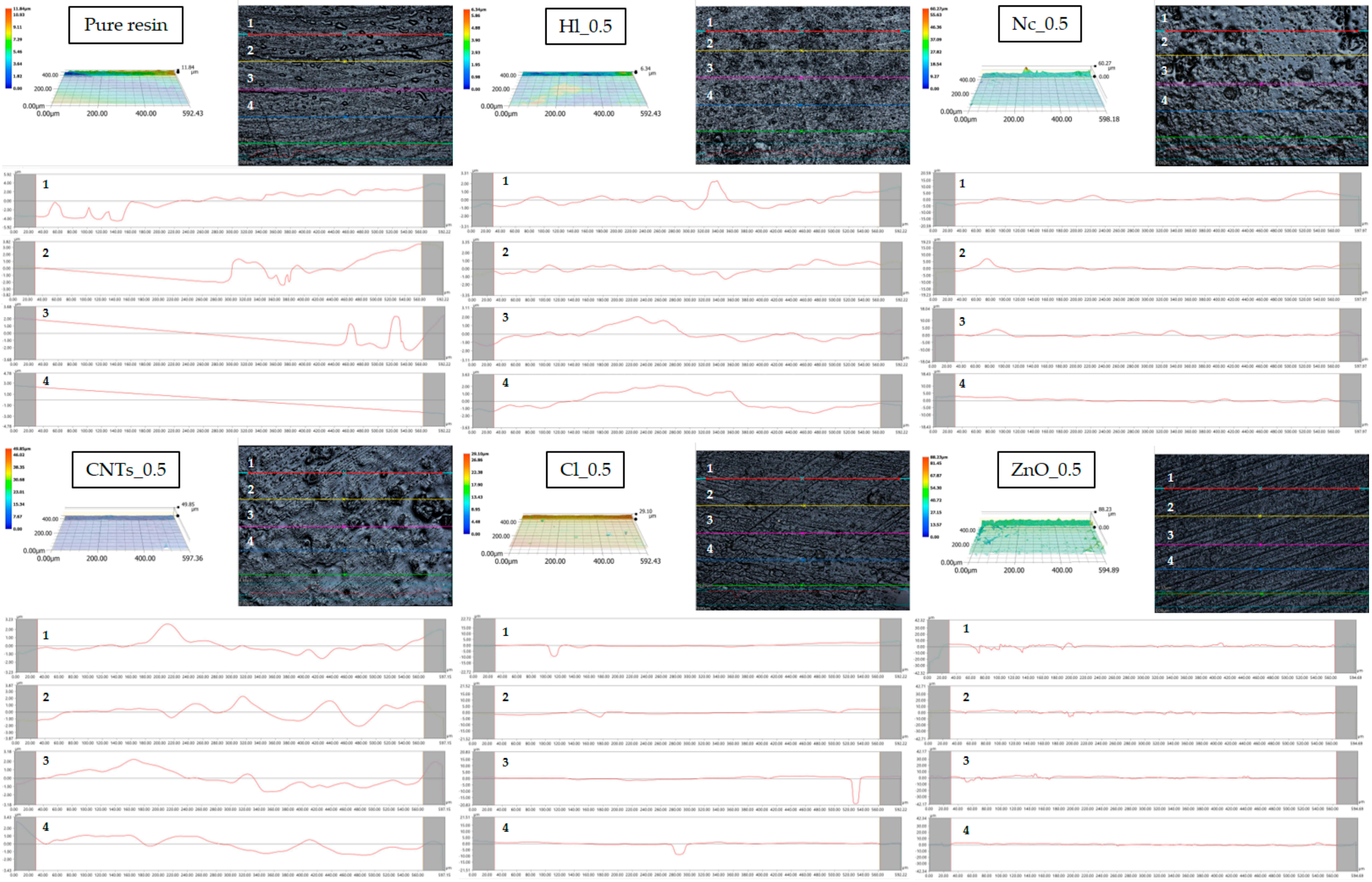

Figure S1: Representative surface topography maps, optical images showing the four measurement traces, and the corresponding roughness profiles of the neat unsaturated polyester resin and

representative composites containing 0.5 wt.% halloysite, nanoclay, CNTs, Cloisite® 30B, and ZnO after incubation in a buffer solution at pH 4.

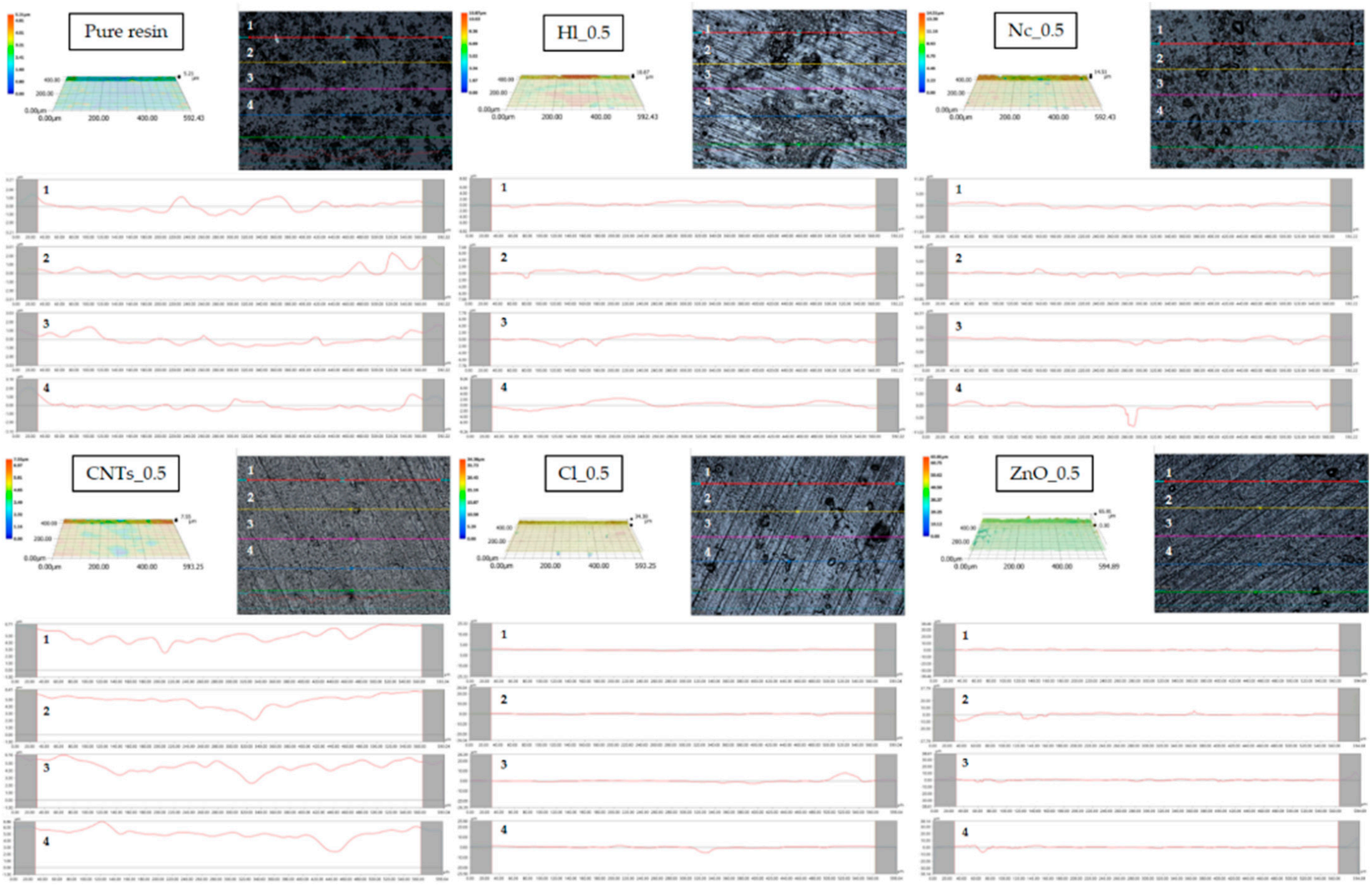

Figure S2: Representative surface topography maps, optical images showing the four measurement traces, and the corresponding roughness profiles of the neat unsaturated polyester resin and representative composites containing 0.5 wt.% halloysite, nanoclay, CNTs, Cloisite® 30B, and ZnO after incubation in a buffer solution at pH 7.

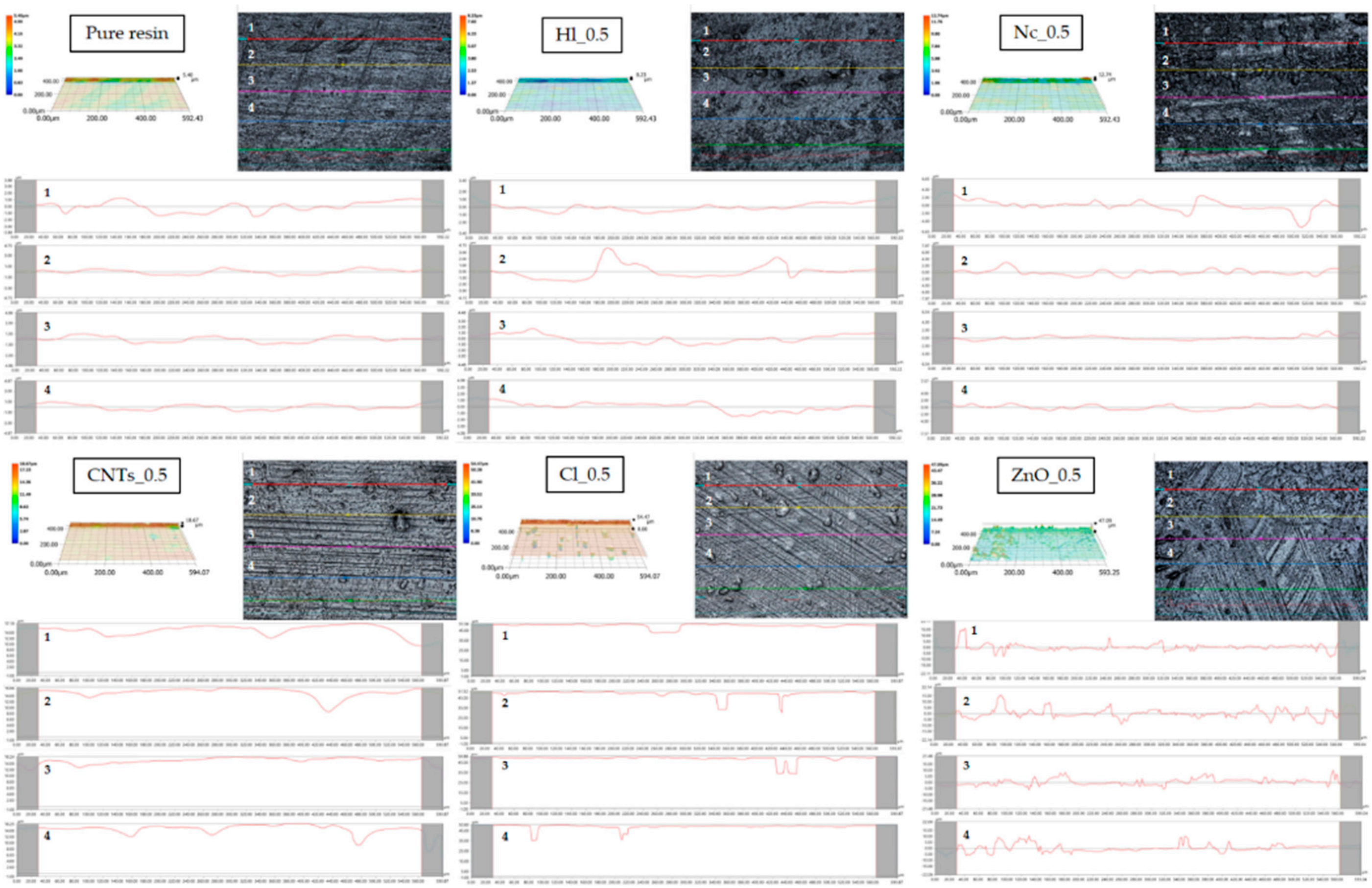

Figure S3: Representative surface topography maps, optical images showing the four measurement traces, and the corresponding roughness profiles of the neat unsaturated polyester resin and representative composites containing 0.5 wt.% halloysite, nanoclay, CNTs, Cloisite® 30B, and ZnO after incubation in a buffer solution at pH 9.

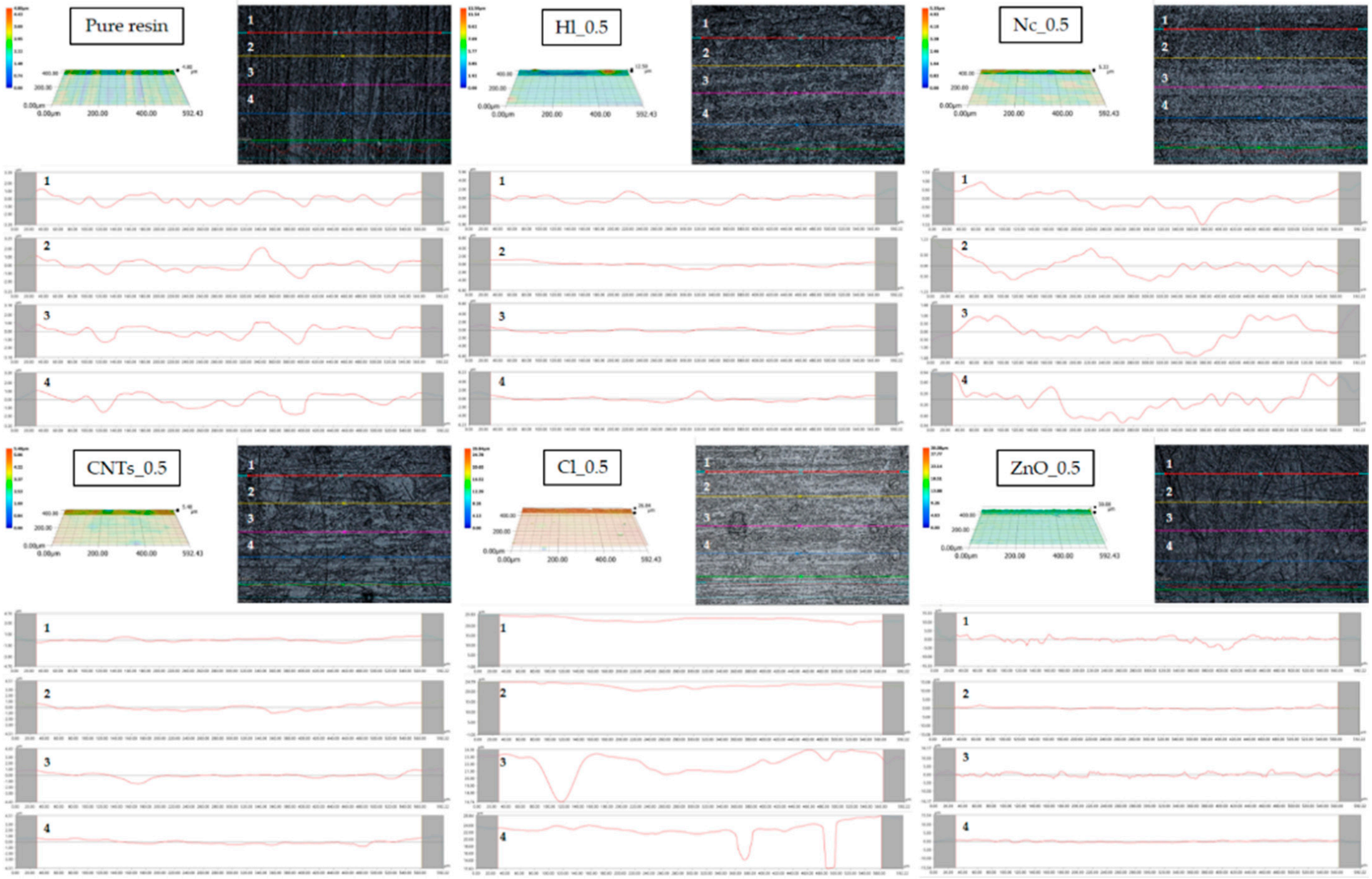

Figure S4: Representative surface topography maps, optical images showing the four measurement traces, and the corresponding roughness profiles of the neat unsaturated polyester resin and representative composites containing 0.5 wt.% halloysite, nanoclay, CNTs, Cloisite® 30B, and ZnO after soil burial.

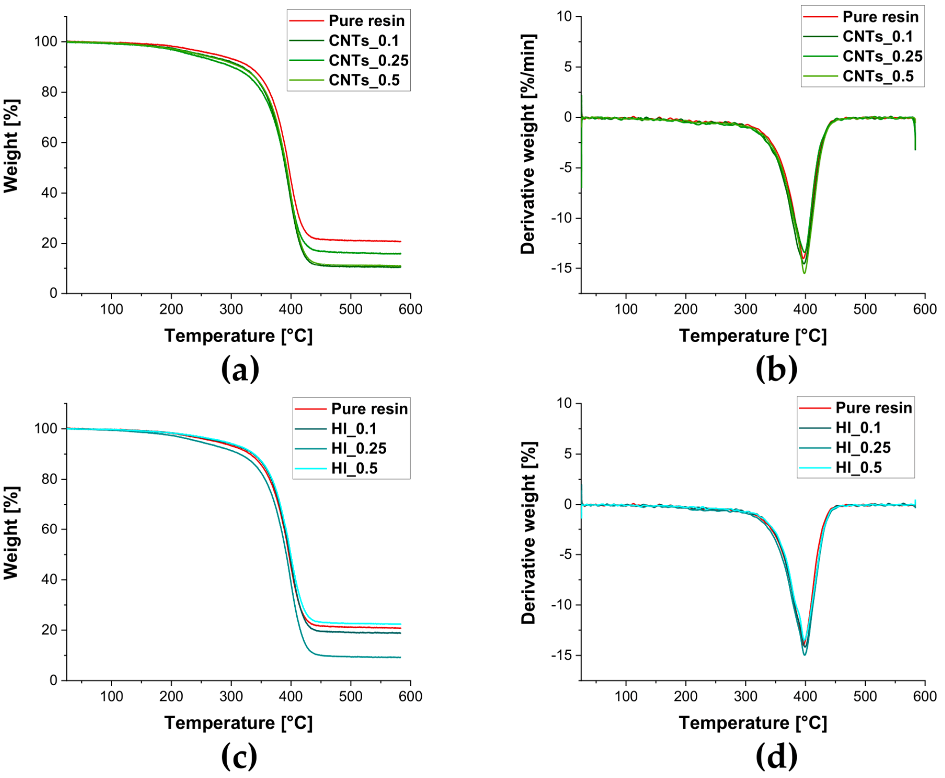

Figure S5: TGA and DTG curves of the pure resin and composites containing different concentrations of CNTs and Halloysite: (a) TGA curves of CNT-containing composites, (b) corresponding DTG curves, (c) TGA curves of Halloysite-containing composites, and (d) corresponding DTG curves.

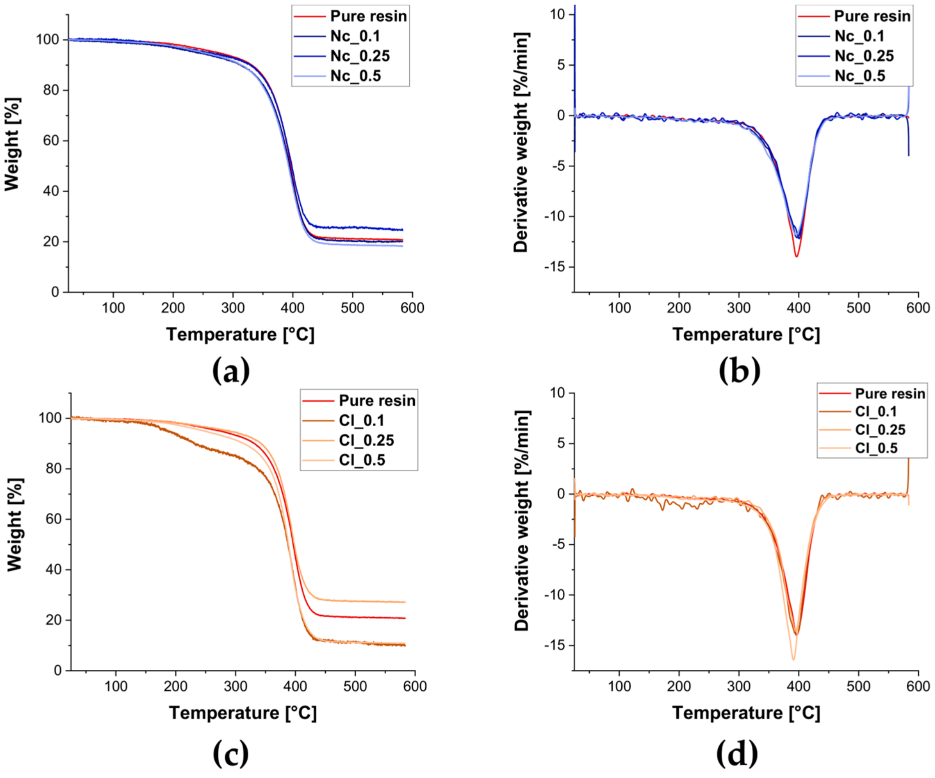

Figure S6: TGA and DTG curves of the neat resin and composites containing different concentrations of Nanoclay and Closite: (a) TGA curves of Nc-containing composites, (b) corresponding DTG curves, (c) TGA curves of Cl-containing composites, and (d) corresponding DTG curves..

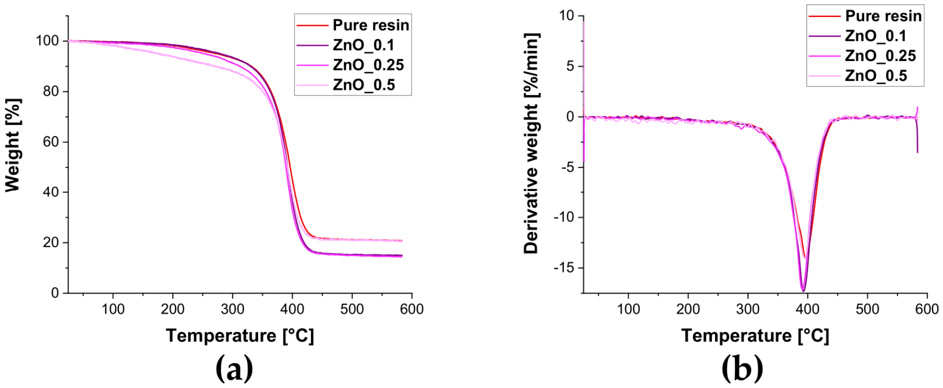

Figure S7: TGA and DTG curves of the neat resin and composites containing different concentrations of zinc oxide nanoparticles: (a) TGA curves of ZnO NPs -containing composites, (b) corresponding DTG curves.

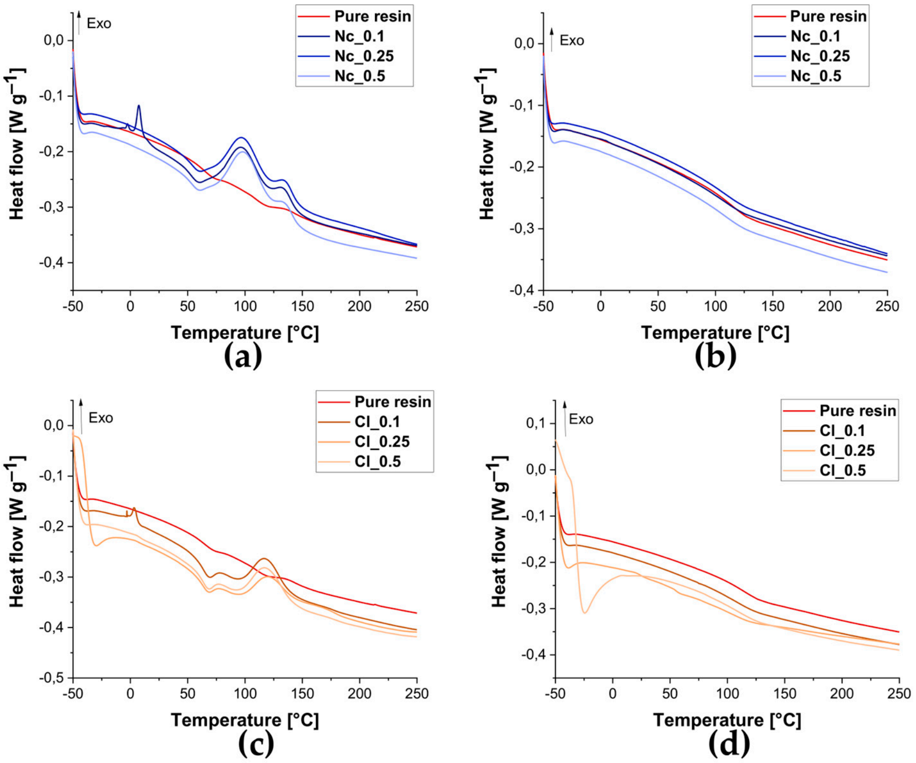

Figure S8. DSC thermograms of the neat resin and composites containing different concentrations of nanoclay and closite: (a) first heating scan of the Nc-containing composites, (b) second heating scan of the Nc-containing composites, (c) first heating scan of the Cl-containing composites, and (d) second heating scan of the Cl-containing composites.

https://doi.org/10.3390/ma19183989

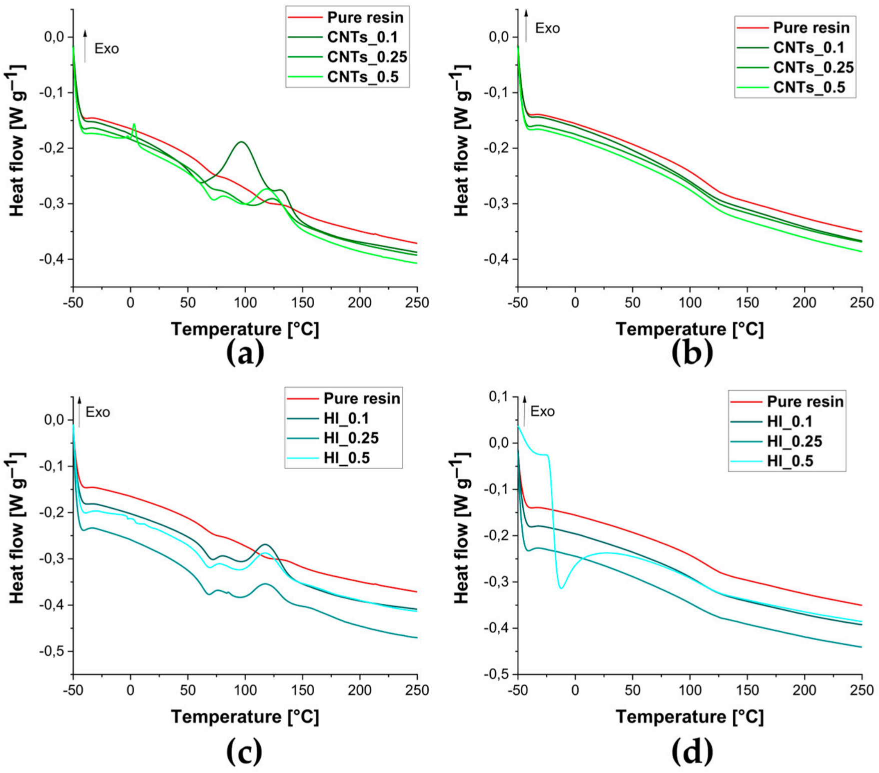

Figure S9: DSC thermograms of the neat resin and composites containing different concentrations of carbon nanotubes and halloysite: (a) first heating scan of the CNTs-containing composites, (b) second heating scan of the CNTs-containing composites, (c) first heating scan of the Hl-containing composites, and (d) second heating scan of the Hl-containing composites.

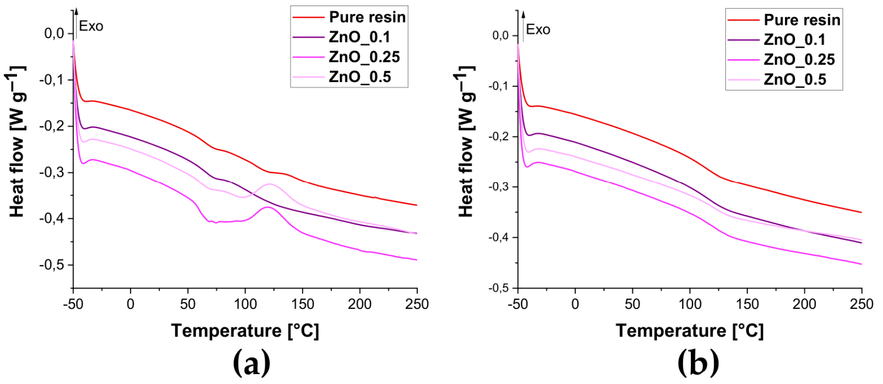

Figure S10: DSC thermograms of the neat resin and composites containing different concentrations of zinc oxide nanoparticles: (a) first heating scan of the ZnO-containing composites, (b) second heating scan of the ZnO-containing composites.

https://doi.org/10.3390/ma19183989

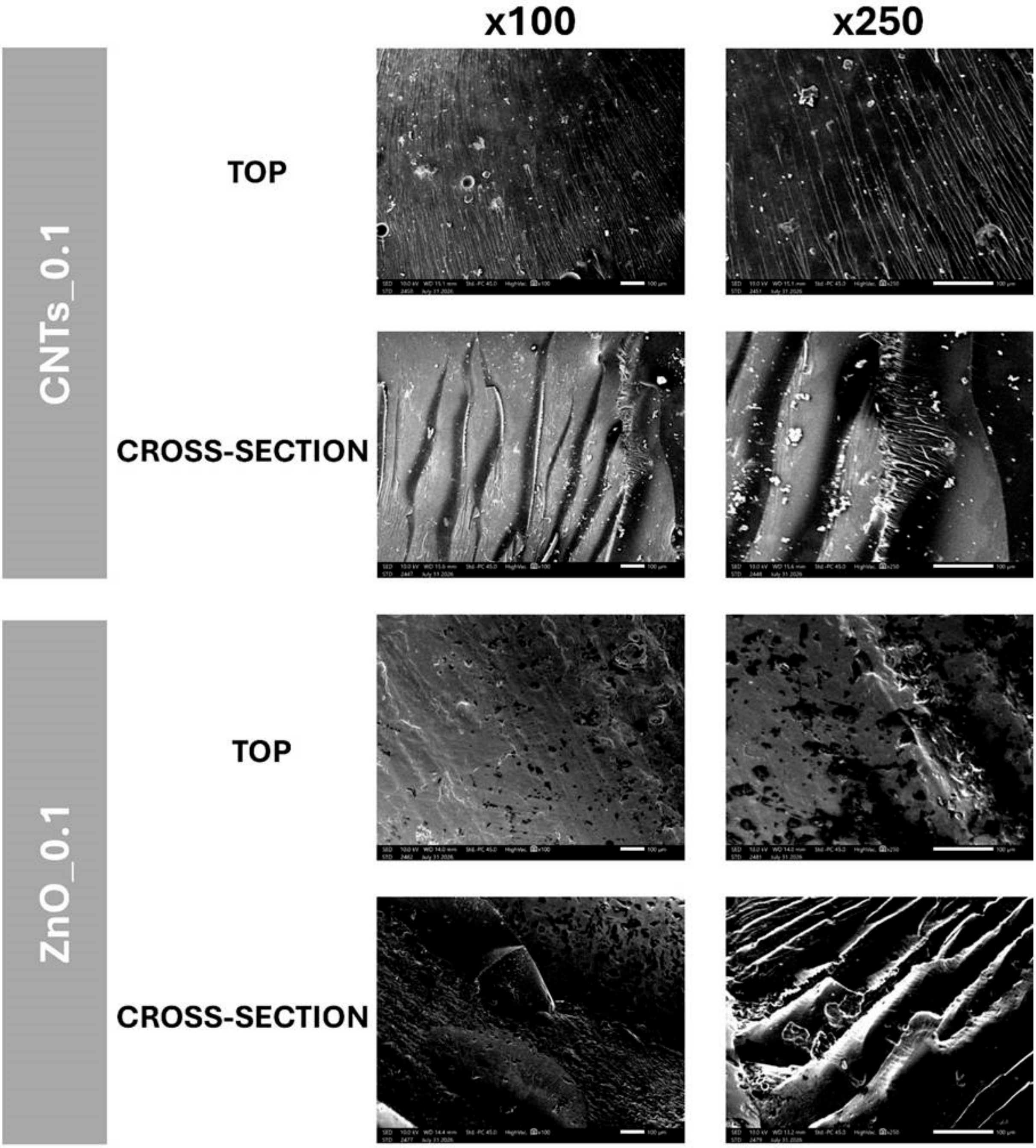

Figure S11: Representative SEM micrographs of the top surfaces and cross-sectional fracture surfaces of the neat resin and selected nanocomposites following 100 h of exposure to accelerated abiotic aging conditions: (a) CNTs_0.1, (b) ZnO_0.1. Micrographs were acquired at magnifications of 100× and 250×. Scale bars: 100 μm.

https://doi.org/10.3390/ma19183989

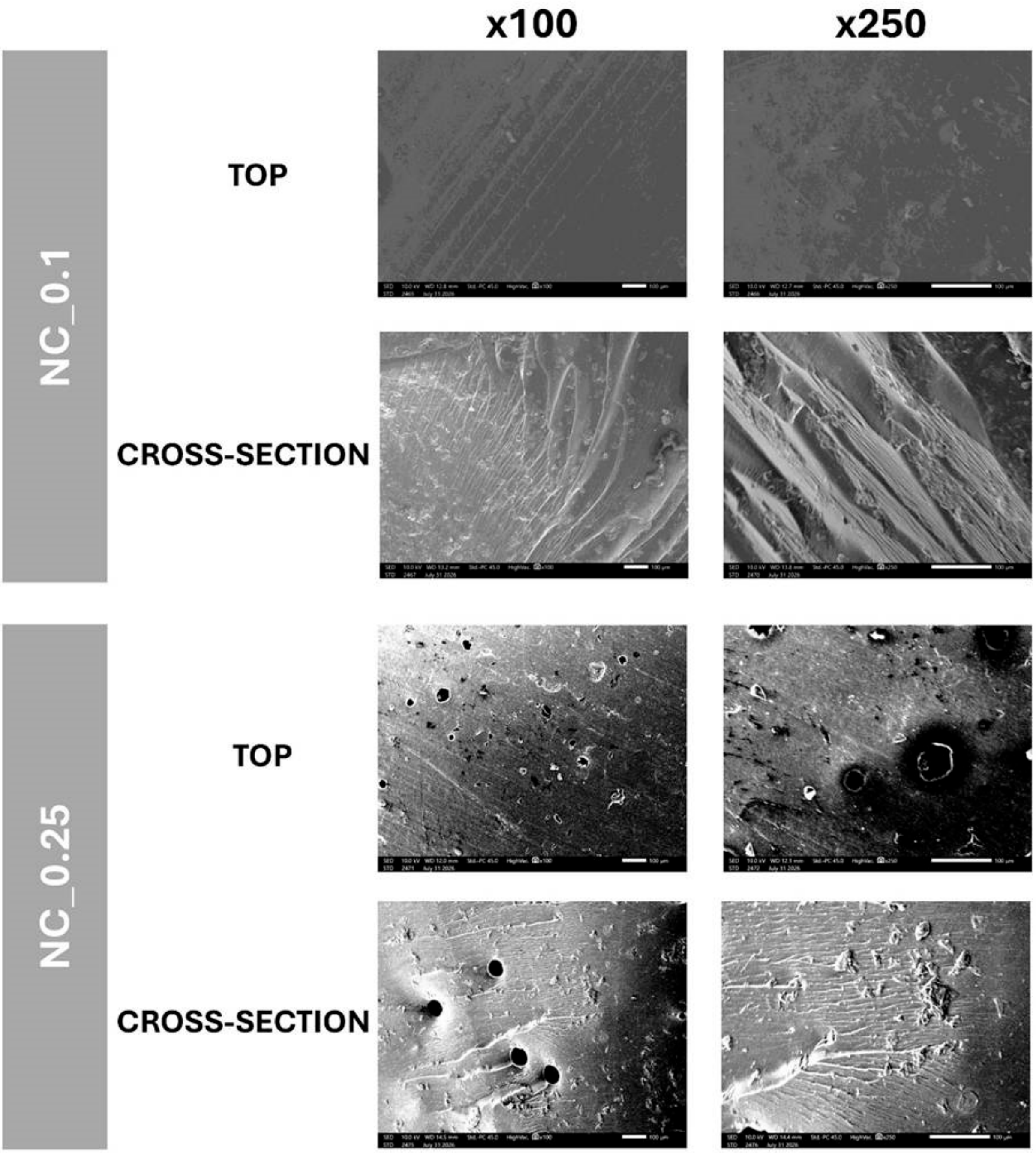

Figure S12: Representative SEM micrographs of the top surfaces and cross-sectional fracture surfaces of the neat resin and selected nanocomposites following 100 h of exposure to accelerated abiotic aging conditions: (a) Nc_0.1, (b) Nc_0.25. Micrographs were acquired at magnifications of 100× and 250×. Scale bars: 100 µm.

https://doi.org/10.3390/ma19183989

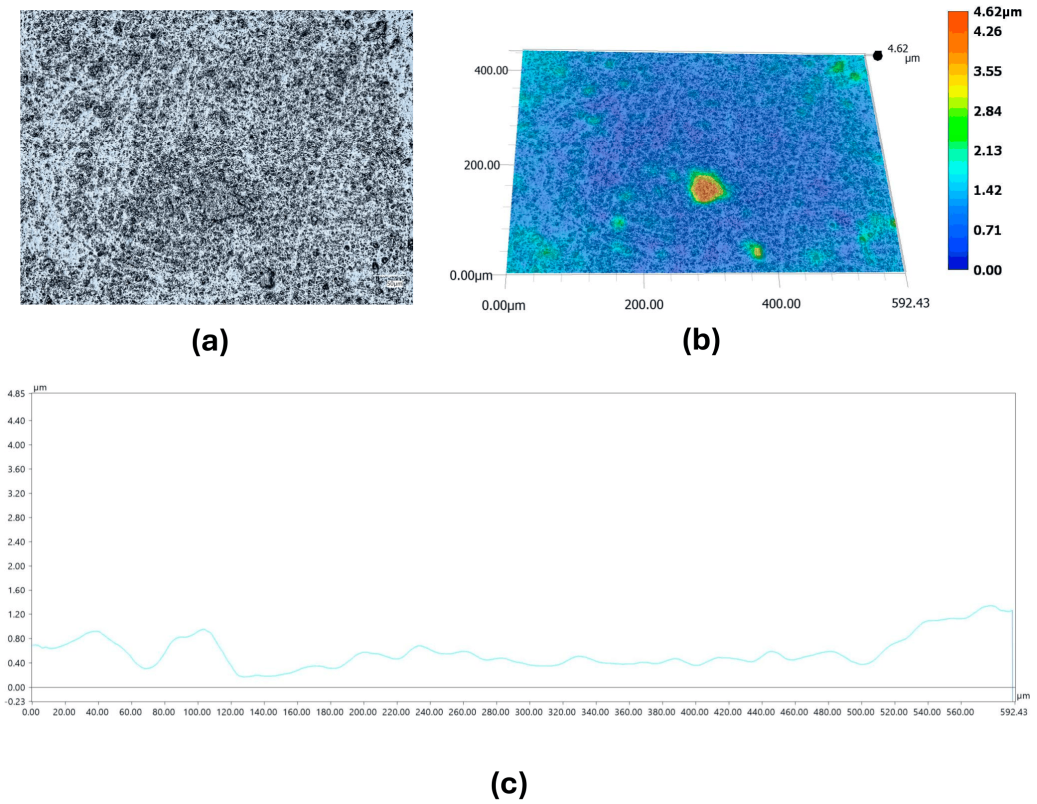

Figure S13: Surface morphology and topography of NC_0.1 after accelerated abiotic weathering test: (a) two-dimensional optical micrograph recorded at 500× magnification, (b) three-dimensional surface topography with a colour-coded height scale, and (c) representative line profile recorded across the analysed surface.

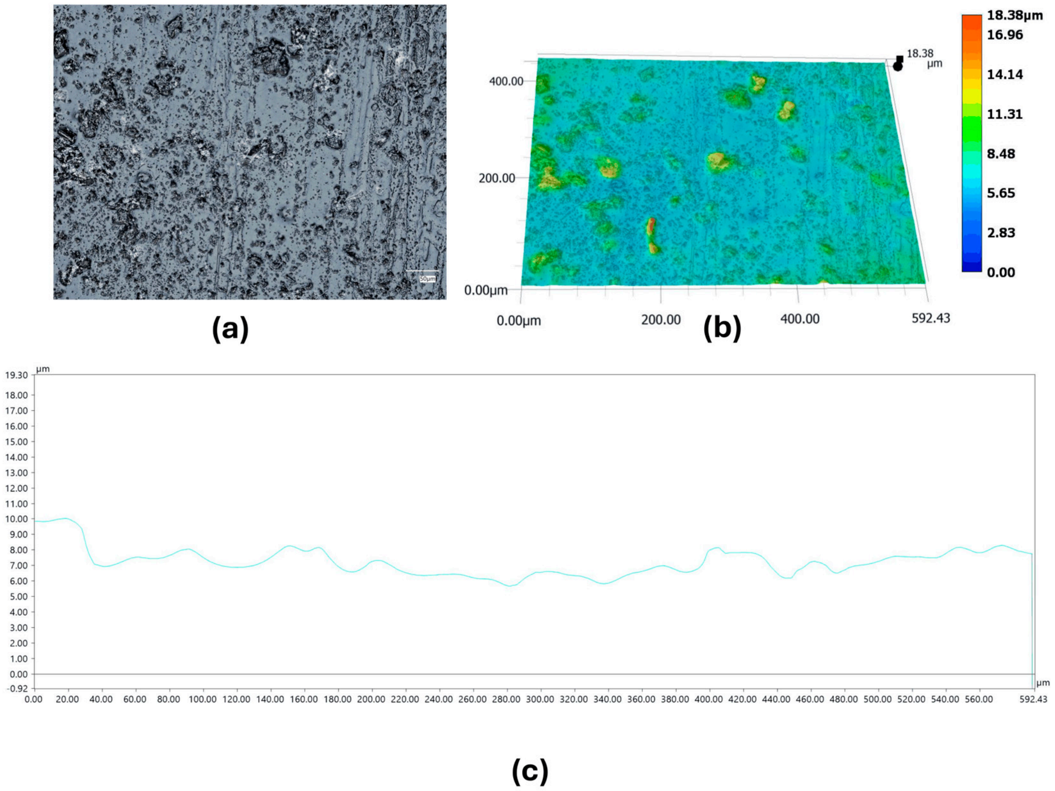

Figure S14: Surface morphology and topography of CNTs_0.1 after accelerated abiotic weathering test: (a) two-dimensional optical micrograph recorded at 500× magnification, (b) three-dimensional surface topography with a colour-coded height scale, and (c) representative line profile recorded across the analysed surface.

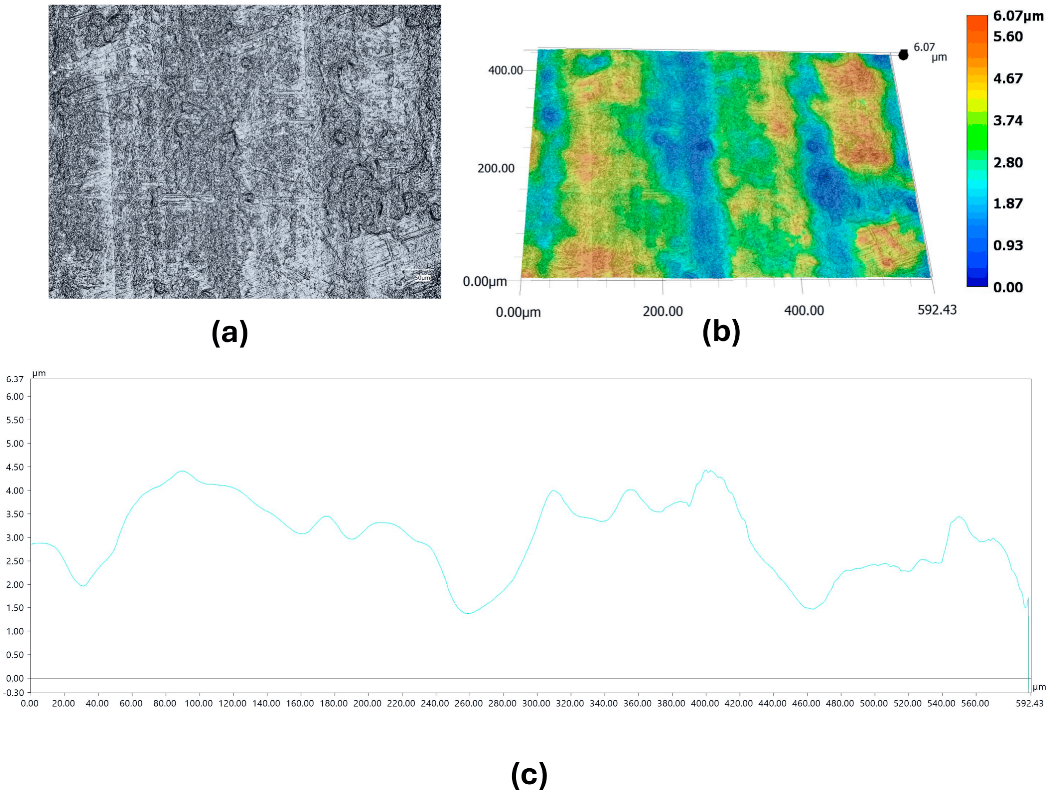

Figure S15: Surface morphology and topography of ZnO_0.1 after accelerated abiotic weathering test: (a) two-dimensional optical micrograph recorded at 500× magnification, (b) three-dimensional surface topography with a colour-coded height scale, and (c) representative line profile recorded across the analysed surface.

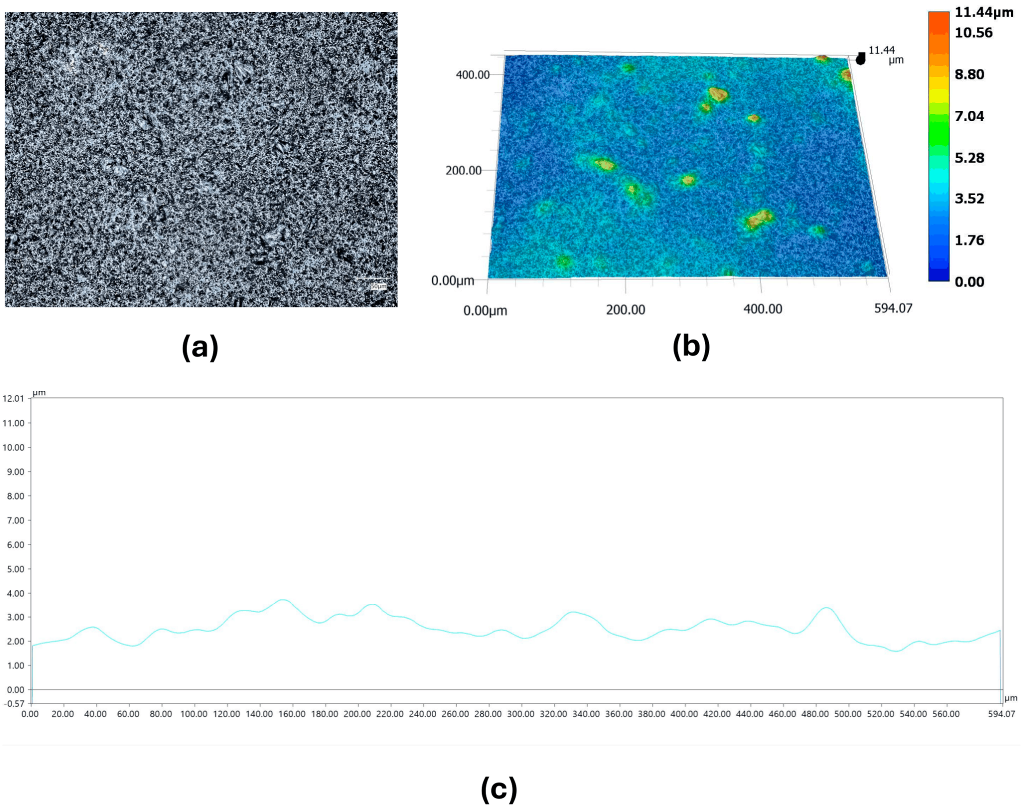

Figure S16: Surface morphology and topography of Nc_0.25 after accelerated abiotic weathering test: (a) two-dimensional optical micrograph recorded at 500× magnification, (b) three-dimensional surface topography with a colour-coded height scale, and (c) representative line profile recorded across the analysed surface.

Table S1: Areal surface texture parameters of the investigated materials after 100 h of abiotic aging. All measurements were performed with more than three replicates ($n > 3$), and the results are presented as mean ± standard error (SE). Differences were considered statistically significant, where $^*p < 0.05$.

Sample	Sa [µm]	Sz [µm]	Sq [µm]	SSk [-]	Sku [-]	Sp [µm]	Sv [µm]
Pure resin	1.91 ± 0.37	40.48 ± 8.87	3.12 ± 0.49	-1.58 ± 1.51	27.32 ± 8.65	20.02 ± 4.55	20.45 ± 5.03
Hl_0,5	1.48 ± 0.19	22.66 ± 1.24	2.43 ± 0.37	2.70 ± 0.24	22.13 ± 0.99	18.19 ± 1.33	9.48 ± 1.13
Nc_0,1	2.84 ± 0.73	44.54 ± 11.52	4.42 ± 1.14	-0.22 ± 0.84	17.97 ± 0.31	10.38 ± 1.89	34.16 ± 9.63
Nc_0,25	0.70 ± 0.00	10.94 ± 0.11	0.99 ± 0.01*	1.98 ± 0.05	11.06 ± 0.33	8.91 ± 0.23	2.04 ± 0.12
Nc_0,5	5.83 ± 1.42	50.48 ± 10.84	7.18 ± 1.70	0.97 ± 0.33	7.10 ± 1.30	23.08 ± 3.72	27.40 ± 7.12
CNTs_0,1	0.75 ± 0.01	14.90 ± 1.00	1.03 ± 0.02*	1.75 ± 0.04	5.30 ± 1.48	9.47 ± 0.43	5.43 ± 0.57
ZnO_0,1	0.94 ± 0.03	7.30 ± 0.35*	1.14 ± 0.02*	-0.14 ± 0.03	2.51 ± 0.03	3.81 ± 0.28	3.49 ± 0.07

https://doi.org/10.3390/ma19183989

Disclaimer/Publisher's Note: The statements, opinions and data contained in all publications are solely those of the individual author(s) and contributor(s) and not of MDPI and/or the editor(s). MDPI and/or the editor(s) disclaim responsibility for any injury to people or property resulting from any ideas, methods, instructions or products referred to in the content.
